# Checklist and identification key of Anomalini (Coleoptera, Scarabaeidae, Rutelinae) of Costa Rica

**DOI:** 10.3897/zookeys.621.7565

**Published:** 2016-10-03

**Authors:** Valentina Filippini, Estefanía Micó, Eduardo Galante

**Affiliations:** 1 Centro Iberoamericano de la Biodiversidad (CIBIO), Universidad de Alicante, Carretera San Vicente del Raspeig s/n 03690 San Vicente del Raspeig, Spain

**Keywords:** Identification key, new species, Anomala, Callistethus, Strigoderma, Anomalorhina, Moroniella, aedeagus, endophallus

## Abstract

A checklist and identification key for the species of the tribe Anomalini in Costa Rica are presented. The Anomalini species are important economically, as they have larvae that are or can become agricultural pests, as well as ecologically, having potential as bioindicators. In spite of their importance and richness, identification tools for the group in the Neotropics remain scarce. The Costa Rican fauna comprises six genera (*Anomala*, *Anomalorhina*, *Callistethus*, *Epectinaspis*, *Moroniella*, and *Strigoderma*) and a total of 120 species. *Anomala contusa* Filippini, Micó, Galante, 2015 is proposed as a synonym of *Anomala inbio* (Ramírez-Ponce, Bitar, Curoe 2014); *Anomala limon* **nom. n.** is proposed as a new name for *Anomala inbio* Filippini, Galante, Micó, 2015, a homonym of *Anomala inbio* (Ramírez-Ponce, Bitar, Curoe, 2014); *Anomala cinaedias* **nom. n.** is proposed as a new name for *Anomala chloropyga* Ohaus, 1897, a homonym of *Anomala chloropyga* Burmeister, 1844; and *Anomala chrysomelina* is moved to the genus *Callistethus*.

## Abstract (es)

Presentamos el listado y la clave de identificación de las especies de la tribu Anomalini de Costa Rica. Las especies de Anomalini son importantes económica, con larvas que son o pueden ser plagas agrícolas, y ecológicamente, con un potencial como bioindicadores. A pesar de su importancia y riqueza, los instrumentos de identificación para el grupo para el Neoptrópico son todavía escasos. La fauna de Costa Rica está compuesta por seis géneros (*Anomala*, *Anomalorhina*, *Callistethus*, *Epectinaspis*, *Moroniella* y *Strigoderma*) y un total de 120 especies. *Anomala contusa* Filippini, Micó, Galante, 2015 se propone como sinónimo de *Anomala inbio* (Ramírez-Ponce, Bitar, Curoe, 2014); *Anomala limon* se propone como nuevo nombre de *Anomala inbio* Filippini, Galante, Micó, 2015, homónimo de *Anomala inbio* (Ramírez-Ponce, Bitar, Curoe, 2014); *Anomala cinaedias* se propone como nuevo nombre de *Anomala chloropyga* Ohaus, 1897, homónimo de *Anomala chloropyga* Burmeister, 1844; *Anomala chrysomelina* se mueve al género *Callistethus*.

## Introduction

One reason for the “taxonomic neglect” (Jameson et al. 2003) of the genus *Anomala* over the past centuries is due to the inverse relationship between the number of species in that genus and the available taxonomic information about them. Most descriptions date to the early 20^th^ century and earlier, and the brevity of these descriptions makes reliable identification impossible without consulting the type material. The largest contribution to the study of the Anomalini in Central America was made by H. W. Bates, with his collaboration on the volumes of *Biologia Centrali-Americana* (1888 for the volume on Anomalini).

For the Neotropics, only a few national checklists that include Anomalini are available, such as Ratcliffe’s (2002) for Panama and Paucar-Cabrera’s (2005) for Ecuador. Species-level keys are available only for local fauna (e.g., Neita et al. 2006; Reyes Novelo and Morón 2005; Carrillo Ruiz and Morón 2003; Alcazar Ruiz et al. 2003).

Adults of most Anomalini species are nocturnal and are easily captured by light traps. Although they may serve as bioindicators for ecosystem conservation, the lack of proper identification tools makes such a role difficult (Morón 1997). The larvae of Anomalini are subterranean feeders, consuming roots and organic material (Ritcher 1966), and are considered ecologically important for their role in the airing of soil and decomposition of organic material (Morón and Aragón 2003). Some species are known to cause damage to crops, and some have become invasive agricultural pests in countries where they are adventive. While no invasive species of Anomalini have been recorded in Costa Rica (I3N Costa Rica http://invasoras.acebio.org), a few species of *Anomala* have been found to be associated with different crops, together with the scarab beetles *Phyllophaga* (Melolonthinae) and *Cyclocephala* species (Dynastinae). The lack of knowledge about the species’ larval morphology, however, makes it difficult to identify which species are associated with crop damage, and identification is usually done on adults collected nearby (Abarca and Quesada 1997). Larval descriptions are available for only four of the species recorded in Costa Rica (*Anomala discoidalis*, *Anomala undulata*, *Anomala ludoviciana*, *Anomala cupricollis*; Micó et al. 2003).

In this paper, a checklist for the Anomalini of Costa Rica is presented, which comprise 120 species, including photographs of the habitus and drawings of male genitalia for nearly all species, and a comprehensive key for use in identification. This work is the final part of the of a three year taxonomic study on Costa Rican Anomalini performed by the authors, which has resulted in the description of more than 50 new species of *Anomala* and *Callistethus* (Filippini et al. 2013, 2014, 2015a–e)

*Anomala contusa* Filippini, Micó Galante 2015 is proposed as a synonym of *Anomala inbio* (Ramírez-Ponce, Bitar, Curoe, 2014); *Anomala limon* is proposed as a new name for *Anomala inbio* Filippini, Galante, Micó, 2015, a homonym of *Anomala inbio* (Ramírez-Ponce, Bitar, Curoe, 2014); *Anomala cinaedias* is proposed as a new name for *Anomala chloropyga* Ohaus, 1897, homonym of *Anomala chloropyga* Burmeister, 1844; and *Anomala chrysomelina* is moved to the genus *Callistethus*.

## Methods

Specimens cited in this publication are deposited in the following collections:

BMNH
Natural History Museum, London, United Kingdom 


CEUA
 CIBIO Research Institute, Entomological Collection of the University of Alicante, Spain 


MNCR
National Museum of Costa Rica, Costa Rica 


MNHUB
Natural History Museum of Humboldt University, Germany 


MUCR
 Insect Museum, University of Costa Rica, Costa Rica 


Procedures for the preparation of endophalli, measurements, definitions, and morphological terminology follow Filippini et al. (2013, 2014). In contrast to Ramírez-Ponce and Morón (2009), who group New World *Anomala* species into a new genus (*Paranomala*) we follow the traditional inclusion in the genus *Anomala* (Jameson et al. 2003) as a more conservative classification, while waiting for a more extensive study at global scale. We use the phylogenetic species concept described by Wheeler and Platnick (2000), which defines species as the smallest aggregation of sexual populations that are diagnosable by a unique combination of character states.

Line drawings were traced using a GNU image manipulation program (GIMP version 2.8, www.gimp.org). Original drawings were produced with the aid of a camera lucida attached to a stereo microscope (Leica M80) for endophalli, or from photographs for aedeagi (taken with a Leica DFC450 camera mounted on a Leica M205C stereo microscope).

**Figure 1.**
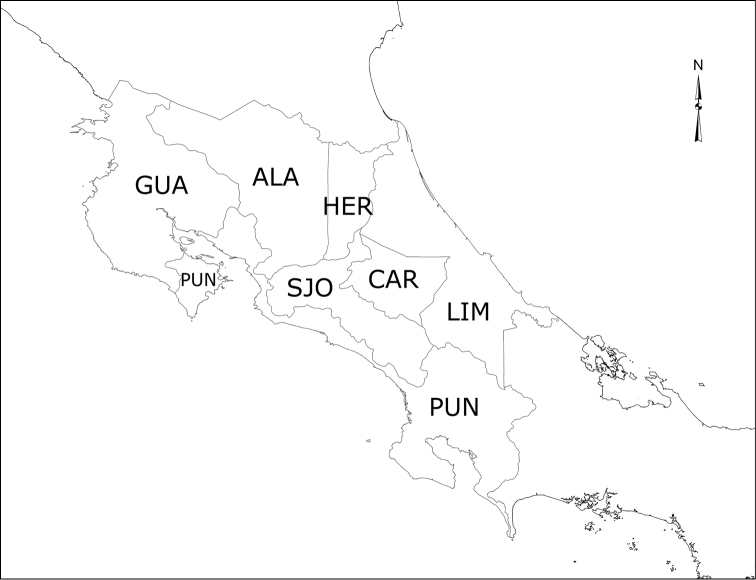
Map of Costa Rica showing provinces. ALA: Alajuela; CAR: Cartago; GUA: Guanacaste; HER: Heredia; LIM: Limón; PUN: Puntarenas; SJO: San José.

For each species in the checklist, the provinces where it is located are given in Figure 1. The distribution data were gathered using labels from identified specimens in the MNCR and CEUA collections, and the Atta database (http://atta.inbio.ac.cr/) of INBio, Costa Rica.

Species identification was undertaken by consulting original species descriptions and, when possible, by type comparison. For a list of the types consulted, see Filippini et al. (2015b). Nomenclatural changes are suggested in accordance with the International Code of Zoological Nomenclature.

## Data resources

The data from specimens deposited at CEUA are deposited at GBIF, the Global Biodiversity Information Facility, http://www.gbif.es/ic_colecciones.php?ID_Coleccion=9709

## Results

In the last three years, new species descriptions have led to a 49% increase in the known Costa Rican fauna of the tribe (Filippini et al. 2013, 2014, 2015a-e; Ramírez-Ponce et al. 2014; Ramírez-Ponce and Curoe 2014). The lack of taxonomical and faunistic studies in other Neotropical countries, however, makes it difficult to make comparisons with similar regions, or even to determine which species are endemic. For example, only 42 Anomalini species are registered for Panama (Ratcliffe 2002), and only 79 for Ecuador (which has a surface area five times that of Costa Rica) (Paucar-Cabrera 2005). It is safe to say that most of the Anomalini diversity in the Neotropical region is yet to be described.

The present work does not exhaust the actual diversity of Costa Rican Anomalini; there were at least a few species that, for reasons such as the scarcity of specimens, were not included in the descriptive work.

Based on the data gathered from the studied specimens, the richest habitats for the species were various types of evergreen tropical forests located on the slopes of the country’s main mountain ranges (unpublished material).

### Nomenclatural changes

***Anomala inbio* (Ramírez-Ponce, Bitar, Curoe, 2014), comb. n.**

*Anomala contusa* Filippini, Micó, Galante, 2015, **syn. n.**

The comparison between specimens of *Anomala contusa* and the description and illustrations of *Anomala inbio* lead to the conclusion that they are the same species. In particular, the diagnostic characteristics that include the species in the *trapezifera* species group (pronotum bronze with an irregular shaped macula, elytra with ochre background covered by small numerous flecks, tridentate protibia, tectum shorter than or similar in size to the basal piece) (Filippini et al. 2015), the particular texture of the pronotum of this species (not shared by other Costa Rican species), and the peculiar shape of aedeagus, are coincident.

*Anomala inbio* (Ramírez-Ponce, Bitar, Curoe, 2014) was originally described in the genus *Paranomala*. For the reasons explained in Methods it is here placed in the genus *Anomala*.

***Anomala limon* Filippini, Micó, Galante, nom. n.**

For *Anomala inbio* Filippini, Galante, Micó, 2015 [not *Anomala inbio* (Ramírez-Ponce, Bitar, Curoe, 2014)]

Due to the homonymy of *Anomala inbio* Filippini, Galante, Micó, 2015 with *Anomala inbio* (Ramírez-Ponce, Bitar, Curoe, 2014) (Figs 32, 145, 258), a replacement name is proposed here for the first species: *Anomala limon* (Figs 37, 150, 262). The comparison between images of the habitus and male genitalia of the two species leaves no doubt that the same name was given to very different species.

**Etymology.** this species is specific to the Costa Rican Province of Limón, where most of the type material was collected. To be used as a name in apposition.

***Anomala cinaedias* Filippini, Micó, Galante, nom. n.**

For *Anomala chloropyga* Ohaus, 1897 (not *Anomala chloropyga* Burmeister 1844).

*Anomala chloropyga* Ohaus, 1897 is a homonym for *Anomala chloropyga* Burmeister, 1844, a species from the Philippines, so a replacement name is necessary.

**Etymology.** from the Greek κιναιδίας, a precious stone, for the smooth and shiny appearance of this species. To be used as a noun in apposition.

***Callistethus chrysomelinus* (Bates, 1888), comb. n.**

*Anomala chrysomelina* Bates, 1888 is moved to the genus *Callistethus*, as it presents the following diagnostic characteristics of this genus, as described in Filippini et al. (2015): wide interocular space and small elongated eyes; posterior margin of the pronotum is smooth, without bead, and directly opposite to the scutellum; presence of a mesosternal process produced slightly beyond the apex of the mesocoxae.

### Checklist of Anomalini of Costa Rica

The three figure numbers for each species refer to the habitus, aedeagus and endophallus, respectively.

*ANOMALA* Samouelle, 1819

1. *Anomala aereiventris* Filippini, Micó, Galante, 2015d (Figs 2, 115, 228)
  Distribution: Cartago, San José.
2. *Anomala aglaos* Filippini, Galante, Micó, 2015b (Figs 3, 116, 229)
  Distribution: Alajuela, San José.
3. *Anomala antica* Ohaus, 1897 (Figs 4, 117, 230)
  Note: type specimens (Colombia, MNHUB) have aedeagus with longer and thinner parameres than specimens from Costa Rica.
  Distribution: Alajuela, Guanacaste, Limón, Puntarenas, San José.
4. *Anomala arara* Ohaus, 1897 (Figs 5, 118, 231)
  Distribution: Alajuela, Cartago, Guanacaste, Puntarenas, San José.
5. *Anomala arthuri* Filippini, Micó, Galante, 2014 (Figs 6, 119, 232)
  Distribution: Guanacaste.
6. *Anomala aspersa* Filippini, Micó, Galante, 2015d (Figs 7, 120, 233)
  Distribution: Cartago, San José.
7. *Anomala atrivillosa* Filippini, Micó, Galante, 2015d (Figs 8, 121, 234)
  Distribution: Heredia.
8. *Anomala balzapambae* Ohaus, 1897 (Figs 9, 122, 235)
  Distribution: Alajuela, Guanacaste, Heredia, Limón, Puntarenas, San José.
9. *Anomala calligrapha* Bates, 1888 (Figs 10, 123, 236)
  Distribution: Alajuela, Cartago, Guanacaste, Limón, Puntarenas, San José.
10. *Anomala chiriquina* Bates, 1888 (Figs 11, 124, 237)
  Distribution: Alajuela, Cartago, Guanacaste, Puntarenas, San José.
11. *Anomala cinaedias* **nom. n.** (Figs 12, 125, 238)
  Distribution: San José.
12. *Anomala clarivillosa* Filippini, Micó, Galante, 2015d (Figs 13, 126, 239)
  Distribution: Cartago.
13. *Anomala clathrata* Ohaus, 1930 (Figs 14, 127, 240)
  Distribution: Alajuela, Cartago, Guanacaste, Heredia, Limón, Puntarenas.
14. *Anomala coffea* Filippini, Galante, Micó, 2015c (Figs 15, 128, 241)
  Distribution: Guanacaste.
15. *Anomala cupreovariolosa* Filippini, Micó, Galante, 2014 (Figs 16, 129, 242)
  Distribution: Puntarenas.
16. *Anomala cupricollis* Chevrolat, 1834 (Figs 17, 130, 243)
  Distribution: Alajuela, Cartago, Guanacaste, Heredia, Limón, Puntarenas.
17. *Anomala cyclops* Filippini, Galante, Micó, 2015c (Figs 18, 131, 244)
  Distribution: Guanacaste.
18. *Anomala discoidalis* Bates, 1888 (Figs 19, 132, 245)
  Distribution: Alajuela, Cartago, Guanacaste, Heredia, Limón, Puntarenas, San José.
19. *Anomala divisa* Filippini, Galante, Micó, 2015c (Figs 20, 133, 246)
  Distribution: Alajuela, Puntarenas.
20. *Anomala estrella* Filippini, Galante, Micó, 2015b (Figs 21, 134, 247)
  Distribution: Guanacaste, Puntarenas.
21. *Anomala eucoma* Bates, 1888 (Figs 22, 135, 248)
  Distribution: Guanacaste, Limón, Puntarenas.
22. *Anomala eulissa* Bates, 1888 (Figs 23, 136, 249)
  Distribution: Alajuela, Cartago, Guanacaste, Heredia, Limón, Puntarenas.
23. *Anomala eusticta* Filippini, Micó, Galante, 2015d (Figs 24, 137, 250)
  Distribution: Guanacaste, Puntarenas.
24. *Anomala ferrea* Filippini, Micó, Galante, 2014 (Figs 25, 138, 251)
  Distribution: Puntarenas.
25. *Anomala flavacoma* Filippini, Micó, Galante, 2013 (Figs 26, 139, 252)
  Distribution: Alajuela, Guanacaste, Limón.
26. *Anomala foraminosa* Bates, 1888 (Figs 27, 140, 253)
  Distribution: Guanacaste, Heredia, Limón, Puntarenas, San José.
27. *Anomala globulata* Filippini, Micó, Galante, 2015d (Figs 28, 141, 254)
  Distribution: Cartago, San José.
28. *Anomala hiata* Filippini, Micó, Galante, 2015d (Figs 29, 142, 255)
  Distribution: Puntarenas.
29. *Anomala histrionella* Bates, 1888 (Figs 30, 143, 256)
  Distribution: Alajuela, Guanacaste, Puntarenas.
30. *Anomala hoppi* Ohaus, 1928 (Figs 31, 144, 257)
  Distribution: Alajuela, Cartago, Guanacaste, Limón, Puntarenas.
31. *Anomala inbio* (Ramírez-Ponce, Bitar, Curoe, 2014) (Figs 32, 145, 258)
  Distribution: Guanacaste, San José.
32. *Anomala jansoni* Ohaus, 1897 (Figs 33, 146)
  Note: no specimens apart from the type series are known.
  Distribution: “Monte Rotondo, Costa Rica”.
33. *Anomala latifalculata* Filippini, Micó, Galante, 2015d (Figs 34, 147, 259)
  Distribution: Cartago.
34. *Anomala leopardina* Filippini, Micó, Galante, 2015d (Figs 35, 148, 260)
  Distribution: Puntarenas.
35. *Anomala levicollis* Filippini, Micó, Galante, 2015d (Figs 36, 149, 261)
  Distribution: Alajuela, Cartago, Guanacaste, Puntarenas.
36. *Anomala limon* **nom. n.** (Figs 37, 150, 262)
  Distribution: Heredia, Limón.
37. *Anomala longisacculata* Filippini, Micó, Galante, 2015d (Figs 38, 151, 263)
  Distribution: Alajuela, Cartago, Guanacaste, Limón, San José.
38. *Anomala ludoviciana* Schaeffer, 1906 (Figs 39, 152, 264)
  Distribution: Guanacaste, Puntarenas.
39. *Anomala megalia* Bates, 1888 (Figs 40, 153, 265)
  Distribution: Limón, San José.
40. *Anomala megaparamera* Filippini, Micó, Galante, 2013 (Figs 41, 154, 266)
  Distribution: Limón.
41. *Anomala mersa* Filippini, Galante, Micó, 2015c (Figs 42, 155, 267)
  Distribution: Guanacaste.
42. *Anomala mesosticta* Filippini, Galante, Micó, 2015c (Figs 43, 156, 268)
  Distribution: Heredia, Limón.
43. *Anomala m-fuscum* Filippini, Micó, Galante, 2015d (Figs 44, 157, 269)
  Distribution: Cartago.
44. *Anomala moroni* Filippini, Micó, Galante, 2015e (Figs 45, 158, 270)
  Distribution: Guanacaste.
45. *Anomala nigroflava* Filippini, Micó, Galante, 2014 (Figs 46, 159, 271)
  Distribution: Puntarenas.
46. *Anomala obovata* Ohaus, 1933 (Figs 47, 160, 272)
  Distribution: Cartago, Heredia, Limón.
47. *Anomala ochrogastra* Bates, 1888 (Figs 48, 161, 273)
  Distribution: Guanacaste, Heredia, Limón, Puntarenas.
48. *Anomala ochroptera* Bates, 1888 (Figs 49, 162, 274)
  Distribution: Guanacaste, Puntarenas.
49. *Anomala parvaeucoma* Filippini, Micó, Galante, 2015e (Figs 50, 163, 275)
  Distribution: Puntarenas.
50. *Anomala perspicax* Filippini, Micó, Galante, 2015d (Figs 51, 164, 276)
  Distribution: Cartago, Puntarenas.
51. *Anomala piccolina* Filippini, Micó, Galante, 2015d (Figs 52, 165, 277)
  Distribution: Puntarenas.
52. *Anomala pincelada* Filippini, Galante, Micó, 2015b (Figs 53, 166, 278)
  Distribution: Guanacaste.
53. *Anomala polygona* Bates, 1888 (Figs 54, 167)
  Note: apart from the type specimen, only one recent specimen with dubious identification is known.
  Distribution: “Costa Rica” (holotype, MNHN); San José (1 specimen at MNHUB); Limón (see Filippini et al. 2014).
54. *Anomala popayana* Ohaus, 1897 (Figs 55, 168, 279)
  Distribution: Guanacaste, Heredia, Limón, Puntarenas.
55. *Anomala praecellens* Bates, 1888 (Figs 56, 169, 280)
  Distribution: Alajuela, Cartago, Guanacaste, Heredia, Limón, Puntarenas, San José.
56. *Anomala pseudoeucoma* Filippini, Micó, Galante, 2013 (Figs 57, 170, 281)
  Distribution: Alajuela, Limón, Puntarenas.
57. *Anomala quiche* Ohaus, 1897 (Figs 58, 171, 282)
  Note: specimens from Costa Rica differ from the type specimen (Guatemala, MNHUB) in that they have two defined maculae on the pronotum instead of one. Aedeagus is identical.
  Distribution: Alajuela, Guanacaste, Heredia, Limón, Puntarenas.
58. *Anomala robiginosa* Filippini, Galante, Micó, 2015c (Figs 59, 172, 283)
  Distribution: Alajuela, Guanacaste.
59. *Anomala ruatana* Bates, 1888 (Figs 60, 173, 284)
  Distribution: Guanacaste.
60. *Anomala semicincta* Bates, 1888 (Figs 61, 174, 285)
  Distribution: Alajuela, Cartago, Guanacaste, Heredia, Limón, Puntarenas.
61. *Anomala semilla* Filippini, Micó, Galante, 2014 (Figs 62, 175, 286)
  Distribution: Alajuela, Guanacaste.
62. *Anomala solisi* Filippini, Micó, Galante, 2014 (Figs 63, 176, 287)
  Distribution: Alajuela, Guanacaste, Limón.
63. *Anomala stillaticia* Filippini, Micó, Galante, 2015d (Figs 64, 177, 288)
  Distribution: Cartago.
64. *Anomala strigodermoides* Filippini, Galante, Micó, 2015c (Figs 65, 178, 289)
  Distribution: Alajuela, Cartago.
65. *Anomala subaenea* (Nonfried, 1893) (Figs 66, 179, 290)
  Distribution: Guanacaste.
66. *Anomala subridens* Filippini, Micó, Galante, 2015d (Figs 67, 180, 291)
  Distribution: Cartago.
67. *Anomala subusta* Filippini, Micó, Galante, 2015d (Figs 68, 181, 292)
  Distribution: Alajuela, Cartago, Guanacaste, Puntarenas.
68. *Anomala tenoriensis* Filippini, Micó, Galante, 2015d (Figs 69, 182, 293)
  Distribution: Alajuela, Guanacaste.
69. *Anomala testaceipennis* Blanchard, 1851 (Figs 70, 183, 294)
  Distribution: Alajuela, Cartago, Guanacaste, Heredia, Limón, Puntarenas, San José.
70. *Anomala trapezifera* Bates, 1888 (Figs 71, 184, 295)
  Distribution: Cartago, Limón.
71. *Anomala tuberculata* Filippini, Micó, Galante, 2015d (Figs 72, 185, 296)
  Distribution: Alajuela, Cartago, San José.
72. *Anomala undulata* Melsheimer, 1844 (Figs 73, 186, 297)
  Distribution: Alajuela, Cartago, Guanacaste, Heredia, Limón, Puntarenas, San José.
73. *Anomala unilineata* Filippini, Galante, Micó, 2015c (Figs 74, 187, 298)
  Distribution: Guanacaste.
74. *Anomala valida* Burmeister, 1844 (Figs 75, 188, 299)
  Distribution: Alajuela, Cartago, Guanacaste, Heredia, Limón, Puntarenas, San José.
75. *Anomala vallisneria* Filippini, Micó, Galante, 2015d (Figs 76, 189, 300)
  Distribution: Alajuela, Cartago, Guanacaste, Puntarenas.
76. *Anomala veraecrucis* Bates, 1888 (Figs 77, 190, 301)
  Distribution: Alajuela, Cartago, Guanacaste, Puntarenas.
77. *Anomala volsellata* Filippini, Micó, Galante, 2014 (Figs 78, 191, 302)
  Distribution: Puntarenas, San José.
78. *Anomala vulcanicola* Ohaus, 1897 (Figs 79, 192, 303)
  Distribution: San José.
79. *Anomala zumbadoi* Filippini, Micó, Galante, 2014 (Figs 80, 193, 304)
  Distribution: Puntarenas.

*ANOMALORHINA* Jameson, Paucar-Cabrera, Solís, 2003

1. *Anomalorhina osaensis* Jameson, Paucar-Cabrera, Solís, 2003
  Distribution: Puntarenas.
2. *Anomalorhina turrialbana* (Ohaus, 1928) (Figs 81, 194, 305)
  Distribution: Alajuela, Cartago.

*CALLISTETHUS* Blanchard, 1851

1. *Callistethus calonotus* (Bates, 1888) (Figs 82, 195, 306)
  Distribution: Puntarenas.
2. *Callistethus carbo* Filippini, Galante, Micó, 2015a (Figs 83, 196, 307)
  Distribution: Guanacaste.
3. *Callistethus chlorotoides* (Bates, 1888) (Figs 84, 197, 308)
  Distribution: Alajuela, Cartago, Limón, Puntarenas, San José.
4. *Callistethus chontalensis* (Bates, 1888) (Figs 85, 198, 309)
  Distribution: Alajuela, Cartago, Guanacaste, Heredia, Limón, Puntarenas.
5. *Callistethus chrysanthe* (Bates, 1888) (Figs 86, 199)
  Note: no specimens apart from the type series are known.
  Distribution: “Costa Rica”.
6. *Callistethus chrysomelinus* (Bates, 1888) (Figs 87, 200, 310)
  Distribution: Puntarenas.
7. *Callistethus flavodorsalis* Filippini, Galante, Micó, 2015a (Figs 88, 201, 311)
  Distribution: Puntarenas.
8. *Callistethus fuscorubens* Filippini, Galante, Micó, 2015a (Figs 89, 202, 312)
  Distribution: Puntarenas.
9. *Callistethus granulipygus* (Bates, 1888) (Figs 90, 203, 313)
  Distribution: Alajuela, Guanacaste, Heredia, Limón, Puntarenas, San José.
10. *Callistethus jordani* (Ohaus, 1902) (Figs 91, 204, 314)
  Distribution: Guanacaste, Puntarenas.
11. *Callistethus lativittis* Filippini, Galante, Micó, 2015a (Figs 92, 205, 315)
  Distribution: Alajuela, Guanacaste.
12. *Callistethus levigatus* Filippini, Galante, Micó, 2015a (Figs 93, 206, 316)
  Distribution: Alajuela, Cartago, Guanacaste, Puntarenas.
13. *Callistethus macroxantholeus* Filippini, Galante, Micó, 2015a (Figs 94, 207, 317)
  Distribution: Alajuela, Limón, Guanacaste.
14. *Callistethus microxantholeus* Filippini, Galante, Micó, 2015a (Figs 95, 208, 318)
  Distribution: Alajuela, Guanacaste, Heredia, Limón, Puntarenas.
15. *Callistethus mimeloides* (Ohaus, 1902) (Figs 96, 209, 319)
  Distribution: Alajuela, Cartago, Guanacaste, Heredia, Limón, Puntarenas, San José.
16. *Callistethus multiplicatus* Filippini, Galante, Micó, 2015a (Figs 97, 210, 320)
  Distribution: Alajuela, Guanacaste, Limón.
17. *Callistethus nicoya* (Ohaus, 1928) (Figs 98, 211, 321)
  Distribution: Alajuela, Heredia, Puntarenas, San José.
18. *Callistethus parapulcher* Filippini, Galante, Micó, 2015a (Figs 99, 212, 322)
  Distribution: Guanacaste, Puntarenas, San José.
19. *Callistethus pseudocollaris* Filippini, Galante, Micó, 2015a (Figs 100, 213, 323)
  Distribution: Puntarenas.
20. *Callistethus ruteloides* Filippini, Galante, Micó, 2015b (Figs 101, 214, 324)
  Distribution: Alajuela, Cartago, Puntarenas.
21. *Callistethus schneideri* (Ohaus, 1905) (Figs 102, 215, 325)
  Distribution: Alajuela, Guanacaste, Heredia, Limón, Puntarenas, San José.
22. *Callistethus specularis* (Bates, 1888) (Figs 103, 216, 326)
  Distribution: Alajuela, Cartago, Guanacaste, Heredia, Puntarenas, San José.
23. *Callistethus stannibractea* Filippini, Galante, Micó, 2015a (Figs 104, 217, 327)
  Distribution: Heredia.
24. *Callistethus sulcans* (Bates, 1888) (Figs 105, 218, 328)
  Distribution: Alajuela, Guanacaste, Limón.
25. *Callistethus valdecostatus* (Bates, 1888) (Figs 106, 219, 329)
  Distribution: Puntarenas, San José.
26. *Callistethus vanpatteni* (Bates, 1888) (Figs 107, 220, 330)
  Distribution: Alajuela, Guanacaste, Heredia, Puntarenas.
27. *Callistethus xiphostethus* (Bates, 1888) (Figs 108, 221, 331)
  Distribution: Alajuela, Guanacaste, Heredia, San José.
28. *Callistethus yalizo* Filippini, Galante, Micó, 2015b (Figs 109, 222, 332)
  Distribution: Alajuela, Cartago, Heredia.

*EPECTINASPIS* Blanchard, 1851

1. *Epectinaspis costaricensis* Ramírez-Ponce and Curoe, 2014
  Distribution: Heredia.

*MORONIELLA* Ramírez-Ponce, 2015

1. *Moroniella nitidula* (Blanchard, 1851) (Figs 110, 223, 333)
  Distribution: Alajuela, Cartago, Guanacaste, Heredia, San José.

*STRIGODERMA* Burmeister, 1844

1. *Strigoderma angulicollis* Ohaus, 1915
  Distribution: Limón.
2. *Strigoderma auriventris* Bates, 1888 (Figs 111, 224, 334)
  Distribution: Alajuela, Guanacaste, Limón.
3. *Strigoderma biolleyi* Ohaus, 1908 (Figs 112, 225, 335)
  Distribution: Cartago, Puntarenas, San José.
4. *Strigoderma castor* (Newman, 1838)
  Distribution: Heredia, San José.
5. *Strigoderma marginata* (Olivier, 1789)
  Distribution: Alajuela, Cartago, Guanacaste, Heredia, Limón, Puntarenas, San José.
6. *Strigoderma micans* Nonfried, 1893
  Distribution: Guanacaste.
7. *Strigoderma nodulosa* Ohaus, 1902 (Figs 113, 226, 336)
  Distribution: Heredia, Limón, Puntarenas.
8. *Strigoderma orbicularis* Burmeister, 1855
  Distribution: Cartago, Guanacaste, Heredia, Limón, Puntarenas.
9. *Strigoderma rutelina* Bates, 1888
  Distribution: Alajuela, Puntarenas.
10. *Strigoderma sulcipennis* Burmeister, 1844 (Figs 114, 227, 337)
  Distribution: Guanacaste, Puntarenas, San José.
11. *Strigoderma vestita* Burmeister, 1844
  Distribution: Alajuela, Guanacaste, Puntarenas, San José.

### Key to Anomalini species of Costa Rica

Partly modified from: Jameson, Paucar-Cabrera and Solís (2003), Filippini et al. (2013, 2015a, d).

**Table:** 

1	Mesepimeron is well-exposed anterior to base of elytron in dorsal view (Fig. 338B); body shape elongated	**2**
–	Mesepimeron is concealed by base of elytron or weakly exposed (Fig. 338A); body shape oval or elongated	**3**
2	Mesosternal intercoxal region subequal in width to base of mesofemur; mesepimeron subrectangular and well-exposed; clypeus of male narrowly reflexed at apex; dorsal surface of elytron flat; pronotum narrower than base of elytra (except in *Strigoderma orbicularis*)	***STRIGODERMA* Burmeister, 1844**...**7**
–	Mesosternal intercoxal region less than 1/4 width of base of mesofemur; mesepimeron subtriangular and partially exposed; clypeus of male broadly reflexed at apex; dorsal surface of elytron evenly rounded; pronotum as wide as base of elytra	***Epectinaspis costaricensis* Ramírez-Ponce & Curoe, 2014**
3	Frontoclypeal suture incomplete, sides of clypeus elevated at base of canthus; males with pronotal disc with depression; females with apical bead of pronotum produced posteriorly at middle	***ANOMALORHINA* Jameson, Paucar-Cabrera, Solís, 2003**...**17**
–	Frontoclypeal suture complete, sides of clypeus weakly elevated or flat at base of canthus; males with pronotal disc evenly convex; females with apical bead of pronotum not produced posteriorly	**4**
4	Surface between mesocoxae not produced beyond mesotrochanters; pronotum with basal bead complete or obsolete at middle	***ANOMALA* Samouelle, 1819**...**18**
–	Surface between mesocoxae possessing a mesosternal process and produced beyond mesotrochanters; pronotum with basal bead or lacking basal bead	**5**
5	Parameres perpendicular to the phallobase; metatarsi 1-4 together similar in length to the 5th, excluding claws; small size	***Moroniella nitidula* (Blanchard, 1851)**
–	Parameres in line with the phallobase; metatarsi 1-4 together longer than the 5th, excluding claws; size varies	**6**
6	Mesosternal process short, either not or slightly produced beyond the mesocoxae, apex seen as a bump in lateral view	***ANOMALA* Samouelle, 1819** and ***CALLISTETHUS* Blanchard, 1851**...**96**
–	Mesosternal process long, produced beyond the mesocoxae for more than half the width of mesocoxa, apex free in lateral view	***CALLISTETHUS* Blanchard, 1851**...**112**
7	Pronotum with irregular surface, granulate or with concavities	**8**
–	Pronotum homogeneously convex	**10**
8	Pronotum with granulate surface	***Strigoderma nodulosa* Ohaus, 1902**
–	Pronotum with concavities	**9**
9	Body length 11–13 mm; pronotum homogeneously black or copper; elongated shape (elytra > 2 times longer than wide)	***Strigoderma sulcipennis* Burmeister, 1844**
–	Body length 7–9 mm; pronotum green or reddish brown with white sides; rounded shape (elytra < 2 times longer than wide)	***Strigoderma castor* (Newman, 1838)**
10	Elytra smooth; venter with metallic colors	***Strigoderma auriventris* Bates, 1888**
–	Elytra striated; venter not with metallic colors	**11**
11	Pronotum with green metallic color	**12**
–	Pronotum brown to black, without metallic luster	**14**
12	Elytra black with a ochre circle near base	***Strigoderma rutelina* Bates, 1888**
–	Elytra with uniform color	**13**
13	Pronotum with one central macula or uniformly colored; elytra black or brown	***Strigoderma biolleyi* Ohaus, 1908**
–	Pronotum with two lateral light colored bands; elytra brown	***Strigoderma micans* Nonfried, 1893**
14	Elytra homogeneously brown	***Strigoderma vestita* Burmeister, 1844**
–	Elytra black or brown with black maculae	**15**
15	Body length 11–12 mm	***Strigoderma angulicollis* Ohaus, 1915**
–	Body length < 8 mm	**16**
16	Rounded shape; pronotum strongly convex, as wide as base of elytra; body length 7–8 mm	***Strigoderma orbicularis* Burmeister, 1855**
–	Elongated shape; pronotum slightly convex, narrower than base of elytra; body length 5–7 mm	***Strigoderma marginata* (Olivier, 1789)**
17	Head and pronotum rufous, elytra black or reddish brown; male with two tubercles on base of frons; clypeus with apex acute in frontal view; females with subsutural interstice twice as wide as first costa, frontal disc slightly concave	***Anomalorhina turrialbana* (Ohaus, 1928)**
–	Head, pronotum and elytra castaneous; male without tubercles on frons; clypeus with apex quadrate in frontal view; females with subsutural interstice as wide as first costa, frontal disc slightly convex	***Anomalorhina osaensis* Jameson, Paucar-Cabrera, Solís, 2003**
18	Protibia tridentate	**19**
–	Protibia bidentate	**49**
19	Elytra of homogeneous color	**20**
–	Elytra with darker maculae	**22**
20	Body color dark brown	***Anomala coffea* Filippini, Galante, Micó, 2015**
–	Pronotum dark brown or green, elytra ochre	**21**
21	Pronotum of homogeneous color, green or brown; body length 11.0–13.0 mm; aedeagus Fig. 183	***Anomala testaceipennis* Blanchard, 1851**
–	Pronotum dark brown with ochre sides; body length 8.5–9.5 mm; aedeagus Fig. 190	***Anomala veraecrucis* Bates, 1888**
22	Head and pronotum entirely black; elytra ochre with large irregular black maculae developing longitudinally (Fig. 53)	***Anomala pincelada* Filippini, Galante, Micó, 2015**
–	Head and pronotum metallic green or brown, pronotum usually with light colored margins; elytra with small maculae on transversal bands or sparse flecks on entire surface	**23**
23	Pattern on elytra consists of one central macula or median transversal band	**24**
–	Pattern on elytra consists of various bands of maculae or sparse flecks	**25**
24	Pronotum mainly dark brown with narrow ochre sides; body length 10–12 mm; aedeagus Fig. 156	***Anomala mesosticta* Filippini, Galante, Micó, 2015**
–	Pronotum with pentagonal central dark macula, less than half the pronotum width; body length 8–10 mm; aedeagus Fig. 131	***Anomala cyclops* Filippini, Galante, Micó, 2015**
25	Pronotum with deep concavities	***Anomala inbio* (Ramírez-Ponce, Bitar, Curoe, 2014)**
–	Pronotum with convex surface	**26**
26	Presence of setae on pronotum and elytra	**27**
–	Pronotum and elytra glabrous, pronotum may have a row of a few setae	**29**
27	Body length 10.0–11.5 mm; pronotum with dense punctation; elytra with two regular rows of maculae; aedeagus in Fig. 148	***Anomala leopardina* Filippini, Micó, Galante, 2015**
–	Body length 12.0–14.0 mm; pronotum with sparse and coarse punctation; elytra irregularly covered with maculae and flecks; aedeagus with different shape	**28**
28	Light color; pronotum with evident sinuate lateral margins; aedeagus Fig. 126	***Anomala clarivillosa* Filippini, Micó, Galante, 2015**
–	Dark color; pronotum with slightly sinuate lateral margins; aedeagus Fig. 121	***Anomala atrivillosa* Filippini, Micó, Galante, 2015**
29	Lateral margins of pronotum sinuate	**30**
–	Lateral margins of pronotum regularly convex or angulated	**37**
30	Side of pronotum deeply sinuate (crosses an imaginary line from apical to basal angle); pronotum almost completely dark in color; aedeagus Fig. 167	***Anomala polygona* Bates, 1888**
–	Lateral margins of pronotum weakly sinuate (do not cross an imaginary line from apical to basal angle); pronotum with an irregular macula on disc or ochre margins; aedeagus with different shape	**31**
31	Pronotum dark with ochre margins; elytra with regular maculae; aedeagus Fig. 142	***Anomala hiata* Filippini, Micó, Galante, 2015**
–	Pronotum with an irregular macula on its disc; elytra with several small flecks; aedeagus with different shape	**32**
32	Pronotum with a narrow sinuate macula; elytra with few flecks; body length less than 11.5 mm; parameres long with an acute apex and protruding ventral angle in lateral view (Fig. 157)	***Anomala m-fuscum* Filippini, Micó, Galante, 2015**
–	Pronotum with larger maculae; elytra with abundant flecks; body length 10.2–13.4 mm; different aedeagus	**33**
33	Ventral plate with elongated apical corners; short parameres, less than half the length of the tectum (Fig. 192)	***Anomala vulcanicola* Ohaus, 1897**
–	Ventral plate with a curved apex in ventral view; parameres longer than half the length of the tectum	**34**
34	Ventral plate with apical side curved in lateral view	**35**
–	Ventral plate with apical side flat	**36**
35	Parameres short with blunt apex in lateral view (Fig. 120)	***Anomala aspersa* Filippini, Micó, Galante, 2015**
–	Parameres long with acute apex in lateral view (Fig. 184)	***Anomala trapezifera* Bates, 1888**
36	Parameres long and narrow, dorsal margin curved, ventral angle obtuse (Fig. 180)	***Anomala subridens* Filippini, Micó, Galante, 2015**
–	Parameres short and wide, dorsal margin sinuate, ventral angle pointing backwards in a lateral view (Fig. 189)	***Anomala vallisneria* Filippini, Micó, Galante, 2015**
37	Elytra light colored with sparse flecks; body length > 14.1 mm	**38**
–	Elytra light colored and body length < 13.5 mm; or dark elytra and body length > 14.1 mm	**40**
38	Presence of protuberance on clypeus; aedeagus Fig. 185	***Anomala tuberculata* Filippini, Micó, Galante, 2015**
–	Clypeus with even surface; aedeagus with different shape	**39**
39	Pronotum with irregular macula on its disc; elytra with large maculae; aedeagus Fig. 137	***Anomala eusticta* Filippini, Micó, Galante, 2015**
–	Pronotum completely dark; elytra with small flecks; aedeagus Fig. 115	***Anomala aereiventris* Filippini, Micó, Galante, 2015**
40	Pronotum surface smooth	**41**
–	Pronotum surface with evident punctures	**42**
41	Pronotum completely dark; venter homogeneously bronze brown, metallic; third tooth of protibia weakly developed; body length > 13.2 mm; aedeagus Fig. 115	***Anomala aereiventris* Filippini, Micó, Galante 2015**
–	Pronotum with ochre sides; venter brown with yellowish parts, without metallic luster; third tooth of protibia well developed; body length < 13.0 mm; aedeagus Fig. 149	***Anomala levicollis* Filippini, Micó, Galante, 2015**
42	Male with large eyes (interocular ratio < 1.8); medium size	**43**
–	Male with small eyes (interocular ratio > 2.2); medium and large size	**44**
43	Pronotum with coarse punctures, basal half of lateral margins parallel; elytra with small irregularly placed flecks; aedeagus Fig. 164	***Anomala perspicax* Filippini, Micó, Galante, 2015**
–	Pronotum with fine punctures, basal half of lateral margins oblique to the base; elytra with large maculae organized in three rows; aedeagus Fig. 177	***Anomala stillaticia* Filippini, Micó, Galante, 2015**
44	Body length > 13.0 mm, body width > 7 mm; elytra nearly completely dark	**45**
–	Body length < 12.6 mm, body width < 6.8 mm; elytra usually with abundant light flecks	**46**
45	Green pronotum; elongated ochre maculae on elytra; light colored pygidium; aedeagus Fig. 182	***Anomala tenoriensis* Filippini, Micó, Galante, 2015**
–	Brown pronotum; diffuse ochre flecks on elytra; pygidium with large dark maculae; aedeagus Fig. 181	***Anomala subusta* Filippini, Micó, Galante, 2015**
46	Basal half of lateral margins of pronotum parallel; body length ≤ 10.0 mm, body width < 5.2 mm	**47**
–	Basal half of lateral margins of pronotum oblique to the base; body length generally > 11.0 mm, body width > 5.9 mm	**48**
47	Presence of concavity on frons; dark venter; micropunctation on elytra surface; male internal protarsal claw wide (upper branch 1/4 the width of the lower one); aedeagus Fig. 147	***Anomala latifalculata* Filippini, Micó, Galante, 2015**
–	Frons with even surface; light venter; punctation on elytra surface simple; male internal protarsal claw narrow (upper branch 2/3 the width of the lower one); aedeagus Fig. 165	***Anomala piccolina* Filippini, Micó, Galante, 2015**
48	Clypeus with a straight apical side; pronotum green with ochre sides, surface homogeneous; aedeagus Fig. 151	***Anomala longisacculata* Filippini, Micó, Galante, 2015**
–	Clypeus with a sinuate apical side; pronotum with an irregular brown macula on disc, surface with wrinkles; aedeagus Fig. 141	***Anomala globulata* Filippini, Micó, Galante, 2015**
49	Pronotum and elytra covered with dense setae	**50**
–	Pronotum and elytra glabrous or with very few setae	**56**
50	Body shape rounded, with widest point at mid length of elytra; two transversal bands on elytra: a median transversal wavy band, hind band usually not reaching the posterior margin of elytra	***Anomala balzapambae* Ohaus, 1897**
–	Body shape elongated, with widest point in last third of elytra; elytra with uniform color, or with 1 or more continuous transversal bands, hind band covering apical third of elytra; when bands are defined by maculae, they are arranged in 3 rows	**51**
51	Elytra uniformly light brown or with one transversal dark band, apical third of elytra light colored; size approximately 11 mm	***Anomala flavacoma* Filippini, Micó, Galante, 2013**
–	Elytra uniformly dark brown or with two bands, apical third of elytra always dark; size varies	**52**
52	Body length > 11.0 mm	**53**
–	Body length < 10.1 mm	**54**
53	Elytra uniformly dark or with a lighter posthumeral band (and rarely, a second light band on disc); male protibia upper tooth short (less than 1/4 of total length) and almost straight; parameres with wide apex and strongly sinuate ventral margin (Fig. 170)	***Anomala pseudoeucoma* Filippini, Micó, Galante, 2013**
–	Two darker transversal bands visible on elytra; male protibia upper tooth long (greater than 1/4 of total length) and oblique; parameres with short rounded apex and slightly sinuate ventral margin (Fig. 135)	***Anomala eucoma* Bates, 1888**
54	Pronotum with irregular surface due to small depressions at sides of median sulcus; parameres wide, with blunt and wide apex, length of parameres 3/4 of tectum length, basal ventral margin longer than dorsal joint of parameres	***Anomala megaparamera* Filippini, Micó, Galante, 2013**
–	Pronotum with uniform surface, sometimes a median sulcus is present; parameres slender, with defined narrow apex, length of parameres not reaching 3/4 of tectum length, basal ventral margin as long as dorsal joint of parameres	**55**
55	Ventral margin of parameres slightly sinuous, parameres long, more than half the length of the tectum (Fig. 163)	***Anomala parvaeucoma* Filippini, Micó, Galante, 2015**
–	Ventral margin of parameres straight, parameres short, less than half the length of the tectum (Fig. 158)	***Anomala moroni* Filippini, Micó, Galante, 2015**
56	Both pronotum and elytra ochre	***Anomala megalia* Bates, 1888**
–	Either pronotum, elytra or both of darker color	**57**
57	Elytra with homogeneous color	**58**
–	Elytra with pattern of maculae or bands	**75**
58	Dark colored elytra	**59**
–	Light colored elytra	**65**
59	Small size, body length < 9.5 mm	**60**
–	Medium and large size, body length > 12.0 mm	**62**
60	Elytra regularly striated	***Anomala subaenea* (Nonfried, 1893)**
–	Elytra with rows of punctures	**61**
61	Elytra metallic green or coppery; large head (about 2/3 of pronotum width); elytra with rows of coalescing punctures	***Anomala hoppi* Ohaus, 1928**
–	Elytra brown; small head (about 1/2 of pronotum width); elytra with shallow isolated punctures	***Anomala cinaedias* nomen novum**
62	Elytra with costae defined by regular rows of punctures, and interstices with rows of punctures	***Anomala ferrea* Filippini, Micó, Galante, 2014**
–	Elytra with costae not defined, irregular surface due to coalescing punctures	**63**
63	Absence of metallic luster	***Anomala semilla* Filippini, Micó, Galante, 2014**
–	Presence of metallic luster	**64**
64	Large size (body length > 17 mm); oblong shape; clypeus with sinuate anterior margin	***Anomala obovata* Ohaus, 1933**
–	Medium size (body length < 14 mm); oval shaped; clypeus with straight anterior margin	***Anomala cupreovariolosa* Filippini, Micó, Galante, 2014**
65	Pronotum of dark homogeneous color	**66**
–	Pronotum with light colored sides	**68**
66	Pronotum black	***Anomala nigroflava* Filippini, Micó, Galante, 2014**
–	Pronotum dark brown	**67**
67	Pygidium and abdominal sternites ochre; body length > 16 mm; aedeagus Fig. 161	***Anomala ochrogastra* Bates, 1888**
–	Pygidium and abdominal sternites brown; body length < 14 mm; aedeagus Fig. 162	***Anomala ochroptera* Bates, 1888**
68	Pronotum and wide elytral suture green	**69**
–	Pronotum and narrow elytral suture brown	**70**
69	Pronotum with one green macula	***Anomala arara* Ohaus, 1897**
–	Pronotum with two maculae	***Anomala arthuri* Filippini, Micó, Galante, 2015**
70	Elytra regularly striated; size < 7.5 mm	***Anomala subaenea* (Nonfried, 1893)**
–	Elytra with primary costae and punctured interstices; size > 8.5 mm	**71**
71	Pronotum with two small dark maculae, not reaching half the length of pronotum and narrower than 1/4 of pronotum width; aedeagus Fig. 146	***Anomala jansoni* Ohaus, 1897**
–	Pronotum with one or two maculae, larger than 1/2 the length and 1/4 the width of pronotum; different aedeagus	**72**
72	Pronotum with one pentagonal central macula not reaching basal margin; aedeagus Fig. 117	***Anomala antica* Ohaus, 1897**
–	Pronotum with large irregular macula, reaching basal margin at least at sides; different aedeagus	**73**
73	Body length >14.4 mm; aedeagus Fig. 134	***Anomala estrella* Filippini, Galante, Micó, 2015**
–	Body length < 14.0 mm; different aedeagus	**74**
74	Body length > 12 mm; pronotum usually with median ochre line; males with dark brown abdominal sternites; aedeagus Fig. 133	***Anomala divisa* Filippini, Galante, Micó, 2015**
–	Body length < 10 mm; pronotum without median ochre line; males with ochre abdominal sternites; aedeagus Fig. 173	***Anomala ruatana* Bates, 1888**
75	Elytral pattern consisting of maculae or lines arranged in at least one median transversal band	**76**
–	Other elytral pattern, not forming transversal bands	**88**
76	Elytra pattern consisting of one median transversal band	**77**
–	Elytra pattern consisting of two or more transversal bands, or presence of maculae at base and apex of elytra	**80**
77	Small size (body length < 8.5 mm); elytral transversal band simple, elytral base color ochre	***Anomala unilineata* Filippini, Galante, Micó, 2015**
–	Medium size (body length > 10.5 mm); elytral transversal band crossed by vertical lines, elytral base color ochre or orangish yellow	**78**
78	Pronotum with shallow sparse punctures, surface appearing polished to the naked eye; aedeagus Fig. 168	***Anomala popayana* Ohaus, 1897**
–	Pronotum with deep dense punctures, visible to naked eye; aedeagus different	**79**
79	Elytral transversal row narrow (less than 1/8 of elytra length), with long vertical lines; aedeagus Fig. 127	***Anomala clathrata* Ohaus, 1930**
–	Elytral transversal row wide (about 1/4 of elytra length), with faint vertical lines; aedeagus Fig. 193	***Anomala zumbadoi* Filippini, Micó, Galante, 2014**
80	Medium size (body length 10–12 mm, body width 6–7 mm)	**81**
–	Small size (body length 6–9 mm, body width < 5 mm)	**82**
81	Macula on pronotum not reaching basal margin; elytra without maculae at sides of scutellum; aedeagus Fig. 191	***Anomala volsellata* Filippini, Micó, Galante, 2014**
–	Macula on pronotum reaching basal margin; elytra with maculae at sides of scutellum; aedeagus Fig. 176	***Anomala solisi* Filippini, Micó, Galante, 2014**
82	Macula on pronotal disc irregular, consisting of a longitudinal median bar with two 3-shaped maculae at sides; elytra light brown with ochre short longitudinal lines (Fig. 30)	***Anomala histrionella* Bates, 1888**
–	Macula on pronotal disc large or pronotum entirely dark; elytra ochre and dark brown	**83**
83	Pronotum of homogeneous dark color	**84**
–	Pronotum with ochre sides	**86**
84	Body shape rounded; apical third of elytra dark in color; head width less than half the basal width of pronotum	***Anomala eulissa* Bates, 1888**
–	Body shape elongated; apical third of elytra light colored with maculae; head width more than half the basal width of pronotum	**85**
85	Elytra regularly striated; pronotum long (ratio width/length < 1.3), with coalescing coarse punctures	***Anomala strigodermoides* Filippini, Galante, Micó, 2015**
–	Elytra with punctured interstices; pronotum short (ratio width/length > 1.6) with isolated fine punctures	***Anomala calligrapha* Bates, 1888**
86	Body length < 6.0 mm; apical portion of elytra mainly dark; aedeagus Fig. 124	***Anomala chiriquina* Bates, 1888**
–	Body length > 7.5 mm; apical portion of elytra light colored; aedeagus different	**87**
87	Pronotum metallic green with narrow ochre sides; elytra with two wavy bands, usually complete	***Anomala undulata* Melsheimer, 1844**
–	Pronotum brown with wide ochre sides (metallic luster present on some specimens); elytra with bands usually composed of isolated maculae	***Anomala discoidalis* Bates, 1888**
88	Elytral pattern made up of pigmented punctures on striae	**89**
–	Elytral pattern not linked to punctures	**92**
89	Body length ≥ 15 mm, body width ≥ 9 mm	***Anomala valida* Burmeister, 1844**
–	Body length ≤ 11 mm, body width ≤ 6.5 mm	**90**
90	Body length < 8.5 mm; first elytral costa defined by a sulcus	***Anomala ludoviciana* Schaeffer, 1906**
–	Body length >10.5 mm; first elytral costa defined by scattered punctures	**91**
91	First interstice on elytra with 4–5 irregular rows of punctures; elytral suture not pigmented; pygidium covered with dense short setae; males with first tooth of protibia longer than width of protibia	***Anomala foraminosa* Bates, 1888**
–	First interstice on elytra with 2–3 irregular rows of punctures; elytral suture dark brown; pygidial disc glabrous; males with first tooth of protibia shorter than width of protibia	***Anomala robiginosa* Filippini, Galante, Micó, 2015**
92	Elytra metallic green	**93**
–	Elytra black and ochre	**94**
93	Elytra with branched yellow lines at apex; pronotum entirely metallic green	***Anomala aglaos* Filippini, Galante, Micó, 2015**
–	Elytra with ochre apex; pronotum with ochre sides	***Anomala semicincta* Bates, 1888**
94	Elytra black with ochre base	***Anomala mersa* Filippini, Galante, Micó, 2015**
–	Elytra ochre with dark maculae	**95**
95	Pronotum with two dark maculae; elytra with black maculae on humeral calli	***Anomala quiche* Ohaus, 1897**
–	Pronotum entirely black; elytra with irregular black maculae mainly on sides	***Anomala limon* nomen novum**
96	Pronotum with basal bead, complete or obliterated at the middle	**97**
–	Pronotum without basal bead, margin smooth	**100**
97	Elytra regularly sulcated, homogeneously dark colored	**98**
–	Elytra punctate; pronotum with brown-reddish color and lighter colored elytra	**99**
98	Dark green color with bronze luster; elytra surface with irregular aspect due to presence of secondary rows of punctures on costae	***Callistethus nicoya* (Ohaus, 1928)**
–	Bluish black color, no metallic luster; elytra surface with smooth costae	***Callistethus sulcans* (Bates, 1888)**
99	Elytra nearly smooth, homogeneously orange-reddish in color	***Anomala praecellens* Bates, 1888**
–	Elytra with rows of coalescing sparse punctures, ochre in color	***Anomala cupricollis* Chevrolat, 1834**
100	Elytra with pattern of regular maculae and stripes in yellowish white and red or black	***Callistethus chrysomelinus* (Bates, 1888)**
–	Elytra of homogeneous color, green or brown	**101**
101	Stout body, wider at 2/3 elytra length; body surface convex; interocular space narrow (less than 3.5 times the width of eye); elytra with defined costae and punctate interstices	**102**
–	Body rhomboidal shaped, pronotum long and with anterior margin narrow, end of body narrowing steadily from half of elytra length; body surface flattened; wide interocular space (more than 3.8 times the width of eye); elytra regularly striated or nearly smooth	**110**
102	Elytra metallic green with brown hues and shallow coalescing punctures	***Callistethus yalizo* Filippini, Galante, Micó, 2015**
–	Elytra brown, metallic luster may be present, costae and punctures well defined	**103**
103	Elytra light brown, lighter in color than pronotum	**104**
–	Elytra dark brown, similar in tone to pronotum	**106**
104	Elytra with bronze luster; first interstice wide with dense punctures; pronotum glabrous	***Callistethus lativittis* Filippini, Galante, Micó, 2015**
–	Elytra without metallic luster; first interstice narrow with 1–2 rows of punctures; pronotum covered with setae	**105**
105	Mesosternal process pointed in lateral view, large and slightly tapering at apex in ventral view; parameres with squared apex (Fig. 212)	***Callistethus parapulcher* Filippini, Galante, Micó, 2015**
–	Mesosternal process blunt in lateral view, tapering strongly just above base in ventral view; parameres with acute apex (Fig. 215)	***Callistethus schneideri* (Frey, 1968)**
106	Pronotum with homogeneous color, no ochre margins	***Callistethus chontalensis* (Bates, 1888)**
–	Pronotum with ochre margins	**107**
107	Elytral surface with protruding costae, interstices flat with dense irregular punctures	***Callistethus valdecostatus* (Bates, 1888)**
–	Costae not protruding in relation to rest of elytral surface, interstices with rows of punctures	**108**
108	Mesosternal process long, slightly protruding beyond mesocoxae; first interstice of elytra with four rows of punctures, other interstices with irregular rows of punctures, flat	***Callistethus fuscorubens* Filippini, Galante, Micó, 2015**
–	Mesosternal process short, not protruding beyond mesocoxae; first interstice of elytra with three rows of punctures, other interstices with regular rows of punctures forming sulcate striae	**109**
109	Secondary small sparse punctures on the whole elytra surface; aedeagus Fig. 203; endophallus Fig. 313	***Callistethus granulipygus* (Bates, 1888)**
–	No secondary punctures on elytra, background surface smooth; aedeagus Fig. 220; endophallus Fig. 330	***Callistethus vanpatteni* (Bates, 1888)**
110	Elytra clearly striated, of variable color	**111**
–	Elytra nearly smooth with very shallow rows of punctures, black color	***Callistethus carbo* Filippini, Galante, Micó, 2015**
111	Body length > 9.5 mm; pygidium ochre with green sides; head small in relation to pronotum (head width < 0.45 pronotum width)	***Callistethus macroxantholeus* Filippini, Galante, Micó, 2015**
–	Body length < 8.6 mm; pygidium entirely yellow; head large in relation to pronotum (head width > 0.55 of pronotum width)	***Callistethus microxantholeus* Filippini, Galante, Micó, 2015**
112	Elytra surface with regular and sulcate striae, normally 14 in number	**113**
–	Elytra surface nearly smooth, or with intercostal spaces with shallow irregular punctures, never forming sulcate striae	**118**
113	Pronotum with irregular dark macula; clypeus rectangular; elytra with transversal band, yellow, without metallic luster	***Callistethus ruteloides* Filippini, Galante, Micó, 2015**
–	Pronotal disc uniform in color; clypeus subtrapezoidal; elytra with uniform color and presence of metallic luster	**114**
114	Elytra of same color as pronotum; body length < 16.0 mm	**115**
–	Elytra of lighter color than pronotum; body length > 16.0 mm	***Callistethus specularis* (Bates, 1888)**
115	Elytra and pronotum yellow; aedeagus Fig. 209	***Callistethus mimeloides* (Ohaus, 1902)**
–	Elytra and pronotum green; different aedeagus	**116**
116	Body length > 14.0 mm; ochre margins of pronotum concealed by metallic green luster; bright light green color with reddish hues	***Callistethus calonotus* (Bates, 1888)**
–	Body length < 14.0 mm; ochre margins evident at wide end of pronotum; dark to brownish metallic green color	**117**
117	Apex of parameres wide and straight in lateral view (Fig. 213)	***Callistethus pseudocollaris* Filippini, Galante, Micó, 2015**
–	Apex of parameres narrow and bending upwards in lateral view (Fig. 210)	***Callistethus multiplicatus* Filippini, Galante, Micó, 2015**
118	Elytra of same color as pronotum, green or purple blackish	**119**
–	Elytra of lighter color than pronotum, yellowish or light green	**120**
119	Elytral surface completely smooth; ventral side brownish, without metallic luster	***Callistethus chlorotoides* (Bates, 1888)**
–	Elytral surface with very shallow punctures, costae visible; ventral side with green metallic luster	***Callistethus levigatus* Filippini, Galante, Micó, 2015**
120	Body length > 20 mm	**121**
–	Body length < 17 mm	**122**
121	Costae on elytra defined by sulcated rows of punctures; aedeagus Fig. 199	***Callistethus chrysanthe* (Bates, 1888)**
–	Costae poorly defined, not sulcated; aedeagus Fig. 204	***Callistethus jordani* (Ohaus, 1902)**
122	Elytral surface irregular with small wrinkles, punctation not clearly visible to the naked eye; body length > 16.5 mm	***Callistethus stannibractea* Filippini, Galante, Micó, 2015**
–	Elytral surface with regular, clearly visible costae and punctate interstices; body length < 16 mm	**123**
123	Body length 12–13 mm; deep punctures on elytra; ventral side ochre reddish in color, or only partially with green luster (not on abdominal sternites or legs); flat sixth spiracle	***Callistethus xiphostethus* (Bates, 1888)**
–	Body length 14–15 mm; shallow punctures on elytra; ventral side completely dark metallic green; tuberculiform sixth spiracle	***Callistethus flavodorsalis* Filippini, Galante, Micó, 2015**

### Clave taxonómica para especies de la tribu Anomalini de Costa Rica

Algunas partes han sido modificadas a partir de: Jameson et al. 2003, Filippini et al. 2013, Filippini et al. 2015a, d.

**Table:** 

1	Mesoepímero visible anteriormente a la base del élitro en vista dorsal (Fig. 338B); forma del cuerpo alargada	**2**
–	Mesoepímero oculto por la base de los élitros o ligeramente expuesto (Fig. 338A); forma del cuerpo ovalada o alargada	**3**
2	Región intercoxal mesoesternal similar en ancho a la base del mesofémur; mesoepímero subrectangular, bien expuesto; clípeo del macho poco doblado al ápice; superficie dorsal de los élitros plana; pronoto más estrecho que la base de los élitros (exceptuado *Strigoderma orbicularis*)	***STRIGODERMA* Burmeister, 1844**...**7**
–	Región intercoxal mesoesternal ancha menos de 1/4 de la base del mesofémur; mesoepímero subtriangular, parcialmente expuesto; clípeo del macho ampliamente doblado al ápice; superficie dorsal de los élitros redondeada, pronoto tan ancho como la base de los élitros	***Epectinaspis costaricensis* Ramírez-Ponce & Curoe, 2014**
3	Sutura frontoclipeal incompleta; lados del clípeo elevados en la base del canto; machos con el disco del pronoto con una depresión; hembras con el margen apical del pronoto con una muesca en la mitad	***ANOMALORHINA* Jameson, Paucar-Cabrera, Solís, 2003**...**17**
–	Sutura frontoclipeal completa; lados del clípeo ligeramente elevados o planos en la base del canto; machos con el disco del pronoto uniformemente convexo; hembras con el margen apical del pronoto liso	**4**
4	Espacio entre las mesocoxas plano o ligeramente convexo que no se prolonga más allá de los mesotrocanteres; pronoto con margen basal completo o interrumpido en el medio	***ANOMALA* Samouelle, 1819**...**18**
–	Presencia de un proceso mesoesternal prolongado más allá de los mesotrocanteres; pronoto con el margen basal presente o liso	**5**
5	Parámeros perpendiculares a la falobase; quinto metatarso similar en longitud a los metatarsos 1-4 unidos, excluyendo las uñas; pequeño tamaño	***Moroniella nitidula* (Blanchard, 1851)**
–	Parámeros dispuestos en línea con la falobase; quinto metatarso más corto que los metatarsos 1-4 unidos, excluyendo las uñas	**6**
6	Proceso mesoesternal corto, no sobrepasando (o sobrepasando muy levemente) la mesocoxa; ápice del proceso romo en vista lateral	***ANOMALA* Samouelle, 1819** and ***CALLISTETHUS* Blanchard, 1851**...**96**
–	Proceso mesoesternal largo, sobrepasando la mesocoxa más de la mitad de la anchura de la misma, ápice libre en vista lateral	***CALLISTETHUS* Blanchard, 1851**...**112**
7	Pronoto con superficie irregular, granulada o con impresiones	**8**
–	Pronoto liso	**10**
8	Pronoto con superficie granulada	***Strigoderma nodulosa* Ohaus, 1902**
–	Pronoto con impresiones	**9**
9	Longitud 11–13 mm; pronoto homogéneamente negro o cobrizo; forma alargada (élitros más de 2 veces más largos que anchos)	***Strigoderma sulcipennis* Burmeister, 1844**
–	Longitud 7–9 mm; pronoto verde o marrón rojizo con lados amarillentos; forma redondeada (élitros menos de 2 veces más largos que anchos)	***Strigoderma castor* (Newman, 1838)**
10	Élitros lisos; vientre con colores metálicos brillantes	***Strigoderma auriventris* Bates, 1888**
–	Élitros estriados; vientre sin colores metálicos	**11**
11	Pronoto de color verde metálico	**12**
–	Pronoto marrón o negro, sin reflejos metálicos	**14**
12	Élitros negros con un círculo amarillo cerca de la base	***Strigoderma rutelina* Bates, 1888**
–	Élitros con color uniforme	**13**
13	Pronoto con una mancha central o color uniforme, élitros negros o marrones	***Strigoderma biolleyi* Ohaus, 1908**
–	Pronoto con dos bandas claras, élitros marrones	***Strigoderma micans* Nonfried, 1893**
14	Élitros marrones	***Strigoderma vestita* Burmeister, 1844**
–	Élitros negros o marrones con manchas negras	**15**
15	Longitud 11–12 mm	***Strigoderma angulicollis* Ohaus, 1915**
–	Longitud <8 mm	**16**
16	Forma redondeada; pronoto fuertemente convexo, tan ancho como la base de los élitros; longitud 7–8 mm	***Strigoderma orbicularis* Burmeister, 1855**
–	Forma alargada; pronoto ligeramente convexo, más estrecho que la base de los élitros; longitud 5–7 mm	***Strigoderma marginata* (Olivier, 1789)**
17	Cabeza y pronoto rojizos, élitros negros o marrones rojizos; macho con dos tubérculos en la base de la frente, clípeo con ápice agudo en vista frontal; hembras con primera interestría de los élitros dos veces más ancho que la primera estría, disco frontal ligeramente cóncavo	***Anomalorhina turrialbana* (Ohaus, 1928)**
–	Cabeza, pronoto y élitros marrones; macho sin tubérculos en la frente, clípeo con ápice cuadrado en vista frontal; hembras con primera interestría de los élitros tan ancho como la primera estría, disco frontal ligeramente convexo	***Anomalorhina osaensis* Jameson, Paucar-Cabrera, Solís, 2003**
18	Protibia con 3 dientes	**19**
–	Protibia con 2 dientes	**49**
19	Élitros de color homogéneo	**20**
–	Élitros con manchas oscuras	**22**
20	Color del cuerpo mayoritariamente marrón oscuro	***Anomala coffea* Filippini, Galante, Micó, 2015**
–	Pronoto marrón oscuro o verde, élitros color ocre	**21**
21	Pronoto de color homogéneo, verde o marrón; longitud corporal 11.0–13.0 mm; edeago en Fig. 183	***Anomala testaceipennis* Blanchard, 1851**
–	Pronoto marrón oscuro con lados amarillentos; longitud 8.5–9.5 mm; edeago en Fig. 190	***Anomala veraecrucis* Bates, 1888**
22	Cabeza y pronoto negros uniformes, élitros ocre con grandes manchas irregulares negras que se desarrollan longitudinalmente (Fig. 53)	***Anomala pincelada* Filippini, Galante, Micó, 2015**
–	Cabeza y pronoto verdes metálico o marrones, pronoto normalmente con lados amarillentos, élitro con manchas oscuras pequeñas en hileras transversales o pequeñas manchitas en toda la superficie	**23**
23	Patrón de los élitros constituido por una mancha central o una hilera transversal media	**24**
–	Patrón de los élitros constituido por varias hileras de manchas o manchitas dispersas	**25**
24	Pronoto mayoritariamente marrón oscuro, con sutiles márgenes amarillos; longitud 10–12 mm; edeago en Fig. 156	***Anomala mesosticta* Filippini, Galante, Micó, 2015**
–	Pronoto con una mancha oscura pentagonal central ocupando menos de la mitad de la anchura del pronoto; longitud 8–10 mm; edeago en Fig. 131	***Anomala cyclops* Filippini, Galante, Micó, 2015**
25	Pronoto con impresiones profundas	***Anomala inbio* (Ramírez-Ponce, Bitar, Curoe, 2014)**
–	Pronoto con superficie homogéneamente convexa	**26**
26	Presencia de setas en pronoto y élitros	**27**
–	Pronoto y élitros glabros, el pronoto puede tener unas pocas setas	**29**
27	Longitud 10.0–11.5 mm; pronoto con puntuación densa; élitros con 2 hileras regulares de manchas; edeago en Fig. 148	***Anomala leopardina* Filippini, Micó, Galante, 2015**
–	Longitud 12.0–14.0 mm; pronoto con puntuación escasa y gruesa; élitros cubiertos irregularmente por manchitas; edeago diferente	**28**
28	Color claro; pronoto con márgenes muy sinuados; edeago en Fig. 126	***Anomala clarivillosa* Filippini, Micó, Galante, 2015**
–	Color oscuro; pronoto con márgenes ligeramente sinuados; edeago en Fig. 121	***Anomala atrivillosa* Filippini, Micó, Galante, 2015**
29	Márgenes laterales del pronoto sinuados	**30**
–	Márgenes laterales del pronoto convexos o formando un ángulo	**37**
30	Lados del pronoto profundamente sinuados (cruzando una línea imaginaria que une los ángulos apicales y basales), pronoto casi completamente oscuro; edeago en Fig. 167	***Anomala polygona* Bates, 1888**
–	Lados del pronoto poco sinuados (no cruzan una línea imaginaria que une los ángulos apicales y basales); pronoto con una mancha irregular en el disco o lados amarillentos; edeago diferente	**31**
31	Pronoto oscuro con lados amarillentos; élitros con manchas regulares; edeago en Fig. 142	***Anomala hiata* Filippini, Micó, Galante, 2015**
–	Pronoto con una mancha irregular en el disco; élitros con numerosas manchitas; edeago diferente	**32**
32	Pronoto con una mancha sutil y sinuosa; élitros con escasas manchitas; longitud < 11.5 mm; parámeros largos con ápice agudo y ángulo ventral saliente en vista lateral (Fig. 157)	***Anomala m-fuscum* Filippini, Micó, Galante, 2015**
–	Pronoto con manchas más grandes; élitros con numerosas manchitas; longitud 10.2–13.4 mm, edeagos diferentes	**33**
33	Placa ventral con ángulos apicales alargados, parámeros más cortos que la mitad de la longitud del tecto	***Anomala vulcanicola* Ohaus, 1897**
–	Placa ventral con ápice curvo en vista ventral, parámeros más largos que la mitad de la longitud del tecto	**34**
34	Placa ventral con porción apical curva en vista lateral	**35**
–	Placa ventral con ápice recto	**36**
35	Parámeros cortos con ápice romo en vista lateral (Fig. 120)	***Anomala aspersa* Filippini, Micó, Galante, 2015**
–	Parámeros largos con ápice agudo en vista lateral (Fig. 184)	***Anomala trapezifera* Bates, 1888**
36	Parámeros largos y estrechos, margen dorsal curvo, ángulo ventral obtuso (Fig. 180)	***Anomala subridens* Filippini, Micó, Galante, 2015**
–	Parámeros cortos y anchos, margen dorsal sinuado, ángulo ventral apuntando hacia atrás en vista lateral (Fig. 189)	***Anomala vallisneria* Filippini, Micó, Galante, 2015**
37	Élitros de color claro con manchitas esparcidas; longitud > 14.1 mm	**38**
–	Élitros de color claro y longitud < 13.5 mm; o élitro oscuro y longitud > 14.1 mm	**40**
38	Presencia de una protuberancia en el clípeo, edeago en Fig. 185	***Anomala tuberculata* Filippini, Micó, Galante, 2015**
–	Superficie del clípeo plana, edeago diferente	**39**
39	Pronoto con una mancha irregular en el disco; élitros con manchas grandes; edeago en Fig. 137	***Anomala eusticta* Filippini, Micó, Galante, 2015**
–	Pronoto completamente oscuro; élitros con manchitas pequeñas; edeago en Fig. 115	***Anomala aereiventris* Filippini, Micó, Galante, 2015**
40	Superficie del pronoto lisa	**41**
–	Superficie del pronoto con puntuación evidente	**42**
41	Pronoto completamente oscuro; partes inferiores del cuerpo homogéneamente marrón-bronce, metálicas; tercer diente de la protibia desarrollado débilmente; longitud > 13.2 mm; edeago en Fig. 115.	***Anomala aereiventris* Filippini, Micó, Galante, 2015**
–	Pronoto con lados amarillentos; partes inferiores del cuerpo marrones con zonas amarillas; tercer diente de la protibia bien desarrollado; longitud < 13.0 mm; edeago as in Fig. 149	***Anomala levicollis* Filippini, Micó, Galante, 2015**
42	Macho con ojos grandes (proporción espacio interocular/diámetro del ojo < 1.8); tamaño mediano	**43**
–	Macho con ojos pequeños (proporción espacio interocular/diámetro del ojo > 2.2); tamaño medio y grande	**44**
43	Pronoto con puntuación gruesa, mitad basal de los márgenes laterales paralelos; élitros con manchitas pequeñas esparcidas irregularmente; edeago en Fig. 164	***Anomala perspicax* Filippini, Micó, Galante, 2015**
–	Pronoto con puntuación fina, mitad basal de los márgenes laterales oblicuos respecto a la base; élitros con manchas grandes organizadas en 3 hileras transversales; edeago en Fig. 177	***Anomala stillaticia* Filippini, Micó, Galante, 2015**
44	Longitud > 13.0 mm, anchura >7 mm; élitros casi completamente oscuros	**45**
–	Longitud < 12.6 mm, anchura < 6.8 mm; élitros normalmente con abundantes manchitas claras	**46**
45	Pronoto verde; élitros con manchas amarillas alargadas; pigidio de color claro; edeago en Fig. 182	***Anomala tenoriensis* Filippini, Micó, Galante, 2015**
–	Pronoto marrón; élitros con numerosas manchitas pequeñas amarillas; pigidio con una grande mancha oscura; edeago en Fig. 181	***Anomala subusta* Filippini, Micó, Galante, 2015**
46	Mitad basal de los lados laterales del pronoto paralelos; longitud <=10.0 mm, anchura < 5.2 mm	**47**
–	Mitad basal de los lados laterales del pronoto oblicuos respecto a la base; longitud generalmente > 11.0 mm, anchura > 5.9 mm	**48**
47	Frente cóncava; zonas inferiores oscuras; presencia de micropuntuación en la superficie de los élitros; uña protarsal interna del macho ancha (rama superior 1/4 de ancha de la inferior); edeago en Fig. 147	***Anomala latifalculata* Filippini, Micó, Galante, 2015**
–	Frente con superficie plana; zonas inferiores claras; puntuación de los élitros simple; uña protarsal interna del macho estrecha (rama superior 2/3 de ancha de la inferior); edeago en Fig. 165	***Anomala piccolina* Filippini, Micó, Galante, 2015**
48	Clípeo con lado apical recto; pronoto verde con lados amarillentos, con superficie homogénea; edeago en Fig. 151	***Anomala longisacculata* Filippini, Micó, Galante, 2015**
–	Clípeo con ápice sinuado; pronoto con una mancha marrón irregular en el disco, con superficie irregular; edeago en Fig. 141	***Anomala globulata* Filippini, Micó, Galante, 2015**
49	Pronoto y élitros cubiertos densamente por sedas	**50**
–	Pronoto y élitros glabros o con muy pocas sedas	**56**
50	Forma redondeada, con la máxima anchura a mitad de la longitud de los élitros; 2 bandas transversales en los élitros: una banda mediana ondulada constituida por manchas separadas y otra en el tercio apical, normalmente sin llegar a él	***Anomala balzapambae* Ohaus, 1897**
–	Forma alargada, con la máxima anchura en el último tercio de los élitros; élitros de color uniforme, o con 1 ó más bandas transversales continuas, la banda inferior cubre el tercio apical del élitro, cuando las bandas están definidas por manchas, se organizan en 3 bandas transversales	**51**
51	Élitros uniformemente marrón claro o con 1 banda transversal oscura; longitud aproximadamente 11 mm	***Anomala flavacoma* Filippini, Micó, Galante, 2013**
–	Élitros uniformemente marrón oscuros o con 2 bandas transversales, tercio apical del élitro siempre oscuro, longitud variada	**52**
52	Longitud mayor a 11 mm	**53**
–	Longitud menor a 10.1 mm	**54**
53	Élitros uniformemente oscuros o con una zona basal clara (raramente está presente una segunda banda clara en el disco); diente superior de la protibia del macho corto (menos de 1/4 de la longitud total de la protibia) y recto; edeago con ápice ancho y margen ventral fuertemente sinuado (Fig. 170)	***Anomala pseudoeucoma* Filippini, Micó, Galante, 2013**
–	Élitros con 2 bandas oscura transversales; diente superior de la protibia del macho largo (más de 1/4 de la longitud total de la protibia) y oblicuo; parámeros con ápice redondeado y margen ventral débilmente sinuado (Fig. 135)	***Anomala eucoma* Bates, 1888**
54	Pronoto con superficie irregular debida a pequeñas depresiones a los lados de un surco mediano; parámeros anchos, longitud máxima 3/4 de la longitud del tecto, con ápice romo y ancho, margen ventral basal más largo que la unión dorsal de los parámeros	***Anomala megaparamera* Filippini, Micó, Galante, 2013**
–	Pronoto con superficie uniforme, a veces está presente un surco mediano; parámeros esbeltos, con ápice definido y estrecho, longitud de los parámeros no llega a 3/4 de la longitud del tecto; margen ventral basal tan largo como la unión dorsal de los parámeros	**55**
55	Margen ventral de los parámeros ligeramente sinuada, parámeros largos, más de la mitad de la longitud del tecto (Fig. 163)	***Anomala parvaeucoma* Filippini, Micó, Galante, 2015**
–	Margen ventral de los parámeros recto, parámeros cortos, menos de la mitad de la longitud del tecto (Fig. 158)	***Anomala moroni* Filippini, Micó, Galante, 2015**
56	Pronoto y élitros amarillo claro	***Anomala megalia* Bates, 1888**
–	Pronoto o élitros o ambos de color oscuro	**57**
57	Élitros de color homogéneo	**58**
–	Élitros con manchas o bandas	**75**
58	Élitros de color oscuro	**59**
–	Élitros de color claro	**65**
59	Tamaño pequeño (longitud <9.5 mm)	**60**
–	Tamaño medio y grande (longitud >12 mm)	**62**
60	Élitros con surcos regulares	***Anomala subaenea* (Nonfried, 1893)**
–	Élitros con hileras de puntos	**61**
61	Élitros verde metálico o cobrizos; cabeza ancha (alrededor de 2/3 de la anchura del pronoto); élitros con hilera de puntos coalescentes	***Anomala hoppi* Ohaus, 1928**
–	Élitros marrones; cabeza pequeña (alrededor de 1/2 de la anchura del pronoto); élitros con puntos aislados y pocos profundos	***Anomala cinaedias* nomen novum**
62	Élitros con estrías definidas por hileras regulares de puntos, interestrias con hileras de puntos	***Anomala ferrea* Filippini, Micó, Galante, 2014**
–	Élitros con superficie irregular por coalescencia de puntos, estrías no definidas	**63**
63	Superficie sin reflejos metálicos	***Anomala semilla* Filippini, Micó, Galante, 2014**
–	Superficie con reflejos metálicos	**64**
64	Tamaño grande (longitud > 17 mm); forma oblonga; clípeo con margen anterior sinuado	***Anomala obovata* Ohaus, 1933**
–	Tamaño mediano (longitud < 14 mm); forma ovalada; clípeo con margen anterior recto	***Anomala cupreovariolosa* Filippini, Micó, Galante, 2014**
65	Pronoto uniformemente oscuro	**66**
–	Pronoto con bandas claras a los lados	**68**
66	Pronoto negro	***Anomala nigroflava* Filippini, Micó, Galante, 2014**
–	Pronoto marrón oscuro	**67**
67	Pigidio y esternitos abdominales de color ocre; longitud > 16 mm; edeago en Fig. 161	***Anomala ochrogastra* Bates, 1888**
–	Pigidio y esternitos abdominales marrones; longitud <14 mm; edeago en Fig. 162	***Anomala ochroptera* Bates, 1888**
68	Pronoto y sutura elitral ancha de color verde	**69**
–	Pronoto y sutura elitral estrecha de color marrón	**70**
69	Pronoto con 1 mancha verde	***Anomala arara* Ohaus, 1897**
–	Pronoto con 2 manchas verdes	***Anomala arthuri* Filippini, Micó, Galante, 2015**
70	Élitros estriados regularmente; longitud < 7.5 mm	***Anomala subaenea* (Nonfried, 1893)**
–	Élitros con estrias primarias definidas e interestrias punteadas; longitud > 8.5 mm	**71**
71	Pronoto con 2 manchas pequeñas, no llegando a mitad de la longitud del pronoto y más estrechas que 1/4 de la anchura del pronoto; edeago en Fig. 146	***Anomala jansoni* Ohaus, 1897**
–	Pronoto con 1 ó 2 manchas, más grandes que la mitad de la longitud y 1/4 de la anchura del pronoto, edeago diferente	**72**
72	Pronoto con una mancha pentagonal central no llegando al margen basal; edeago en Fig. 117	***Anomala antica* Ohaus, 1897**
–	Pronoto con mancha grande irregular llegando al margen basal por lo menos en los lados; edeago diferente	**73**
73	Longitud >14.4 mm; edeago en Fig. 134	***Anomala estrella* Filippini, Galante, Micó, 2015**
–	Longitud <14.0 mm; edeago diferente	**74**
74	Longitud >12 mm; pronoto normalmente con una línea mediana amarilla; machos con esternitos abdominales marrón oscuros; edeago en Fig. 133	***Anomala divisa* Filippini, Galante, Micó, 2015**
–	Longitud <10 mm; pronoto con una mancha entera; machos con esternitos abdominales amarillos, edeago en Fig. 173	***Anomala ruatana* Bates, 1888**
75	Patrón elitral constituido por manchas o líneas organizadas en por lo menos una banda transversal mediana	**76**
–	Patrón elitral diferente, que no forma bandas transversales	**88**
76	Patrón elitral constituido por una banda transversal mediana	**77**
–	Patrón elitral constituido por 2 ó más bandas transversales, o con manchas aisladas a la base y ápice del élitro	**80**
77	Tamaño pequeño (longitud < 8.5 mm); élitro con banda transversal simple, color basal del élitro ocre	***Anomala unilineata* Filippini, Galante, Micó, 2015**
–	Tamaño mediano (longitud 10.5 mm); élitro con banda transversal cruzada por líneas verticales, color basal del élitro ocre o anaranjado	**78**
78	Pronoto con puntuación poco profunda y esparcida, casi liso a simple vista; edeago en Fig. 168	***Anomala popayana* Ohaus, 1897**
–	Pronoto con puntuación profunda y densa; edeago diferente	**79**
79	Banda transversal del élitro sutil (menos que 1/8 de la longitud del élitro), con líneas verticales largas; edeago en Fig. 127	***Anomala clathrata* Ohaus, 1930**
–	Banda transversal del élitro ancha (cerca de 1/4 de la longitud del élitro), con líneas verticales pobremente definidas; edeago en Fig. 193	***Anomala zumbadoi* Filippini, Micó, Galante, 2014**
80	Tamaño mediano (longitud 10–12 mm, anchura 6–7 mm)	**81**
–	Tamaño pequeño (longitud 6–9 mm, anchura <5 mm)	**82**
81	Mancha en el pronoto no llegando al margen basal; élitros sin manchas a los lados del escutelo; edeago en Fig. 191	***Anomala volsellata* Filippini, Micó, Galante, 2014**
–	Mancha en el pronoto llegando al margen basal; élitros con manchas a los lados del escutelo; edeago en Fig. 176	***Anomala solisi* Filippini, Micó, Galante, 2014**
82	Mancha del disco pronotal irregular, constituida por una banda longitudinal mediana con dos manchas en forma de 3 a los lados; élitros marrón claro con cortas líneas amarillas longitudinales (Fig. 30)	***Anomala histrionella* Bates, 1888**
–	Mancha del pronoto extendida (a menudo ocupando casi completamente el pronoto); élitros ocre y marrón oscuro	**83**
83	Pronoto de color oscuro	**84**
–	Pronoto con bandas laterales amarillas	**86**
84	Forma redondeada; tercio apical del élitro de color oscuro; ancho de la cabeza menor a la mitad de la anchura basal del pronoto	***Anomala eulissa* Bates, 1888**
–	Forma alargada; tercio apical del élitro de color claro con manchas; ancho de la cabeza mayor de la mitad de la anchura basal del pronoto	**85**
85	Élitros estriados regularmente; pronoto largo (proporción ancho/largo < 1.3), con puntuación gruesa coalescente	***Anomala strigodermoides* Filippini, Galante, Micó, 2015**
–	Élitros con estrías definidas e intersticios punteados; pronoto corto (proporción ancho/largo > 1.6) con puntuación fina y poco densa	***Anomala calligrapha* Bates, 1888**
86	Longitud <6.0 mm; ápice del élitro en mayoría oscuro; edeago en Fig. 124	***Anomala chiriquina* Bates, 1888**
–	Longitud >7.5 mm; ápice del élitro de color claro; edeago diferente	**87**
87	Pronoto verde metálico con bandas amarillas estrechas en los lados; élitros con 2 bandas onduladas en general continuas	***Anomala undulata* Melsheimer, 1844**
–	Pronoto marrón con bandas amarillas anchas a los lados (con reflejos metálicos en algunos especímenes); bandas constituidas normalmente por manchas separadas	***Anomala discoidalis* Bates, 1888**
88	Patrón elitral constituido por puntos pigmentados en las estrías	**89**
–	Patrón elitral no vinculado a la puntuación	**92**
89	Longitud >= 15 mm, ancho >= 9 mm	***Anomala valida* Burmeister, 1844**
–	Longitud <= 11 mm, ancho <= 6.5 mm	**90**
90	Longitud < 8.5 mm; primera estría elitral definida por un surco	***Anomala ludoviciana* Schaeffer, 1906**
–	Longitud >10.5 mm; primera estría elitral definida por puntos aislados	**91**
91	Primer intersticio del élitro con 4–5 hileras irregulares de puntos; sutura elitral no pigmentada; pigidio cubierto por sedas densas y cortas; en machos el primer diente de la protibia es más largo que la anchura de la protibia	***Anomala foraminosa* Bates, 1888**
–	Primer intersticio del élitro con 2–3 hileras irregulares de puntos; sutura elitral marrón oscuro; disco pigidial glabro; en machos el primer diente de la protibia es más corto que la anchura de la protibia	***Anomala robiginosa* Filippini, Galante, Micó, 2015**
92	Élitros verde metálico	**93**
–	Élitros negro y ocre	**94**
93	Élitros con líneas amarillas ramificadas al ápice; pronoto completamente verde	***Anomala aglaos* Filippini, Galante, Micó, 2015**
–	Élitros con ápice amarillo; pronoto con lados amarillentos	***Anomala semicincta* Bates, 1888**
94	Élitros negros con base color ocre	***Anomala mersa* Filippini, Galante, Micó, 2015**
–	Élitros ocre con manchas oscuras	**95**
95	Pronoto con dos manchas oscuras; élitros con manchas negras en los callos	***Anomala quiche* Ohaus, 1897**
–	Pronoto enteramente negro; élitros con manchas irregulares negras concentradas en los lados	***Anomala limon* nomen novum**
96	Pronoto con reborde basal, completo o interrumpido en el medio	**97**
–	Pronoto sin reborde basal, margen liso	**100**
97	Élitros con surcos regulares; color oscuro homogéneo	**98**
–	Élitros punteados; pronoto marrón rojizo y élitros más claros	**99**
98	Color verde oscuro con reflejos bronce; superficie elitral irregular por la presencia de hileras secundarias de puntos en las estrías	***Callistethus nicoyus* (Ohaus, 1928)**
–	Color negro azulado, sin reflejos metálicos; superficie elitral con estrías lisas	***Callistethus sulcans* (Bates, 1888)**
99	Élitros casi lisos, de un color anaranjado rojizo	***Anomala praecellens* Bates, 1888**
–	Élitros con hileras de puntos coalescentes, color ocre	***Anomala cupricollis* Chevrolat, 1834**
100	Élitros con un patrón de grandes manchas regulares y bandas en rojo o negro y blanco amarillento	***Callistethus chrysomelinus* (Bates, 1888)**
–	Élitros de color homogéneo, verde o marrón	**101**
101	Forma ovalada ancha, máxima anchura a 2/3 de la longitud elitral, superficie convexa; espacio interocular estrecho (menor a 3.5 veces la anchura de los ojos); élitros con estrías definidas e intersticios punteados	**102**
–	Forma romboidal, pronoto largo y con margen anterior estrecho, ápice del cuerpo estrechándose gradualmente a partir de mitad de los élitros, superficie aplanada; espacio interocular ancho (más de 3.8 veces la anchura de los ojos); élitros estriados regularmente o casi lisos	**110**
102	Élitros verdes metálicos con sombras marrones, puntuación poco profunda y coalescente	***Callistethus yalizo* Filippini, Galante, Micó, 2015**
–	Élitros marrones, pueden presentar reflejos metálicos, estrías y puntuación bien definidas	**103**
103	Élitros marrones claros, más claros que el pronoto	**104**
–	Élitros marrón oscuro, similar en tonalidad al pronoto	**106**
104	Élitros con reflejos bronce, primer intersticio del élitro ancho y con puntuación densa; pronoto glabro	***Callistethus lativittis* Filippini, Galante, Micó, 2015**
–	Élitros sin reflejos metálicos, primer intersticio del élitro estrecho, con 1-2 hileras de puntos; pronoto cubierto con setas	**105**
105	Proceso mesoesternal agudo en vista lateral, ancho y ligeramente más estrecho al ápice en vista ventral; parámeros con ápice cuadrado (Fig. 212)	***Callistethus parapulcher* Filippini, Galante, Micó, 2015**
–	Proceso mesoesternal romo en vista lateral, estrechándose fuertemente justo arriba de la base en vista ventral; parámeros con ápice agudo (Fig. 215)	***Callistethus schneideri* (Frey, 1968)**
106	Pronoto de color homogéneo, sin lados amarillentos	***Callistethus chontalensis* (Bates, 1888)**
–	Pronoto con lados amarillentos	**107**
107	Superficie elitral con estrías salientes, intersticios planos con puntuación irregular y densa	***Callistethus valdecostatus* (Bates, 1888)**
–	Estrías no salientes respecto al resto de la superficie elitral, intersticios con hileras de puntos	**108**
108	Proceso mesoesternal largo, sobrepasando ligeramente la mesocoxa; primer intersticio elitral con 4 hileras de puntos, demás intersticios con hileras irregulares de puntos, planos	***Callistethus fuscorubens* Filippini, Galante, Micó, 2015**
–	Proceso mesoesternal corto, no sobrepasando las mesocoxas; primer intersticio con 3 hileras de puntos, demás intersticios con hileras regulares de puntos formando estrías surcadas	**109**
109	Puntuación secundaria fina y esparcida por la superficie elitral; edeago en Fig. 203; endofalo en Fig. 313	***Callistethus granulipygus* (Bates, 1888)**
–	Sin puntuación secundaria en la superficie elitral; edeago en Fig. 220; endofalo Fig. 330	***Callistethus vanpatteni* (Bates, 1888)**
110	Élitros estriados bien definidos, de color variable	**111**
–	Élitros casi lisos, con hileras de puntos muy superficiales, color negro	***Callistethus carbo* Filippini, Galante, Micó, 2015**
111	Longitud > 9.50 mm; pigidio amarillo con lados verdes; cabeza pequeña en relación al pronoto (anchura de la cabeza < 0.45 de la anchura del pronoto)	***Callistethus macroxantholeus* Filippini, Galante, Micó, 2015**
–	Longitud < 8.60 mm; pigidio amarillo; cabeza grande en relación al pronoto (anchura de la cabeza > 0.55 de la anchura del pronoto)	***Callistethus microxantholeus* Filippini, Galante, Micó, 2015**
112	Superficie elitral con estrías regulares y surcadas, normalmente 14 en número	**113**
–	Superficie elitral casi lisa, o con intersticios elitrales con puntuación irregular, que no forma estrías	**118**
113	Pronoto con mancha oscura irregular; clípeo rectangular; élitros con bandas transversales, amarillos, sin reflejos metálicos	***Callistethus ruteloides* Filippini, Galante, Micó, 2015**
–	Disco del pronoto de color uniforme; clípeo subtrapezoidal; élitros de color uniforme y con reflejos metálicos	**114**
114	Color dorsal uniforme, longitud < 16.0 mm	**115**
–	Élitros de color más claros que el pronoto, longitud > 16.0 mm	***Callistethus specularis* (Bates, 1888)**
115	Élitros y pronoto de color amarillo; edeago en Fig. 209	***Callistethus mimeloides* (Ohaus, 1902)**
–	Élitros y pronoto de color verde, edeago diferente	**116**
116	Longitud del cuerpo > 14.0 mm; pronoto con bordes laterales amarillos enmascarados por un reflejo verde metálico; color verde claro brillante con reflejos rojizos	***Callistethus calonotus* (Bates, 1888)**
–	Longitud del cuerpo < 14.0 mm; pronoto con bordes laterales amarillos anchos y bien visibles; color verde oscuro o parduzco	**117**
117	Ápice de los parámeros anchos y rectos en vista lateral (Fig. 213)	***Callistethus pseudocollaris* Filippini, Galante, Micó, 2015**
–	Ápice de los parámeros estrechos y curvado hacia arriba en vista lateral (Fig. 210)	***Callistethus multiplicatus* Filippini, Galante, Micó, 2015**
118	Élitros del mismo color que el pronoto, verde o negruzco púrpura	**119**
–	Élitros de color más claro que el pronoto, amarillento o verde claro	**120**
119	Superficie elitral lisa; superficie ventral marrón, sin reflejos metálicos	***Callistethus chlorotoides* (Bates, 1888)**
–	Superficie elitral con puntuación superficial, estrías visibles; superficie ventral con reflejos metálicos verdes	***Callistethus levigatus* Filippini, Galante, Micó, 2015**
120	Longitud > 20 mm	**121**
–	Longitud < 17 mm	**122**
121	Estrías de los élitros definidas por hileras de puntos surcadas; edeago en Fig. 199	***Callistethus chrysanthe* (Bates, 1888)**
–	Estrías poco definidas, no surcadas; edeago en Fig. 204	***Callistethus jordani* (Ohaus, 1902)**
122	Superficie elitral irregular, con pequeñas arrugas, puntuación no definida a simple vista; longitud > 16.5 mm	***Callistethus stannibractea* Filippini, Galante, Micó, 2015**
–	Superficie elitral regular, con estrías identificables e intersticios punteados; longitud < 16 mm	**123**
123	Longitud 12–13 mm; puntuación profunda en los élitros; superficie ventral amarillo rojizo, o sólo parcialmente con reflejos verdes metálicos; sexto espiráculo plano	***Callistethus xiphostethus* (Bates, 1888)**
–	Longitud 14–15 mm; puntuación superficial en los élitros; superficie ventral verde oscuro metálico; sexto espiráculo tuberculiforme	***Callistethus flavodorsalis* Filippini, Galante, Micó, 2015**

## Plates

**Figures 2–16.**
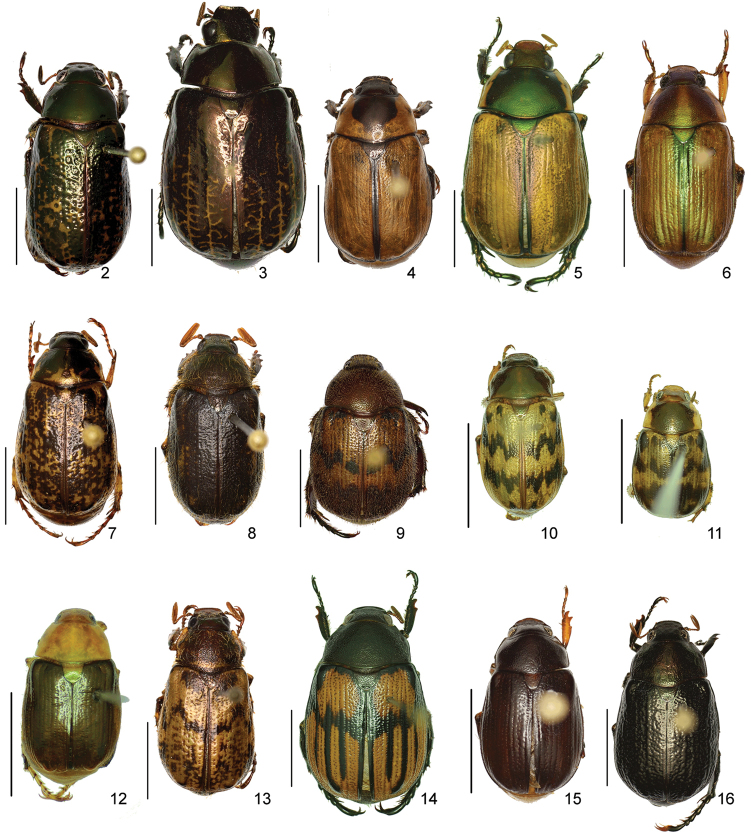
For each specimen is given the locality and province of recollection, and the collection where it is stored. Habitus. **2** *Anomala aereiventris* (Parque Nacional Tapantí, Cartago, MNCR) **3** *Anomala aglaos* (La Montura, San José, CEUA) **4** *Anomala antica* (Estación Palo Verde, Guanacaste, MNCR) **5** *Anomala arara* (Albergue Heliconias, Alajuela, CEUA) **6** *Anomala arthuri* (Estación Maritza, Guanacaste, MNCR) **7** *Anomala aspersa* (Villa Mills, Cartago, MNCR) **8** *Anomala atrivillosa* (Estación Barva, Heredia, MNCR) **9** *Anomala balzapambae* (Reserva biológica Hitoy Cerere, Limón, MNCR) **10** *Anomala calligrapha* (Estación Cabro Muco, Guanacaste, CEUA) **11** *Anomala chiriquina* (Finca Cafrosa, Puntarenas, MNCR) **12** *Anomala cinaedias* (San Luis, Puntarenas, MNCR) **13** *Anomala clarivillosa* (La Esperanza, Cartago, CEUA) **14** *Anomala clathrata* (Estación Cabro Muco, Guanacaste, CEUA) **15** *Anomala coffea* (Estación Pitilla, Guanacaste, MNCR) **16** *Anomala cupreovariolosa* (Las Cruces, Puntarenas, MNCR). Scale bars: 5 mm. Figs 6, 16 from Filippini et al. (2014); Fig. 3 from Filippini et al. (2015b); Fig. 15 from Filippini et al. (2015c); Figs 2, 7, 8, 13 from Filippini et al. (2015d).

**Figures 17–31.**
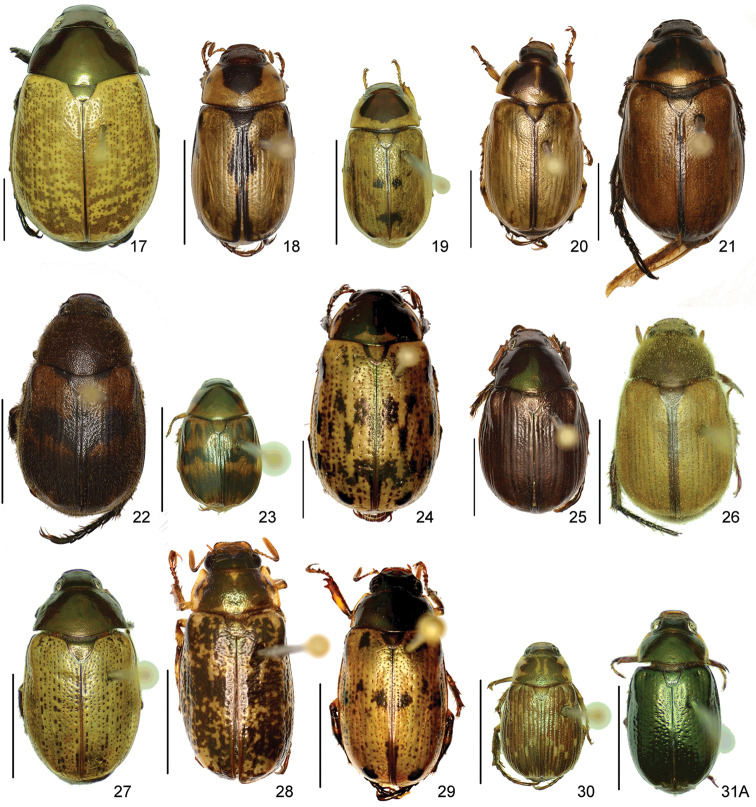
Habitus. **17** *Anomala cupricollis* (Finca San Gabriel, Alajuela, MNCR) **18** *Anomala cyclops* (Cerro El Hacha, Guanacaste, MNCR) **19** *Anomala discoidalis* (Estación Biológica Las Alturas, Puntarenas, MNCR) **20** *Anomala divisa* (Cinco esquinas de Carrizal, Alajuela, MNCR) **21** *Anomala estrella* (Estación La Casona, Puntarenas, MNCR) **22** *Anomala eucoma* (Amubri, Limón, MNCR) **23** *Anomala eulissa* (Sector Cedrales de la Rita, Limón, MNCR) **24** *Anomala eusticta* (Estación La Casona, Puntarenas, MNCR) **25** *Anomala ferrea* (Las Cruces, Puntarenas, MNCR) **26** *Anomala flavacoma* (Estación Hitoy Cerere, Limón, MNCR) **27** *Anomala foraminosa* (Estación Hitoy Cerere, Limón, MNCR) **28** *Anomala globulata* (Macizo de la Muerte, Cartago, MNCR) **29** *Anomala hiata* (Estación Pittier, Puntarenas, MNCR) **30** *Anomala histrionella* (Estación Murcielago, Guanacaste, MNCR) **31** *Anomala hoppi*, showing variable colorations (A: Río San Lorencito, Alajuela, MNCR; B: Las Cruces, Puntarenas, MNCR). Scale bars: 5 mm. Fig. 25 from Filippini et al. (2014); Fig. 21 from Filippini et al. (2015b); Figs 18, 20 from Filippini et al. (2015c); Figs 24, 28–29 from Filippini et al. (2015d).

**Figures 32–46.**
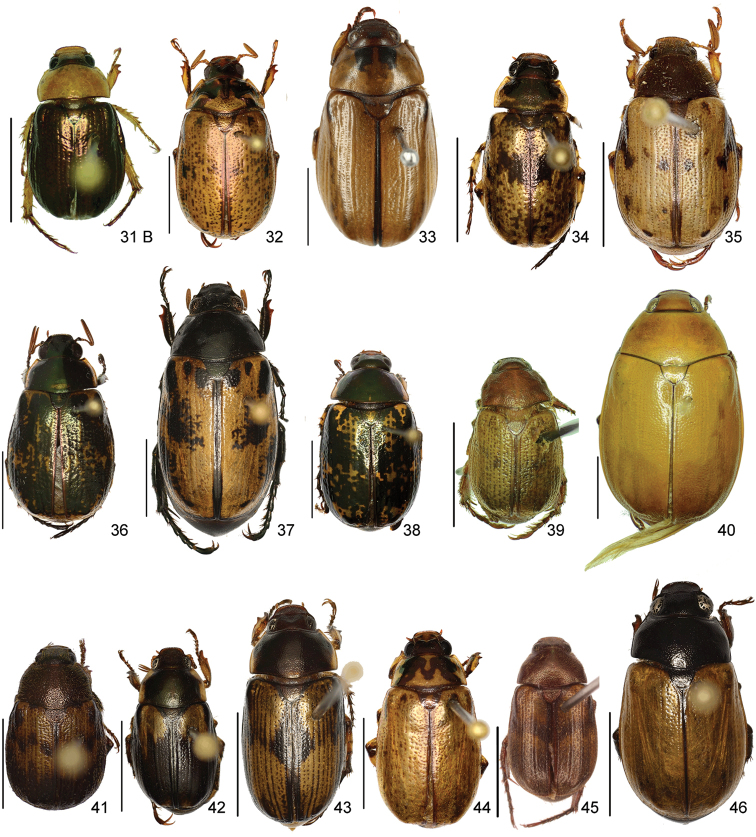
Habitus. **32** *Anomala inbio* (Volcán Tenorio, Guanacaste, CEUA) **33** *Anomala jansoni* (Monte Rotondo, Costa Rica, MNHUB) **34** *Anomala latifalculata* (Zona Protectora Cerros de la Carpintera, Cartago, MNCR) **35** *Anomala leopardina* (Finca Cafrosa, Puntarenas, MNCR) **36** *Anomala levicollis* (Cerro Montezuma, Alajuela, CEUA) **37** *Anomala limon* (Estación Hitoy Cerere, Limón, MNCR) **38** *Anomala longisacculata* (La Montura, San José, CEUA) **39** *Anomala ludoviciana* (Parque Nacional Santa Rosa, Guanacaste, MNCR) **40** *Anomala megalia* (Cerro Tortuguero, Limón, MNCR) **41** *Anomala megaparamera* (Estación Cuatro Esquinas, Limón , MNCR) **42** *Anomala mersa* (Sector Palo Verde, Guanacaste, MNCR) **43** *Anomala mesosticta* (Los Arbolitos, heredia, MNCR) **44** *Anomala m-fuscum* (Río Grande de Orosí, Cartago, MNCR) **45** *Anomala moroni* (Estación Palo Verde, Guanacaste, MNCR) **46** *Anomala nigroflava* (Río Rincon, Puntarenas, MNCR). Scale bars: 5 mm. Fig. 46 from Filippini et al. (2014); Fig. 37 from Filippini et al. (2015b); Figs 42–43 from Filippini et al. (2015c); Figs 32, 34–36, 38, 44 from Filippini et al. (2015d); Fig. 45 from Filippini et al. (2015e).

**Figures 47–62.**
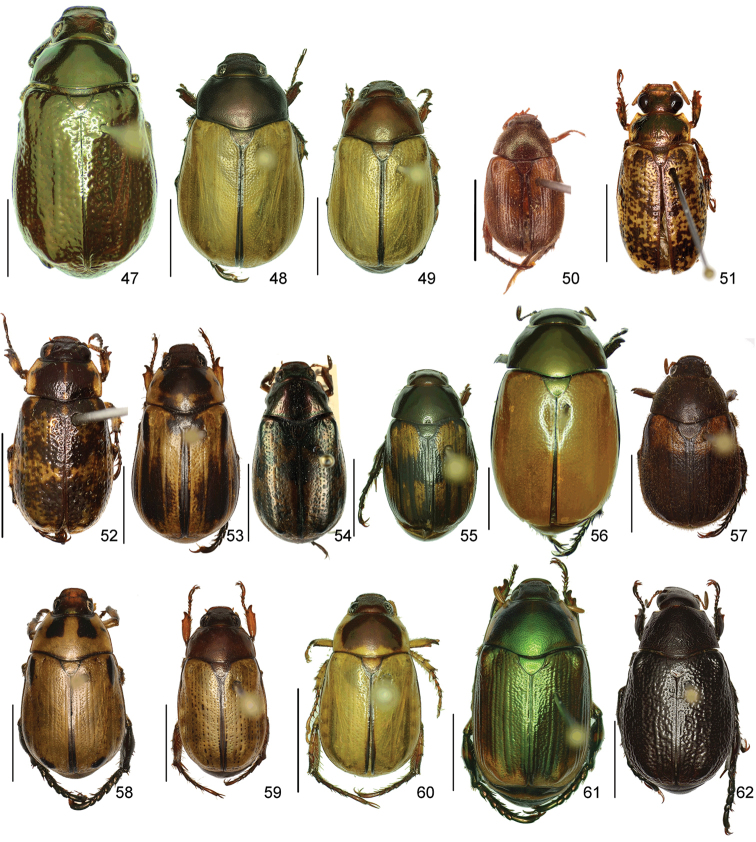
Habitus. **47** *Anomala obovata* (Cerro Chompipe, Heredia, MNCR) **48** *Anomala ochrogastra* (Estación Biológica Las Alturas, Puntarenas, MNCR) **49** *Anomala ochroptera* (La Maritza, Guanacaste, MNCR) **50** *Anomala parvaeucoma* (Estación Sirena, Puntarenas, MNCR) **51** *Anomala perspicax* (La Esperanza, Cartago CEUA) **52** *Anomala piccolina* (Estación Biológica Las Alturas, Puntarenas, MNCR) **53** *Anomala pincelada* (Finca Jenny, Guanacaste, MNCR) **54** *Anomala polygona* (Escazu, Costa Rica, MNHUB) **55** *Anomala popayana* (Reserva Biológica Hitoy Cerere, Limón, MNCR) **56** *Anomala praecellens* (Orosilito, Guanacaste, CEUA) **57** *Anomala pseudoeucoma* (Estación Hitoy Cerere, Limón, MNCR) **58** *Anomala quiche* (Estación Maritza, Guanacaste, MNCR) **59** *Anomala robiginosa* (Zarcero, Alajuela, MNCR) **60** *Anomala ruatana* (Playa Naranjo, Guanacaste, MNCR) **61** *Anomala semicincta* (Estación Cabro Muco, Guanacaste, CEUA) **62** *Anomala semilla* (Albergue Heliconias, Alajuela, CEUA). Scale bars: 5 mm. Fig. 62 from Filippini et al. (2014); Fig. 53 from Filippini et al. (2015b); Fig. 59 from Filippini et al. (2015c); Figs 51–52, 54 from Filippini et al. (2015d); Fig. 50 from Filippini et al. (2015e).

**Figures 63–76.**
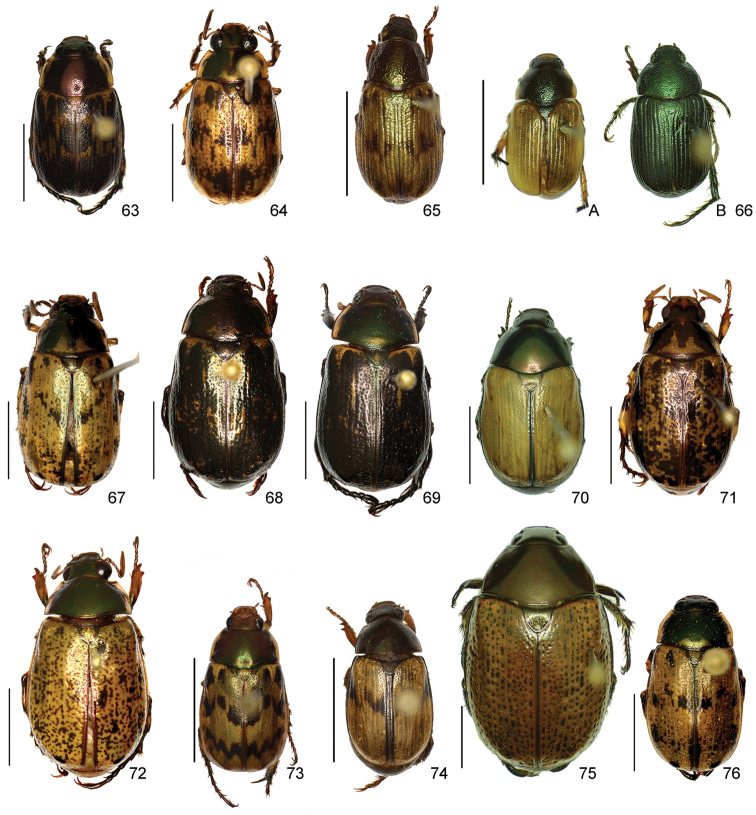
Habitus. **63** *Anomala solisi* (Amubri, Limón, MNCR) **64** *Anomala stillaticia* (La Catarata, Cartago, MNCR) **65** *Anomala strigodermoides* (holotype) **66** *Anomala subaenea*, showing variable colorations (A: Parque Nacional Santa Rosa, Guanacaste, MNCR; B: Estación Maritza, Guanacaste, MNCR) **67** *Anomala subridens* (Reserva Forestal Río Macho, Cartago, MNCR) **68** *Anomala subusta* (Estación Cacao, Guanacaste, MNCR) **69** *Anomala tenoriensis* (Parque Nacional Volcán Tenorio, Alajuela, MNCR) **70** *Anomala testaceipennis* (Cecafor, Heredia, MNCR) **71** *Anomala trapezifera* (Parque Nacional Tapantí, Cartago, CEUA) **72** *Anomala tuberculata* (Isla Bonita, Alajuela, CEUA) **73** *Anomala undulata* (San Luis, Puntarenas, MNCR) **74** *Anomala unilineata* (Parque Nacional Santa Rosa, Guanacaste, MNCR) **75** *Anomala valida* (Reserva Biológica Hitoy Cerere, Limón, MNCR) **76** *Anomala vallisneria* (Sector Las Pailas, Guanacaste, MNCR). Scale bars: 5 mm. Fig. 63 from Filippini et al. (2014); Figs 65, 74 from Filippini et al. (2015c); Figs 64, 67–69, 71–72, 76 from Filippini et al. (2015d).

**Figures 77–90.**
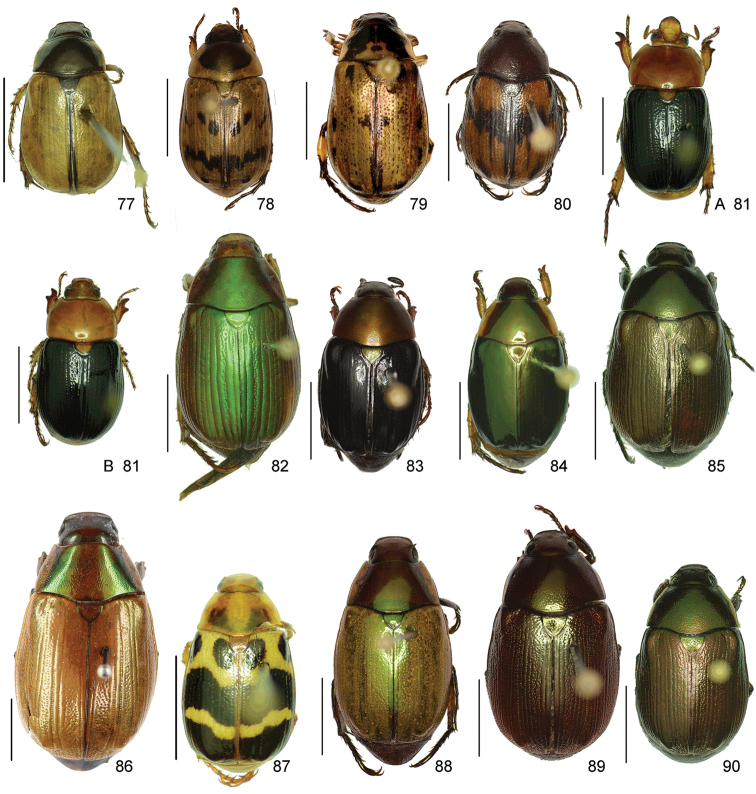
Habitus. **77** *Anomala veraecrucis* (Finca Jenny, Guanacaste, MNCR) **78** *Anomala volsellata* (Cerro Brujo, Puntarenas, MNCR) **79** *Anomala vulcanicola* (San Gerardo de Dota, San José, MNCR) **80** *Anomala zumbadoi* (Rancho quemado, Puntarenas, MNCR) **81** *Anomalorhina turrialbana*, A: male, B: female (Cabanga, Alajuela, CEUA, both) **82** *Callistethus calonotus* (Alto de Las Moras, Puntarenas, MNCR) **83** *Callistethus carbo* (Río San Lorenzo, Guanacaste, MNCR) **84** *Callistethus chlorotoides* (Reserva Biológica Hitoy Cerere, Limón, MNCR) **85** *Callistethus chontalensis* (El Copal, Cartago, CEUA) **86** *Callistethus chrysanthe* (Chiriqui, MNHUB) **87** *Callistethus chrysomelinus* (San Luis, Puntarenas, MNCR) **88** *Callistethus flavodorsalis* (Finca Cafrosa, Puntarenas, MNCR) **89** *Callistethus fuscorubens* (La Esquadra, Puntarenas, MNCR) **90** *Callistethus granulipygus* (Rancho Quemado, Puntarenas, MNCR). Scale bars: 5 mm. Figs 78, 80 from Filippini et al. (2014); Figs 83, 88–89 from Filippini et al. (2015a); Fig. 79 from Filippini et al. (2015d).

**Figures 91–105.**
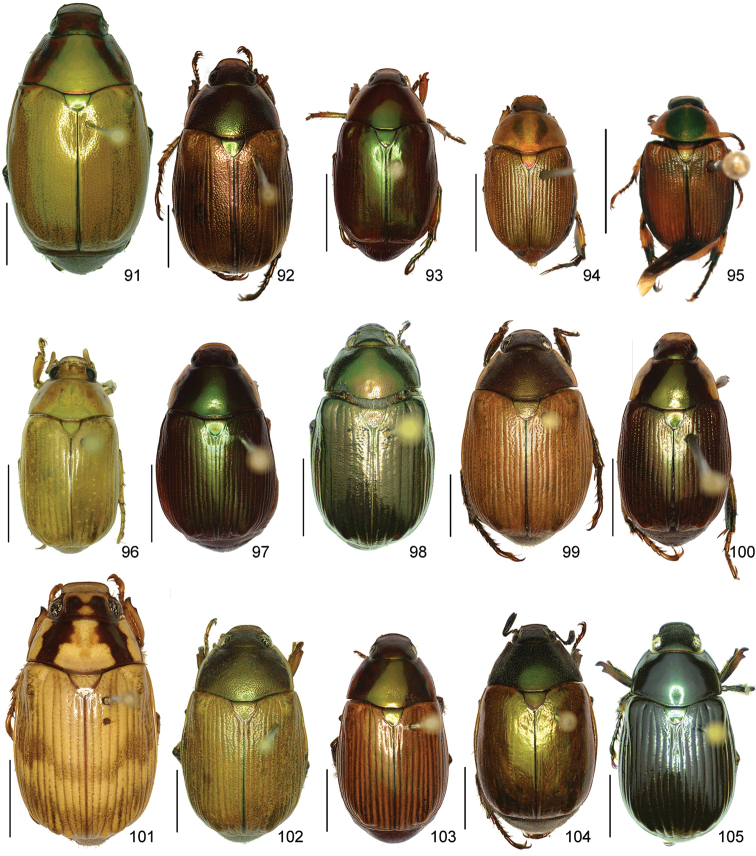
Habitus. **91** *Callistethus jordani* (Estación Cacao, Guanacaste, MNCR) **92** *Callistethus lativittis* (Río San Lorenzo, Guanacaste, MNCR) **93** *Callistethus levigatus* (Quebrada Segunda, Cartago, MNCR) **94** *Callistethus macroxantholeus* (Estación Pitilla, Guanacaste, MNCR) **95** *Callistethus microxantholeus* (Est. Pitilla, Guanacaste, MNCR) **96** *Callistethus mimeloides* (La Montura, San José, CEUA) **97** *Callistethus multiplicatus* (Sector Cerro Cocori, Limón, MNCR) **98** *Callistethus nicoya* (Estación Quebrada Bonita, Puntarenas, MNCR) **99** *Callistethus parapulcher* (Estación Altamira, Puntarenas, MNCR) **100** *Callistethus pseudocollaris* (Estación La Casona, Puntarenas, MNCR) **101** *Callistethus ruteloides* (holotype) **102** *Callistethus schneideri* (Estación Pitilla, Guanacaste, MNCR) **103** *Callistethus specularis* (Rio San Lorenzo, Guanacaste, MNCR) **104** *Callistethus stannibractea* (Estación Barva, Heredia, MNCR) **105** *Callistethus sulcans* (Reserva Biológica Hitoy Cerere, Limón). Scale bars: 5 mm. Figs 92–95, 97, 99–100, 103–104 from Filippini et al. (2015a); Fig. 101 from Filippini et al. (2015b).

**Figures 106–114.**
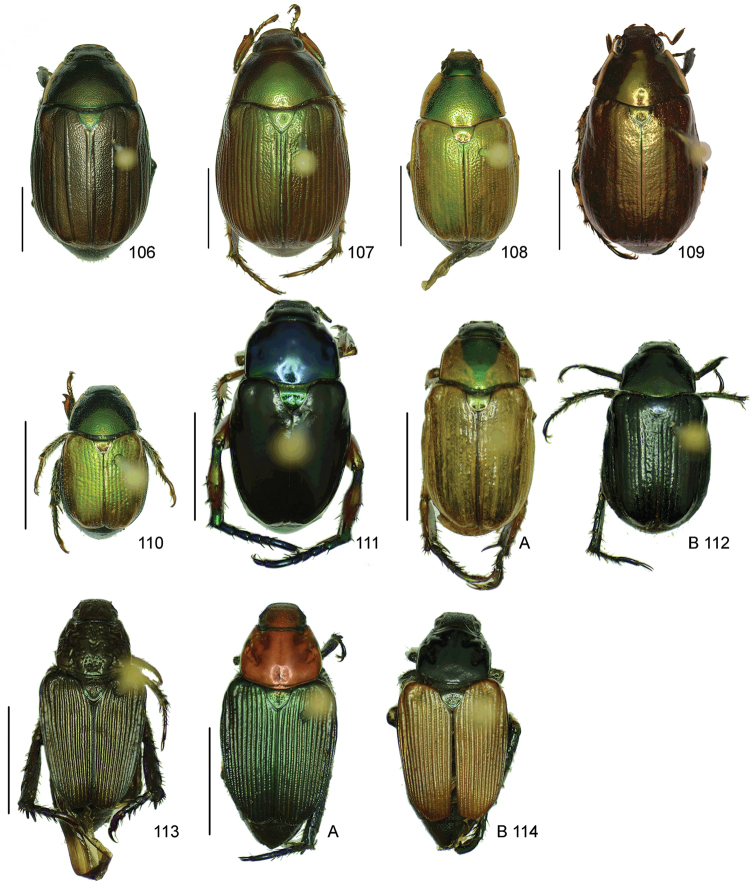
Habitus. **106** *Callistethus valdecostatus* (Estación Biológica Las Alturas, Puntarenas, MNCR) **107** *Callistethus vanpatteni* (Cinco Esquinas de Carrizal, Alajuela, MNCR) **108** *Callistethus xiphostethus* (Estación Las Pailas, Guanacaste, MNCR) **109** *Callistethus yalizo* (holotype) **110** *Moroniella nitidula* (Zarcero, Alajuela, MNCR) **111** *Strigoderma auriventris* (Sector San Ramón de dos ríos, Alajuela, MNCR) **112** *Strigoderma biolleyi*, showing variable colorations (A: Reserva Tapantí, Cartago, MNCR; B: Macizo de la Muerte, Cartago, MNCR) **113** *Strigoderma nodulosa* (Urbanización El Colegio, Puntarenas, MNCR) **114** *Strigoderma sulcipennis*, showing variable colorations (A: Finca Jenny, Guanacaste, MNCR; B: San Luis, Puntarenas, MNCR). Scale bars: 5 mm. Fig. 106 from Filippini et al. (2015a); Fig. 109 from Filippini et al. (2015b).

**Figures 115–123.**
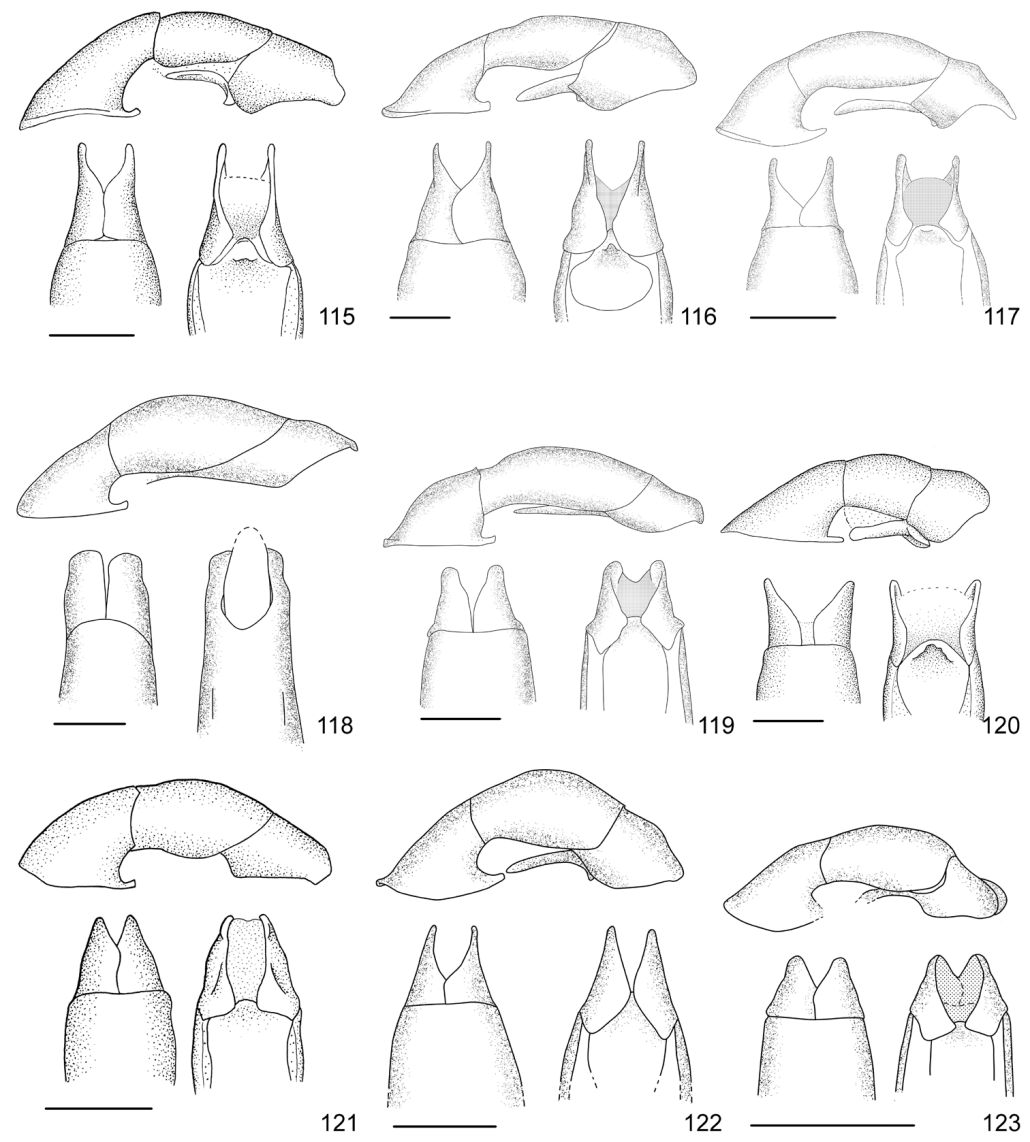
Shape of aedeagus, lateral view (top), dorsal view (bottom left), ventral view (bottom right). **115** *Anomala aereiventris* (Parque Nacional Tapantí, Cartago, MNCR) **116** *Anomala aglaos* (Isla Bonita, Alajuela, CEUA) **117** *Anomala antica* (Estación Palo Verde, Guanacaste, MNCR) **118** *Anomala arara* (Estación Cabro Muco, Guanacaste, CEUA) **119** *Anomala arthuri* (Estación Maritza, Guanacaste, MNCR) **120** *Anomala aspersa* (Villa Mills, Cartago, MNCR) **121** *Anomala atrivillosa* (Estación Barva, Heredia, MNCR) **122** *Anomala balzapambae* (Rancho Quemado, Puntarenas, MNCR) **123** *Anomala calligrapha* (Cabro Muco, Guanacaste, CEUA). Scale bars: 1 mm. Figs 118–119 modified from Filippini et al. (2014); Figs 116–117 modified from Filippini et al. (2015b); Figs 115, 120–121 from Filippini et al. (2015d).

**Figures 124–132.**
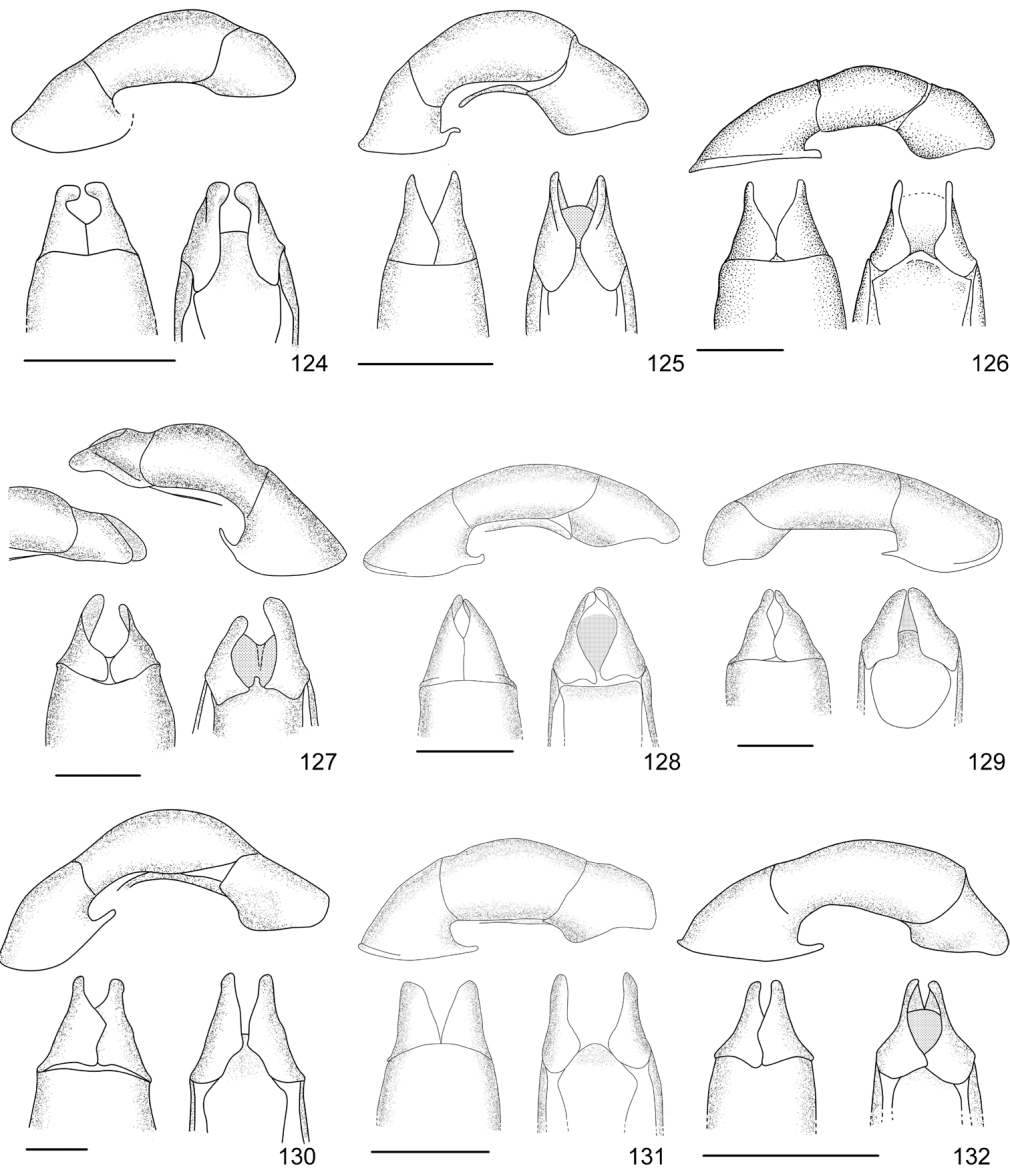
Shape of aedeagus, lateral view (top), dorsal view (bottom left), ventral view (bottom right). **124** *Anomala chiriquina* (Finca Cafrosa, Puntarenas, MNCR) **125** *Anomala cinaedias* (San Luis, Puntarenas, MNCR) **126** *Anomala clarivillosa* (La Esperanza, Cartago, CEUA) **127** *Anomala clathrata*, below lateral view: detail of parameres on the other side (Cerro Bitárkara, Limón, CEUA) **128** *Anomala coffea* (Estación Pitilla, Guanacaste, MNCR) **129** *Anomala cupreovariolosa* (Las Cruces, Puntarenas, MNCR) **130** *Anomala cupricollis* (Finca San Gabriel, Alajuela, MNCR) **131** *Anomala cyclops* (Cerro El Hacha, Guanacaste, MNCR) **132** *Anomala discoidalis* (Estación Cuatro Esquinas, Limón, MNCR). Scale bars: 1 mm. Figs 127, 129 modified from Filippini et al. (2014); Figs 128, 131 from Filippini et al. (2015c); Fig. 126 from Filippini et al. (2015d).

**Figures 133–141.**
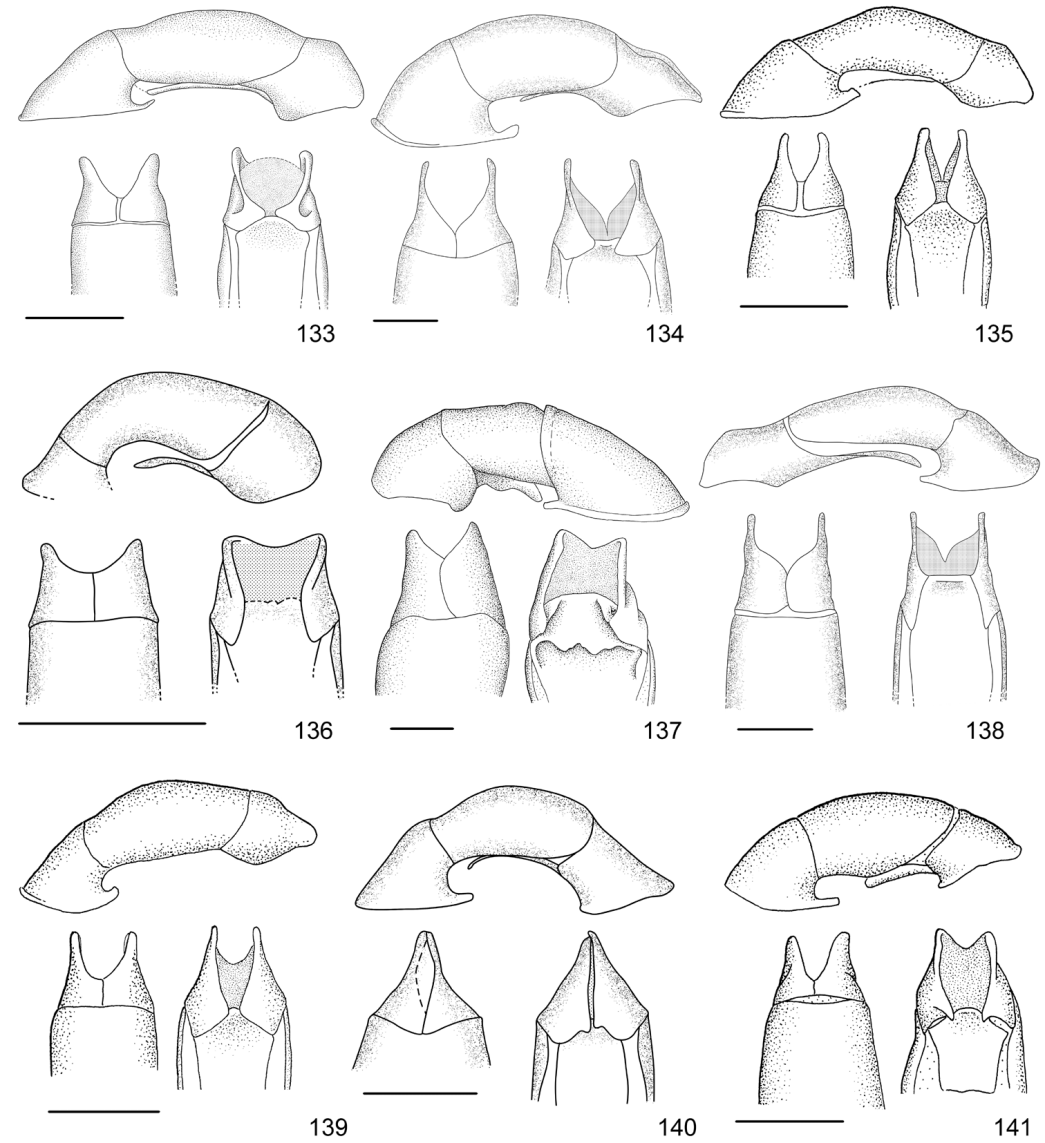
Shape of aedeagus, lateral view (top), dorsal view (bottom left), ventral view (bottom right). **133** *Anomala divisa* (Cinco esquinas de Carrizal, Alajuela, MNCR) **134** *Anomala estrella* (Estación La Casona, Puntarenas, MNCR) **135** *Anomala eucoma* (San José, San José, MUCR) **136** *Anomala eulissa* (Estación Biológica La Selva, Heredia, MNCR) **137** *Anomala eusticta* (Estación La Casona, Puntarenas, MNCR) **138** *Anomala ferrea* (Las Cruces, Puntarenas, MNCR) **139** *Anomala flavacoma* (Estación Cabro Muco, Guanacaste, CEUA) **140** *Anomala foraminosa* (Estación Hitoy Cerere, Limón, MNCR) **141** *Anomala globulata* (Macizo de la Muerte, Cartago, MNCR). Scale bars: 1 mm. Fig. 139 from Filippini et al. (2013), Fig. 138 from Filippini et al. (2014); Fig. 134 from Filippini et al. (2015b); Fig. 133 from Filippini et al. (2015c); Figs 137, 141 from Filippini et al. (2015d).

**Figures 142–150.**
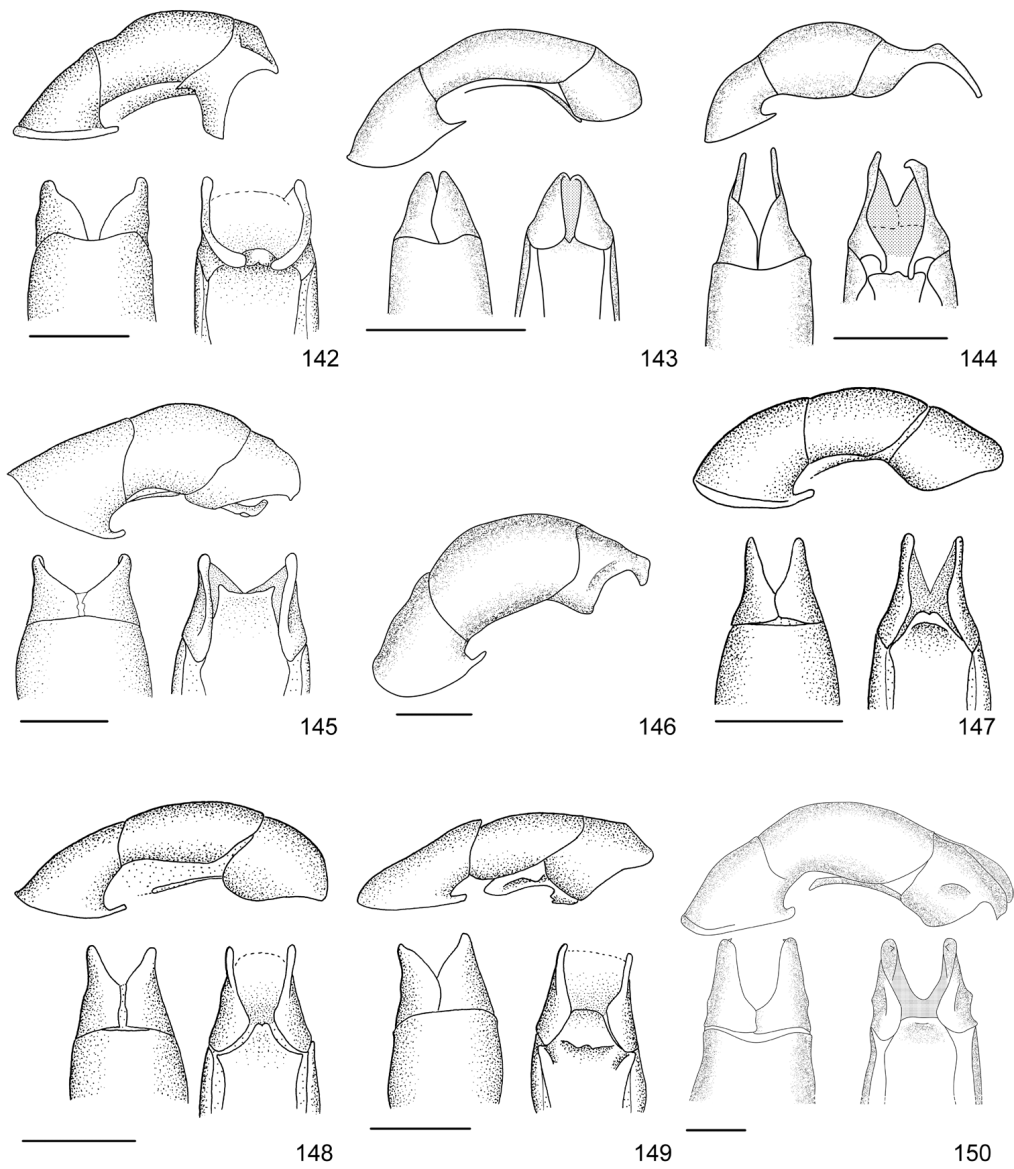
Shape of aedeagus, lateral view (top), dorsal view (bottom left), ventral view (bottom right). **142** *Anomala hiata* (Estación Pittier, Puntarenas, MNCR) **143** *Anomala histrionella* (Bahía Santa Elena, Guanacaste, MNCR) **144** *Anomala hoppi* (Las Cruces, Puntarenas, MNCR) **145** *Anomala inbio* (Volcán Tenorio, Guanacaste, CEUA) **146** *Anomala jansoni* (Monte Rotondo, Costa Rica, MNHUB) **147** *Anomala latifalculata* (Zona Protectora Cerros de la Carpintera, Cartago, MNCR) **148** *Anomala leopardina* (Finca Cafrosa, Puntarenas, MNCR) **149** *Anomala levicollis* (Cerro Montezuma, Alajuela, CEUA) **150** *Anomala limon* (Estación Hitoy Cerere, Limón, MNCR). Scale bars: 1 mm. Figs 146, 150 modified from Filippini et al. (2015b); Figs 142, 145, 147–149 from Filippini et al. (2015d).

**Figures 151–159.**
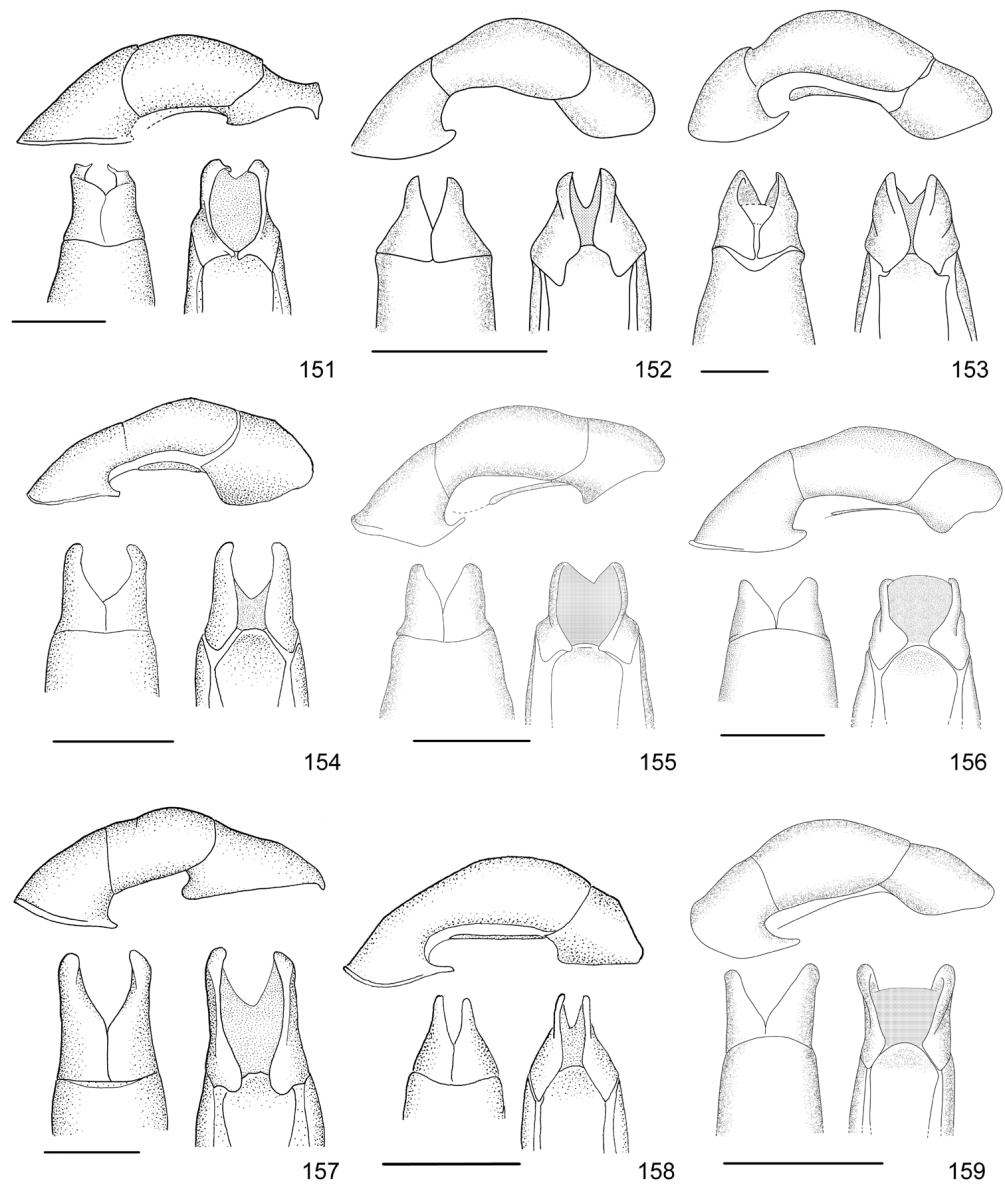
Shape of aedeagus, lateral view (top), dorsal view (bottom left), ventral view (bottom right). **151** *Anomala longisacculata* (La Montura, San José, CEUA) **152** *Anomala ludoviciana* (Finca Jenny, Guanacaste, MNCR) **153** *Anomala megalia* (Manzanillo, Limón, MNCR) **154** *Anomala megaparamera* (Estación Cuatro Esquinas, Limón, MNCR) **155** *Anomala mersa* (Sector Palo Verde, Guanacaste, MNCR) **156** *Anomala mesosticta* (Los Arbolitos, heredia, MNCR) **157** *Anomala m-fuscum* (Río Grande de Orosí, Cartago, MNCR) **158** *Anomala moroni* (Estación Palo Verde, Guanacaste, MNCR) **159** *Anomala nigroflava* (Río Rincon, Puntarenas, MNCR). Scale bars: 1 mm. Fig. 154 from Filippini et al. (2013), Fig. 159 from Filippini et al. (2014); Figs 155–156 from Filippini et al. (2015c); Figs 151, 157 from Filippini et al. (2015d); Fig. 158 from Filippini et al. (2015e).

**Figures 160–168.**
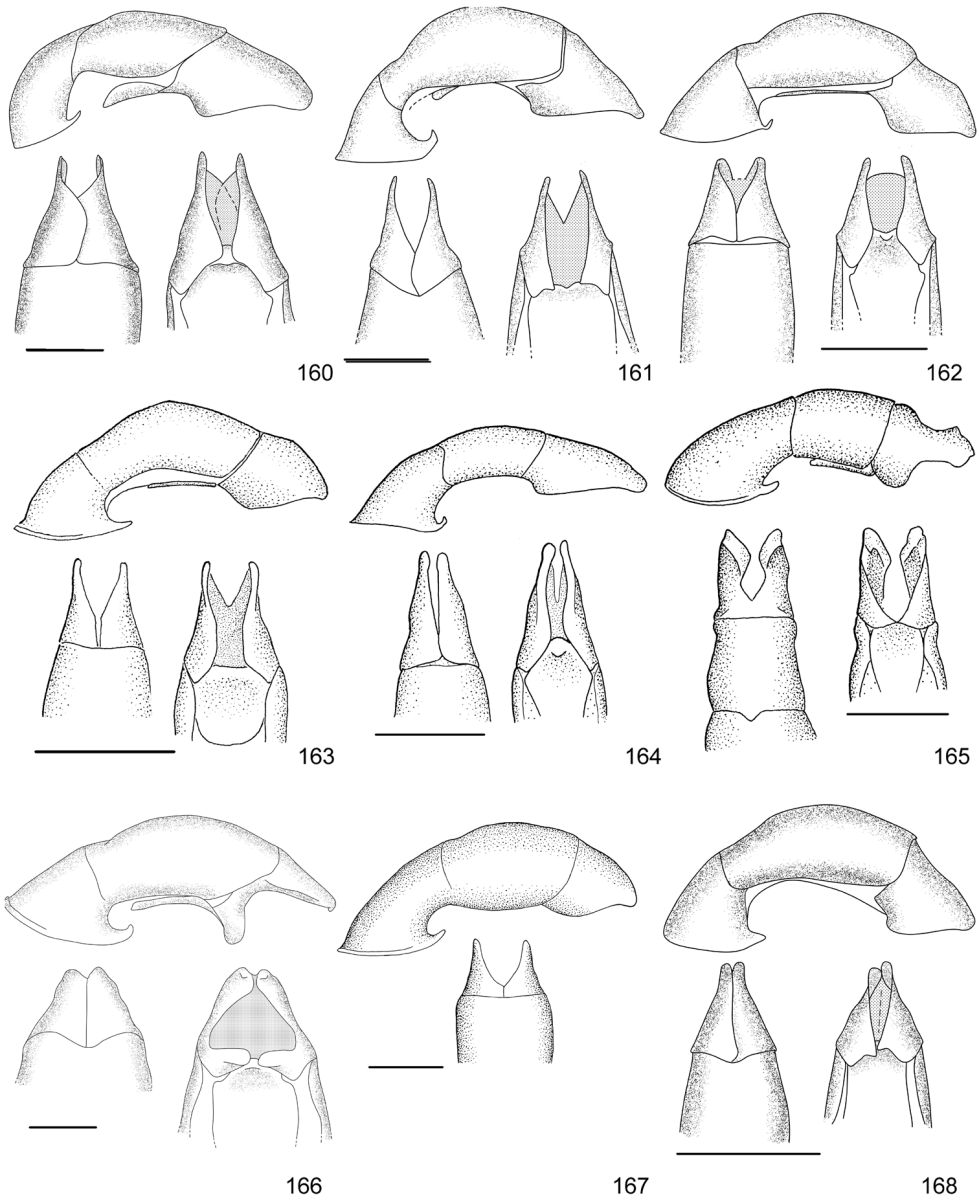
Shape of aedeagus, lateral view (top), dorsal view (bottom left), ventral view (bottom right). **160** *Anomala obovata* (Quebrada Kuisa, Limón, MNCR) **161** *ochrogastra* (Finca Cafrosa, Puntarenas, MNCR) **162** *Anomala ochroptera* (La Maritza, Guanacaste, MNCR) **163** *Anomala parvaeucoma* (Estación Sirena, Puntarenas, MNCR) **164** *Anomala perspicax* (La Esperanza, Cartago, CEUA) **165** *Anomala piccolina* (Estación Biológica Las Alturas, Puntarenas, MNCR) **166** *Anomala pincelada* (Finca Jenny, Guanacaste, MNCR) **167** *Anomala polygona* (Escazu, Costa Rica, MNHUB) **168** *Anomala popayana* (Río Banano, Limón, MNCR). Scale bars: 1 mm. Fig. 168 modified from Filippini et al. (2014); Figs 160, 166 from Filippini et al. (2015b); Figs 164–165, 167 from Filippini et al. (2015d); Fig. 163 from Filippini et al. (2015e).

**Figures 169–177.**
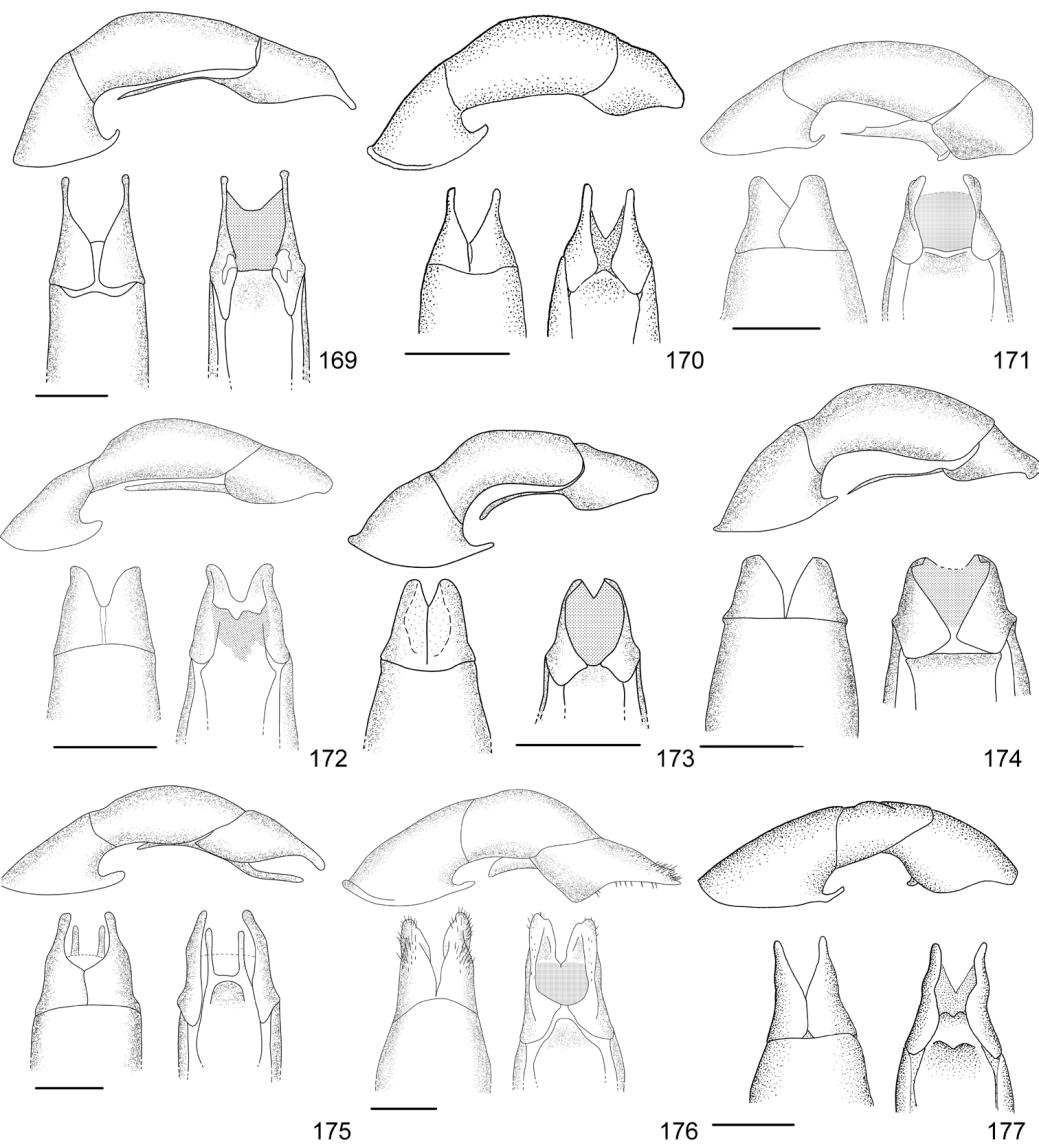
Shape of aedeagus, lateral view (top), dorsal view (bottom left), ventral view (bottom right). **169** *Anomala praecellens* (Orosilito, Guanacaste, CEUA) **170** *Anomala pseudoeucoma* (Estación Hitoy Cerere, Limón, MNCR) **171** *Anomala quiche* (Estación Maritza, Guanacaste, MNCR) **172** *Anomala robiginosa* (Zarcero, Alajuela, MNCR) **173** *Anomala ruatana* (Playa Naranjo, Guanacaste, MNCR) **174** *Anomala semicincta* (Albergue Heliconias, Alajuela, CEUA) **175** *Anomala semilla* (Albergue Heliconias, Alajuela, CEUA) **176** *Anomala solisi* (Amubri, Limón, MNCR) **177** *Anomala stillaticia* (La Catarata, Cartago, MNCR). Scale bars: 1 mm. Fig. 170 from Filippini et al. (2013); Figs 174–176 modified from Filippini et al. (2014); Fig. 171 modified from Filippini et al. (2015b); Fig. 172 from Filippini et al. (2015c); Fig. 177 from Filippini et al. (2015d).

**Figures 178–186.**
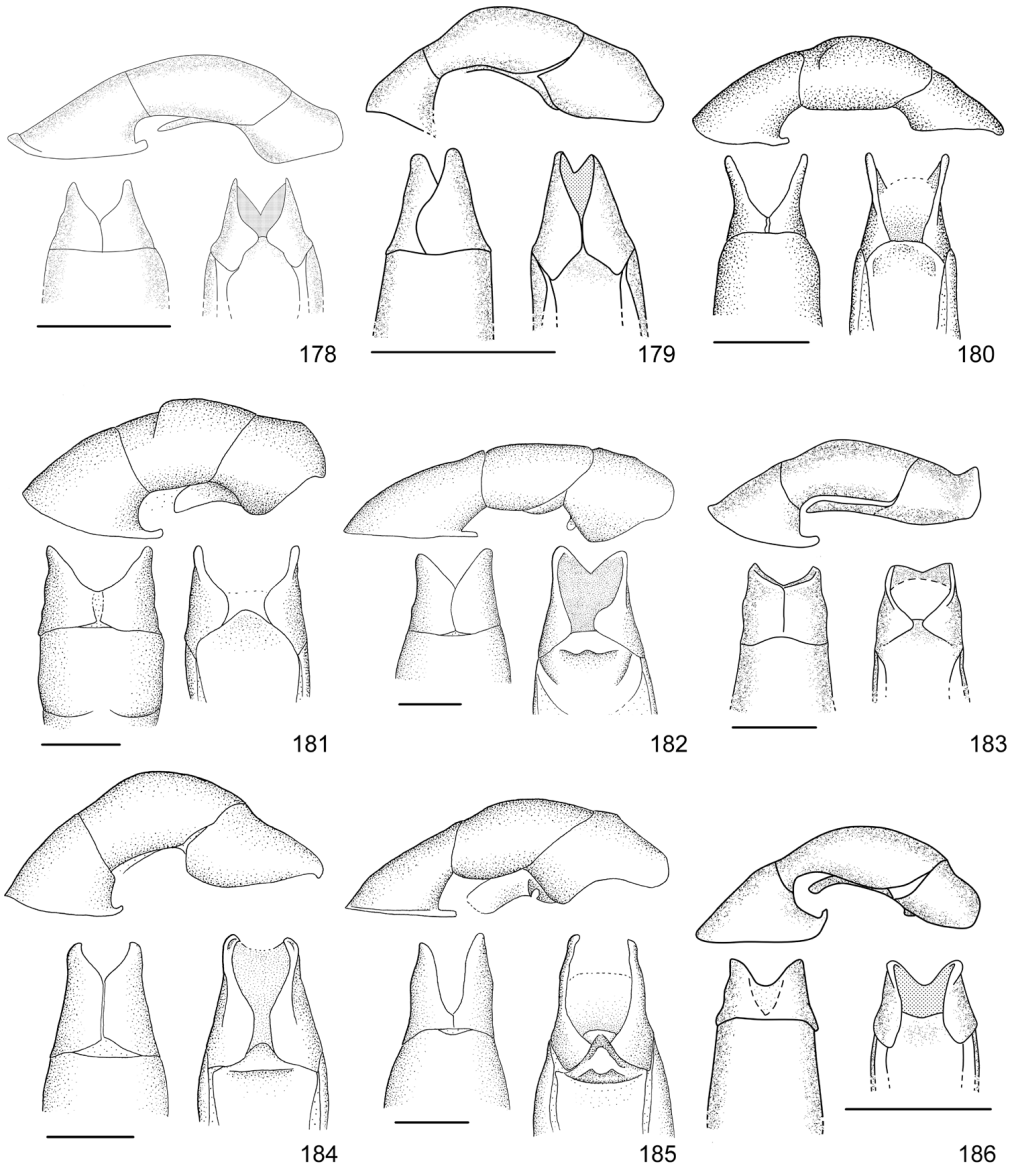
Shape of aedeagus, lateral view (top), dorsal view (bottom left), ventral view (bottom right). **178** *Anomala strigodermoides* (Bijagua, Alajuela, MNCR) **179** *Anomala subaenea* (Estación Maritza, Guanacaste, MNCR) **180** *Anomala subridens* (Reserva Forestal Río Macho, Cartago, MNCR) **181** *Anomala subusta* (Estación Cacao, Guanacaste, MNCR) **182** *Anomala tenoriensis* (Parque Nacional Volcán Tenorio, Alajuela, MNCR) **183** *Anomala testaceipennis* (Boca Tapada, Alajuela, MNCR) **184** *Anomala trapezifera* (Parque Nacional Tapantí, Cartago, CEUA) **185** *Anomala tuberculata* (Isla Bonita, Alajuela, CEUA) **186** *Anomala undulata* (Estación Cacao, Guanacaste, MNCR). Scale bars: 1 mm. Fig. 183 modified from Filippini et al. (2014); Fig. 178 from Filippini et al. (2015c); Figs 180–182, 184–185 from Filippini et al. (2015d).

**Figures 187–195.**
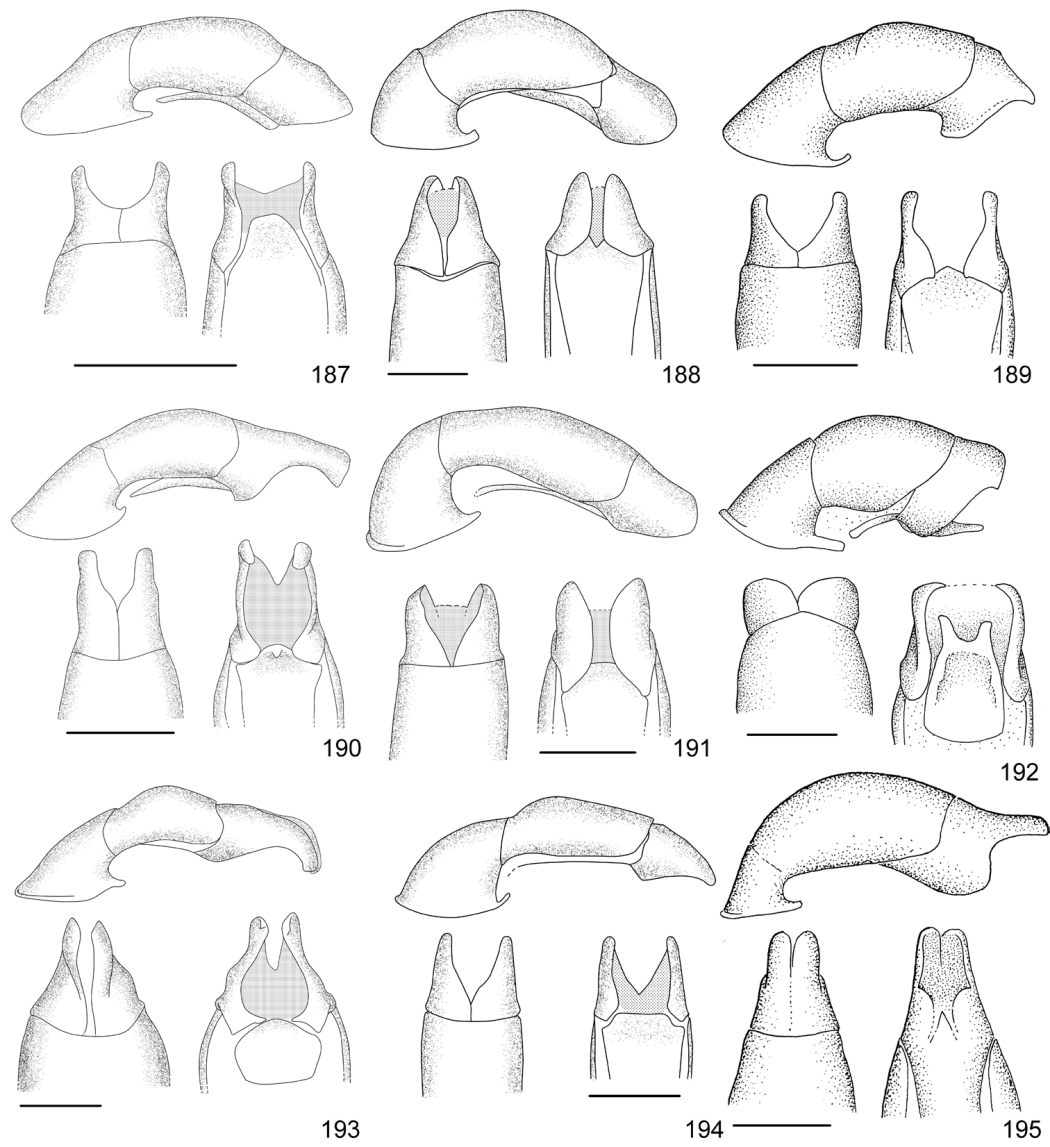
Shape of aedeagus, lateral view (top), dorsal view (bottom left), ventral view (bottom right). **187** *Anomala unilineata* (Parque Nacional Santa Rosa, Guanacaste, MNCR) **188** *Anomala valida* (La Cruz, Guanacaste, MNCR) **189** *Anomala vallisneria* (Sector Las Pailas, Guanacaste, MNCR) **190** *Anomala veraecrucis* (Finca Jenny, Guanacaste, MNCR) **191** *Anomala volsellata* (Las Quebraditas, Puntarenas, MNCR) **192** *Anomala vulcanicola* (San Gerardo de Dota, San José, MNCR) **193** *Anomala zumbadoi* (Rancho quemado, Puntarenas, MNCR) **194** *Anomalorhina turrialbana* (Cabanga, Alajuela, CEUA) **195** *Callistethus calonotus* (Alto de Las Moras, Puntarenas, MNCR). Scale bars: 1 mm. Figs 191, 193 from Filippini et al. (2014); Fig. 195 modified from Filippini et al. (2015a); Fig. 187 from Filippini et al. (2015c); Figs 189, 192 from Filippini et al. (2015d).

**Figures 196–204.**
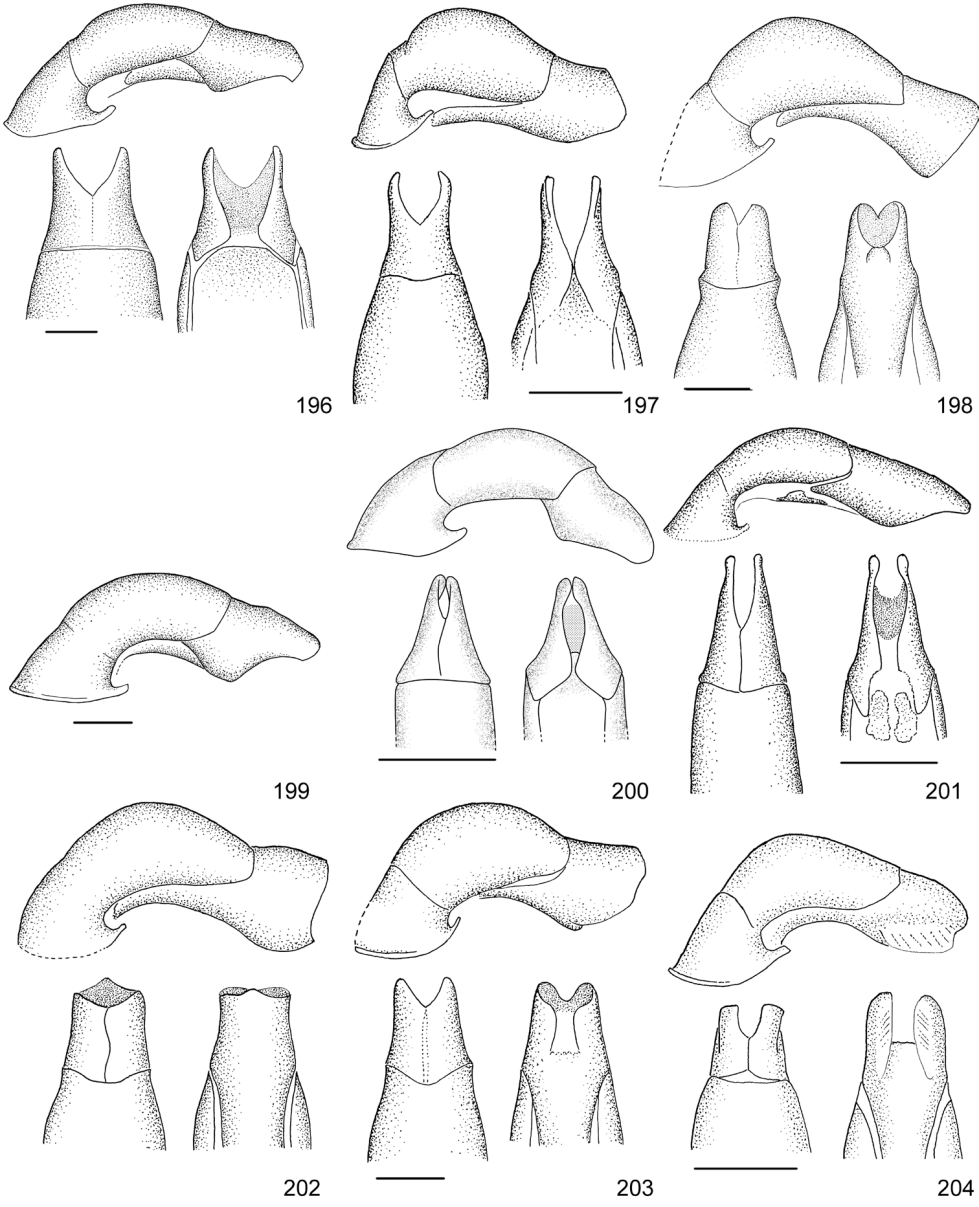
Shape of aedeagus, lateral view (top), dorsal view (bottom left), ventral view (bottom right). **196** *Callistethus carbo* (Río San Lorenzo, Guanacaste, MNCR) **197** *Callistethus chlorotoides* (Estación Hitoy Cerere, Limón, MNCR) **198** *Callistethus chontalensis* (El Copal, Cartago, CEUA) **199** *Callistethus chrysanthe* (Chiriqui, MNHUB) **200** *Callistethus chrysomelinus* (San Luis, Puntarenas, MNCR) **201** *Callistethus flavodorsalis* (Finca Cafrosa, Puntarenas, MNCR) **202** *Callistethus fuscorubens* (La Esquadra, Puntarenas, MNCR) **203** *Callistethus granulipygus* (Rancho Quemado, Puntarenas, MNCR) **204** *Callistethus jordani* (Estación Cacao, Guanacaste, MNCR). Scale bars: 1 mm. Figs 196–199, 201–204 modified from Filippini et al. (2015a).

**Figures 205–213.**
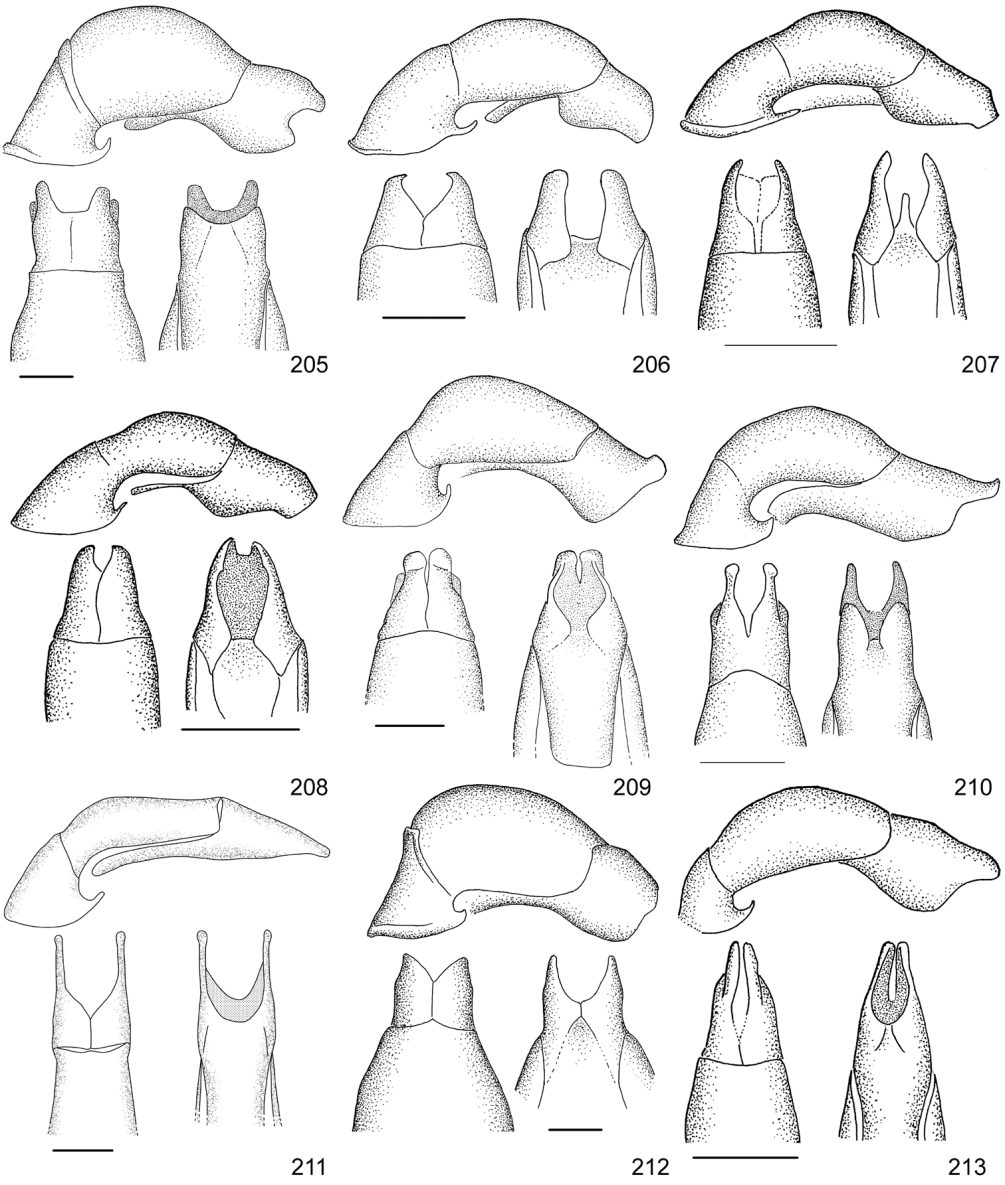
Shape of aedeagus, lateral view (top), dorsal view (bottom left), ventral view (bottom right) **205** *Callistethus lativittis* (Dos de Tilaran, Guanacaste, MNCR) **206** *Callistethus levigatus* (Estación La Casona, Puntarenas, MNCR) **207** *Callistethus macroxantholeus* (Sector Cerro Cocori, Limón, MNCR) **208** *Callistethus microxantholeus* (Est. Pitilla, Guanacaste, MNCR) **209** *Callistethus mimeloides* (La Montura, San José, CEUA) **210** *Callistethus multiplicatus* (Sector Cerro Cocori, Limón, MNCR) **211** *Callistethus nicoya* (Estación Quebrada Bonita, Puntarenas, MNCR) **212** *Callistethus parapulcher* (Estación Pittier, Puntarenas, MNCR) **213** *Callistethus pseudocollaris* (Estación La Casona, Puntarenas, MNCR). Scale bars: 1 mm. Figs 205–210, 212–213 modified from Filippini et al. (2015a).

**Figures 214–222.**
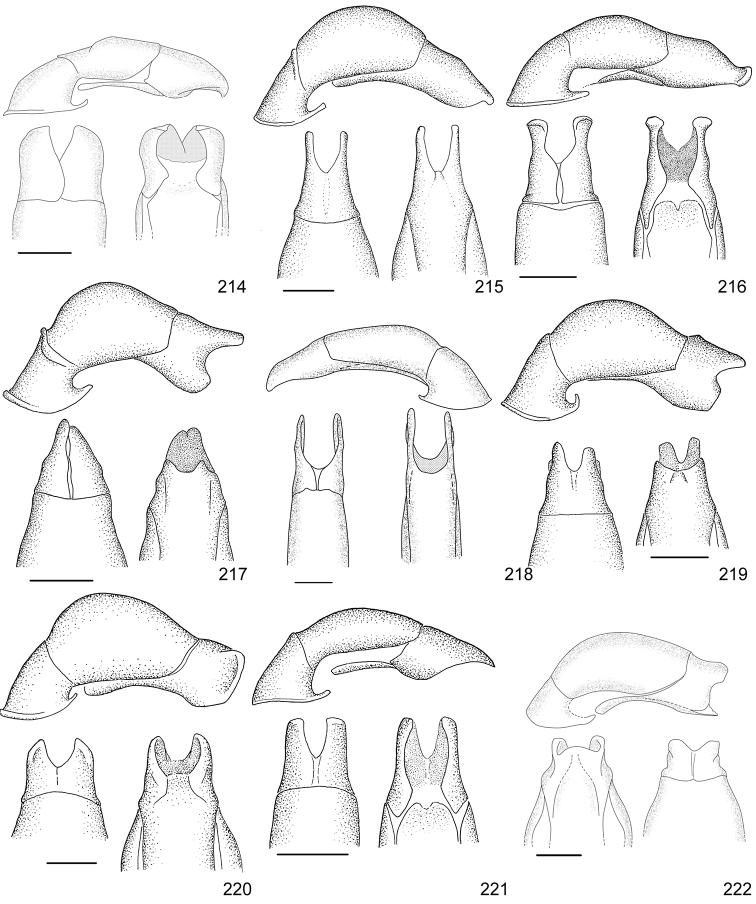
Shape of aedeagus, lateral view (top), dorsal view (bottom left), ventral view (bottom right). **214** *Callistethus ruteloides* (holotype) **215** *Callistethus schneideri* (Albergue Heliconias, Alajuela, MNCR) **216** *Callistethus specularis* (Costa Rica, BMNH) **217** *Callistethus stannibractea* (Estación Barva, Heredia, MNCR) **218** *Callistethus sulcans* (Reserva Biológica Hitoy Cerere, Limón, MNCR) **219** *Callistethus valdecostatus* (Alto de las Moras, Puntarenas, MNCR) **220** *Callistethus vanpatteni* (Cinco Esquinas de Carrizal, Alajuela, MNCR) **221** *Callistethus xiphostethus* (Los Ángeles, Heredia, MNCR) **222** *Callistethus yalizo* (Cerro Chompipe, Heredia). Scale bars: 1 mm. Figs 215–217, 219–221 modified from Filippini et al. (2015a); Figs 214, 222 from Filippini et al. (2015b).

**Figures 223–227.**
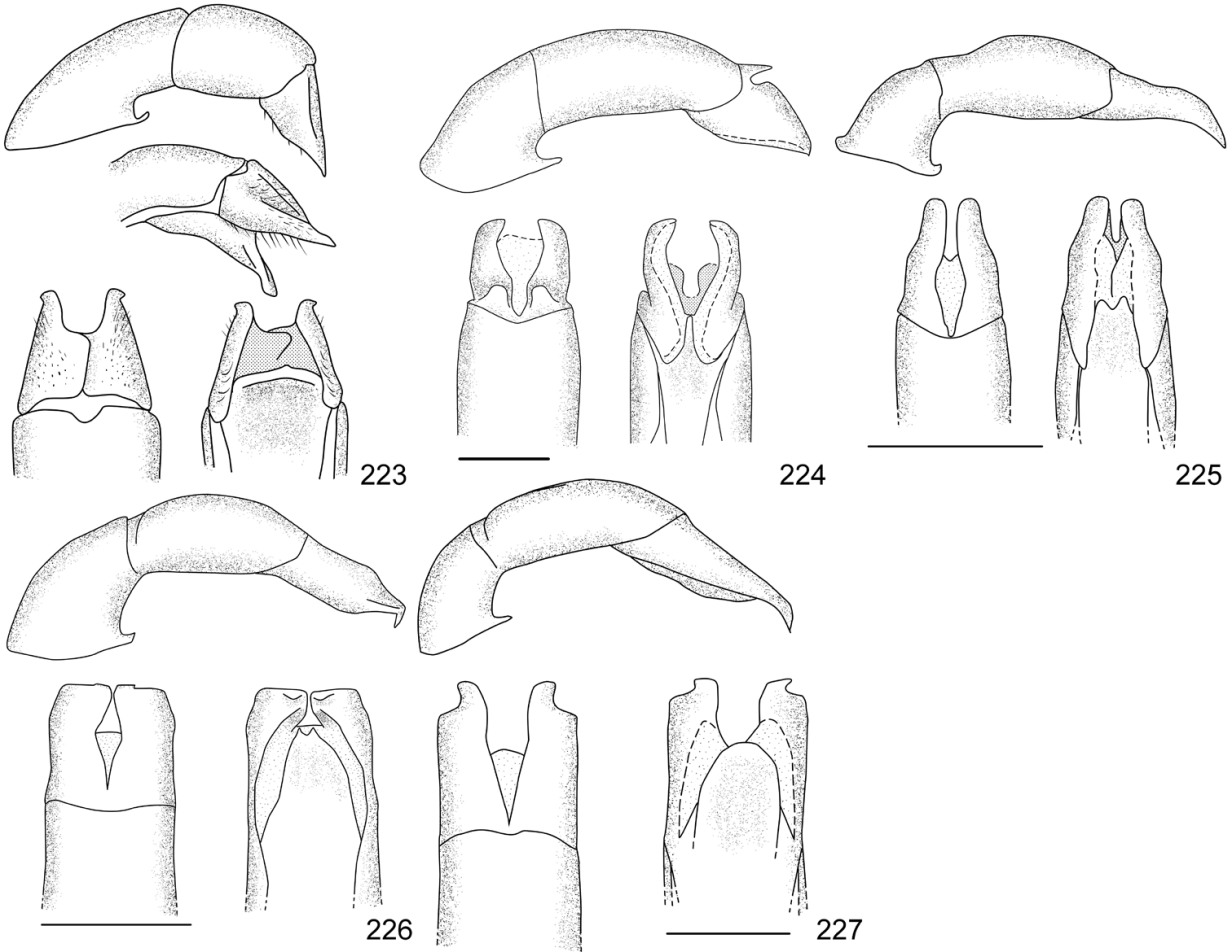
Shape of aedeagus, lateral view (top), dorsal view (bottom left), ventral view (bottom right). **223** *Moroniella nitidula*, below lateral view: detail of parameres when endophallus is everted (Lateral view: Guatemala, MNHUB; other: Zarcero, Alajuela, MNCR) **224** *Strigoderma auriventris* (Sector San Ramón de dos ríos, Alajuela, MNCR) **225** *Strigoderma biolleyi* (Macizo de la Muerte, Cartago, MNCR) **226** *Strigoderma nodulosa* (Estación Quebrada Bonita, Puntarenas, MNCR) **227** *Strigoderma sulcipennis* (Finca Jenny, Guanacaste, MNCR). Scale bars: 1 mm.

**Figures 228–233.**
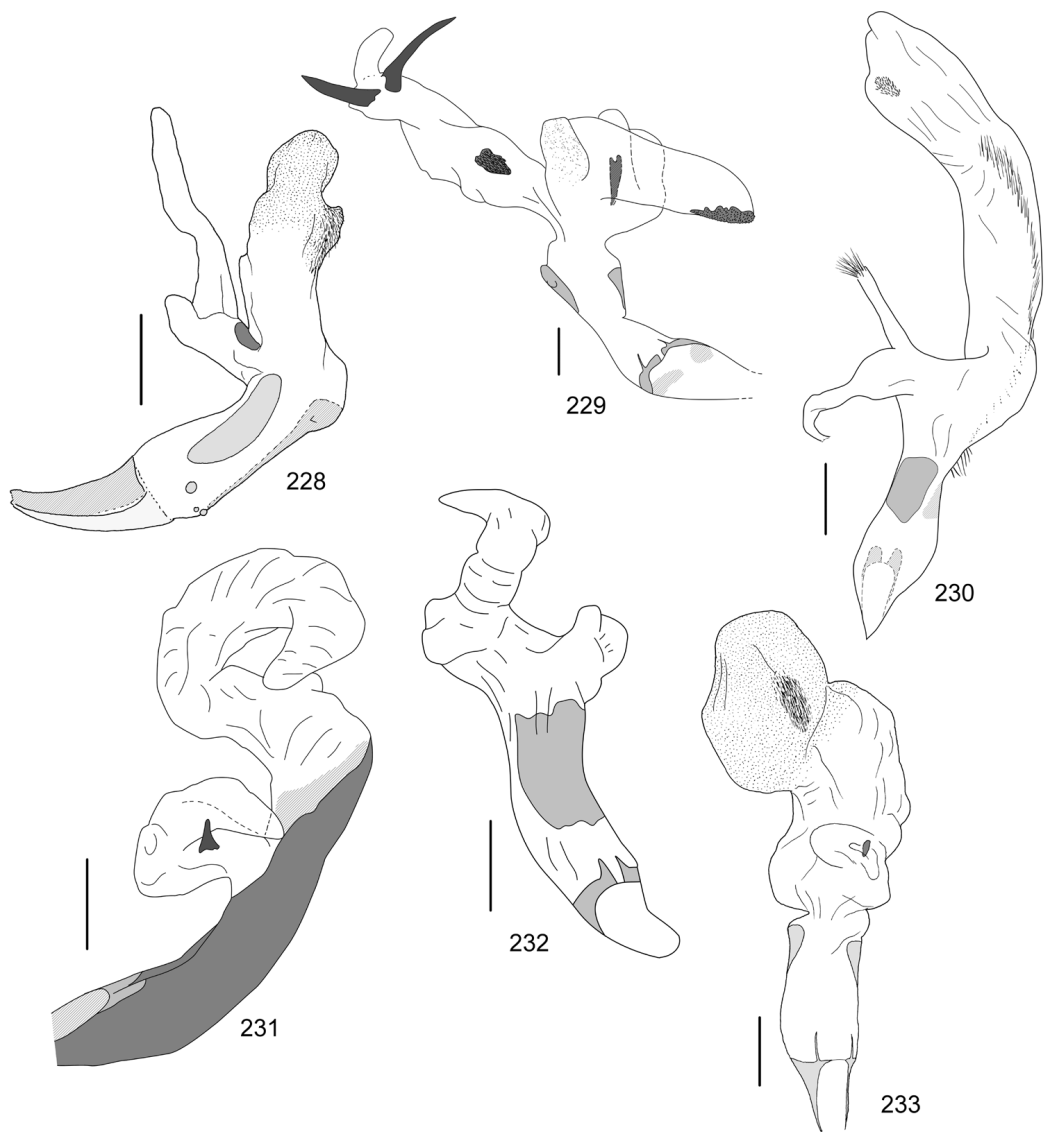
Endophallus of **228** *Anomala aereiventris* (Parque Nacional Tapantí, Cartago, MNCR) **229** *Anomala aglaos* (Isla Bonita, Alajuela, CEUA) **230** *Anomala antica* (Parque Nacional Santa Rosa, Guanacaste, MNCR) **231** *Anomala arara* (Albergue Heliconias, Alajuela, CEUA) **232** *Anomala arthuri* (Estación Maritza, Guanacaste, MNCR) **233** *Anomala aspersa* (Villa Mills, Cartago, MNCR). Scale bars: 1 mm. Fig. 232 from Filippini et al. (2014), Fig. 229 from Filippini et al. (2015b); Figs 228, 233 from Filippini et al. (2015d).

**Figures 234–240.**
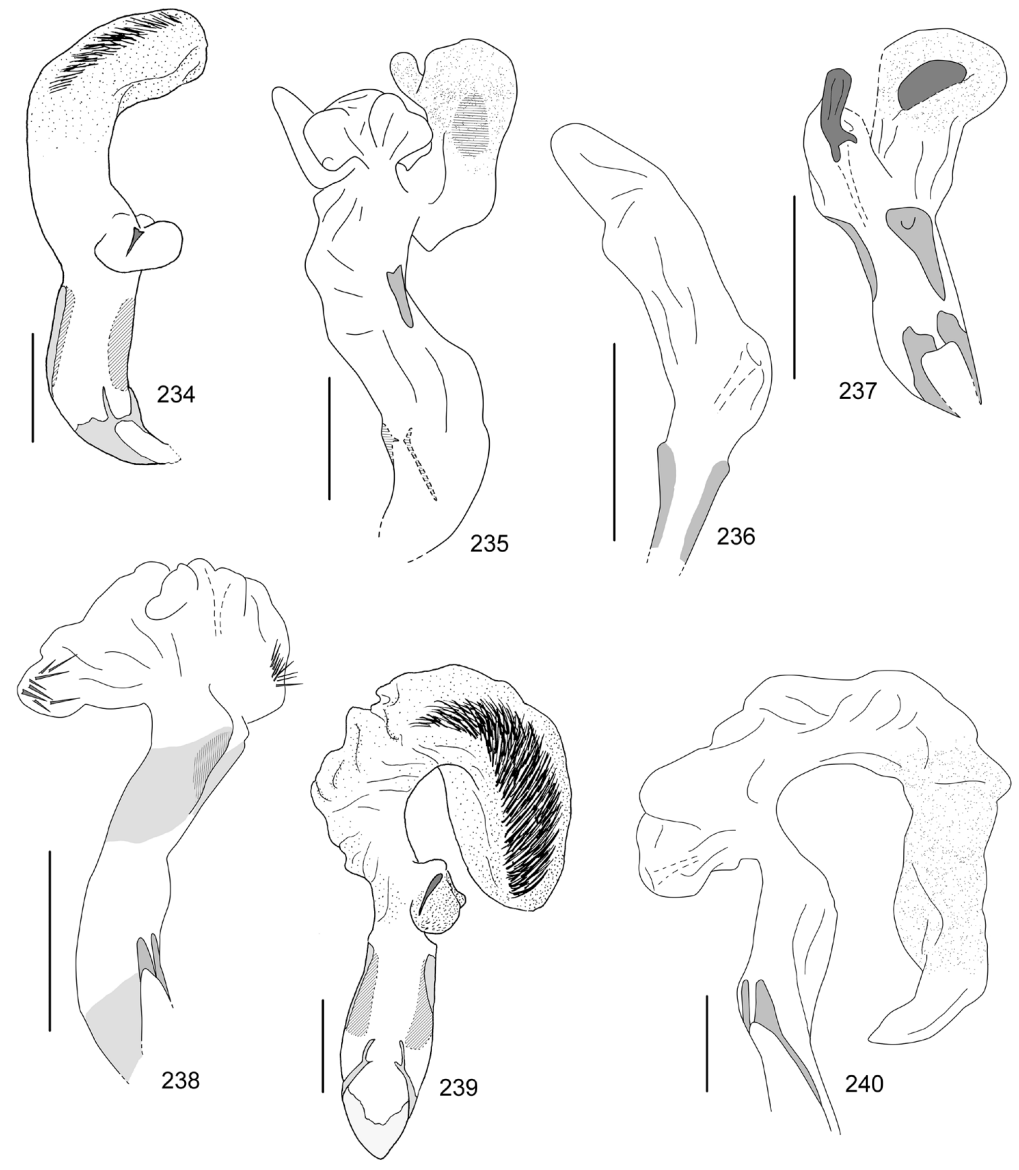
Endophallus of **234** *Anomala atrivillosa* (Estación Barva, Heredia, MNCR) **235** *Anomala balzapambae* (Rancho Quemado, Puntarenas, MNCR) **236** *Anomala calligrapha* (Cabro Muco, Guanacaste, CEUA) **237** *Anomala chiriquina* (Estación Biológica Las Alturas, Puntarenas, MNCR) **238** *Anomala cinaedias* (San Luis, Puntarenas, MNCR) **239** *Anomala clarivillosa* (La Esperanza del Guarco, Cartago, CEUA) **240** *Anomala clathrata* (Albergue Heliconias, Alajuela, CEUA). Scale bars: 1 mm. Figs 234, 239 from Filippini et al. (2015d).

**Figures 241–247.**
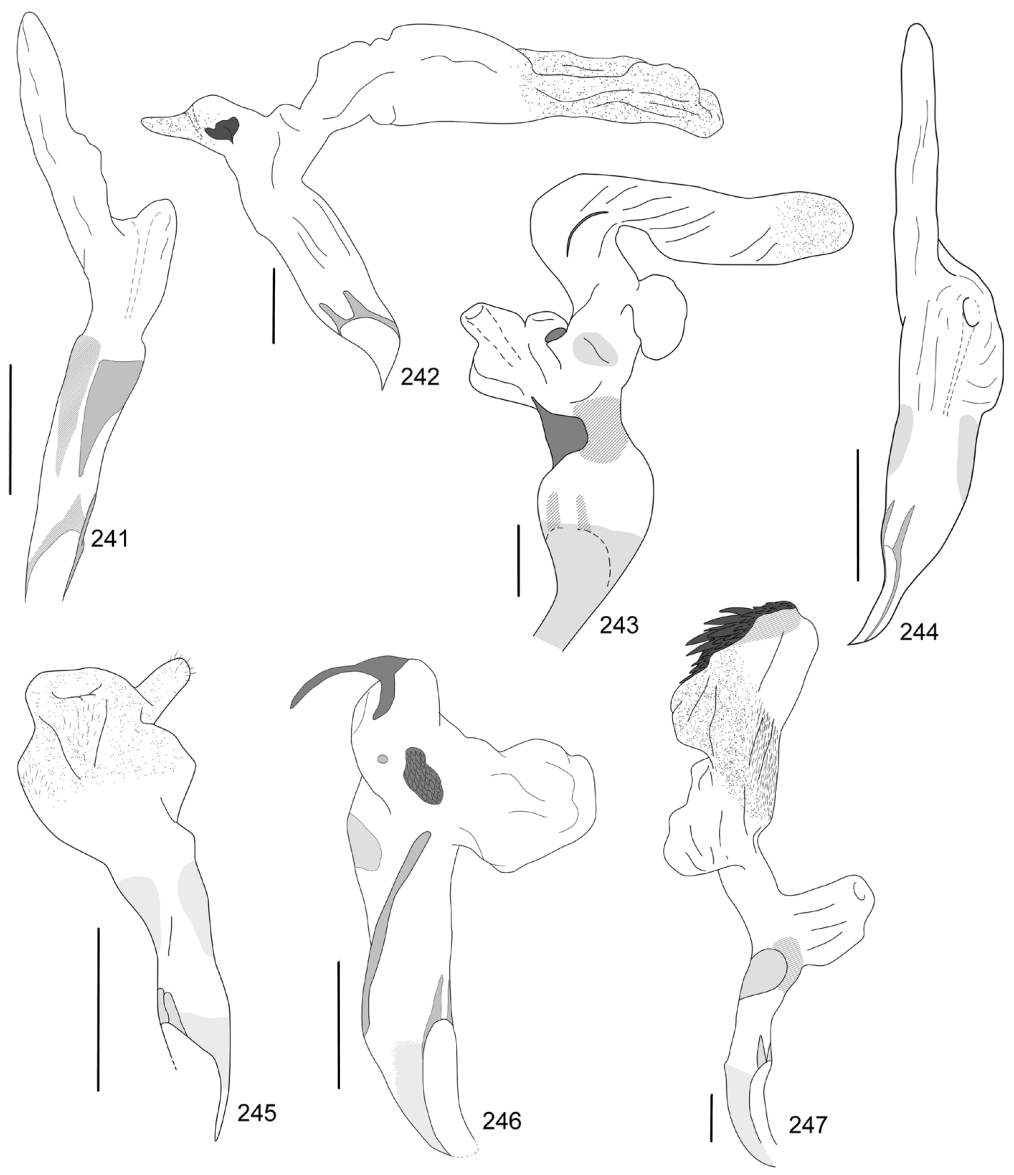
Endophallus of **241** *Anomala coffea* (Estación Pitilla, Guanacaste, MNCR) **242** *Anomala cupreovariolosa* (Zona Protectora Las Tablas, Puntarenas, MNCR) **243** *Anomala cupricollis* (Las Cruces, Puntarenas, MNCR) **244** *Anomala cyclops* (Finca Jenny, Guanacaste, MNCR) **245** *Anomala discoidalis* (Estación Cuatro Esquinas, Limón, MNCR) **246** *Anomala divisa* (Cinco esquinas de Carrizal, Alajuela, MNCR) **247** *Anomala estrella* (Hacienda Tiquires, San José, MNCR). Scale bars: 1 mm. Fig. 242 from Filippini et al. (2014); Fig. 247 from Filippini et al. (2015b); Figs 241, 244, 246 from Filippini et al. (2015c).

**Figures 248–254.**
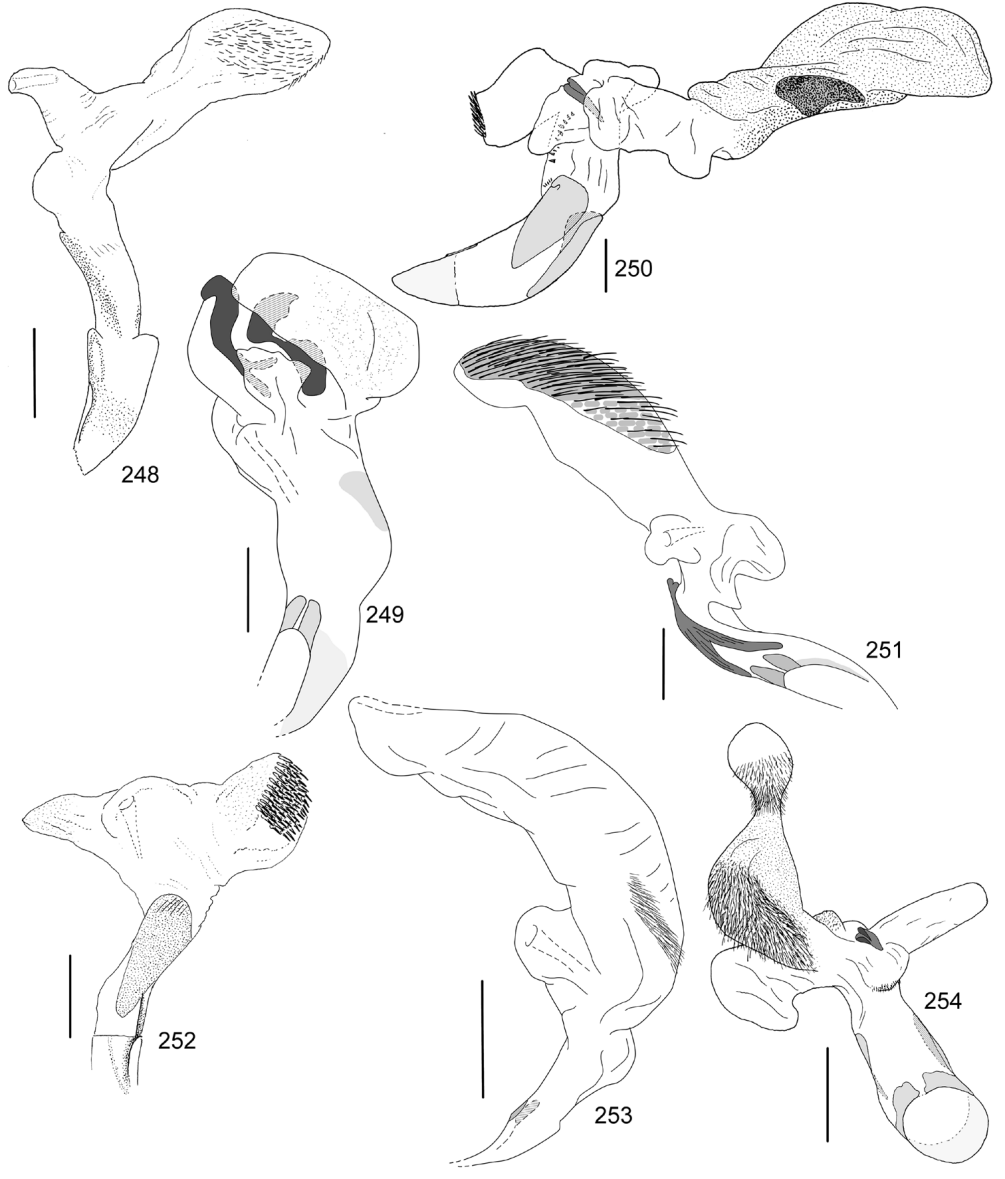
Endophallus of **248** *Anomala eucoma* (San José, San José, MUCR) **249** *Anomala eulissa* (Sector Cedrales de la Rita, Limón, MNCR) **250** *Anomala eusticta* (Estación La Casona, Puntarenas, MNCR) **251** *Anomala ferrea* (Las Cruces, Puntarenas, MNCR) **252** *Anomala flavacoma* (Estación Cabro Muco, Guanacaste, CEUA) **253** *Anomala foraminosa* (Estación Hitoy Cerere, Limón, MNCR) **254** *Anomala globulata* (reconstructed from two specimens: Macizo de la Muerte and Reserva forestal Río Macho, Cartago, MNCR). Scale bars: 1 mm. Figs 248, 252 from Filippini et al. (2013); Fig. 251 from Filippini et al. (2014); Figs 250, 254 from Filippini et al. (2015d).

**Figures 255–261.**
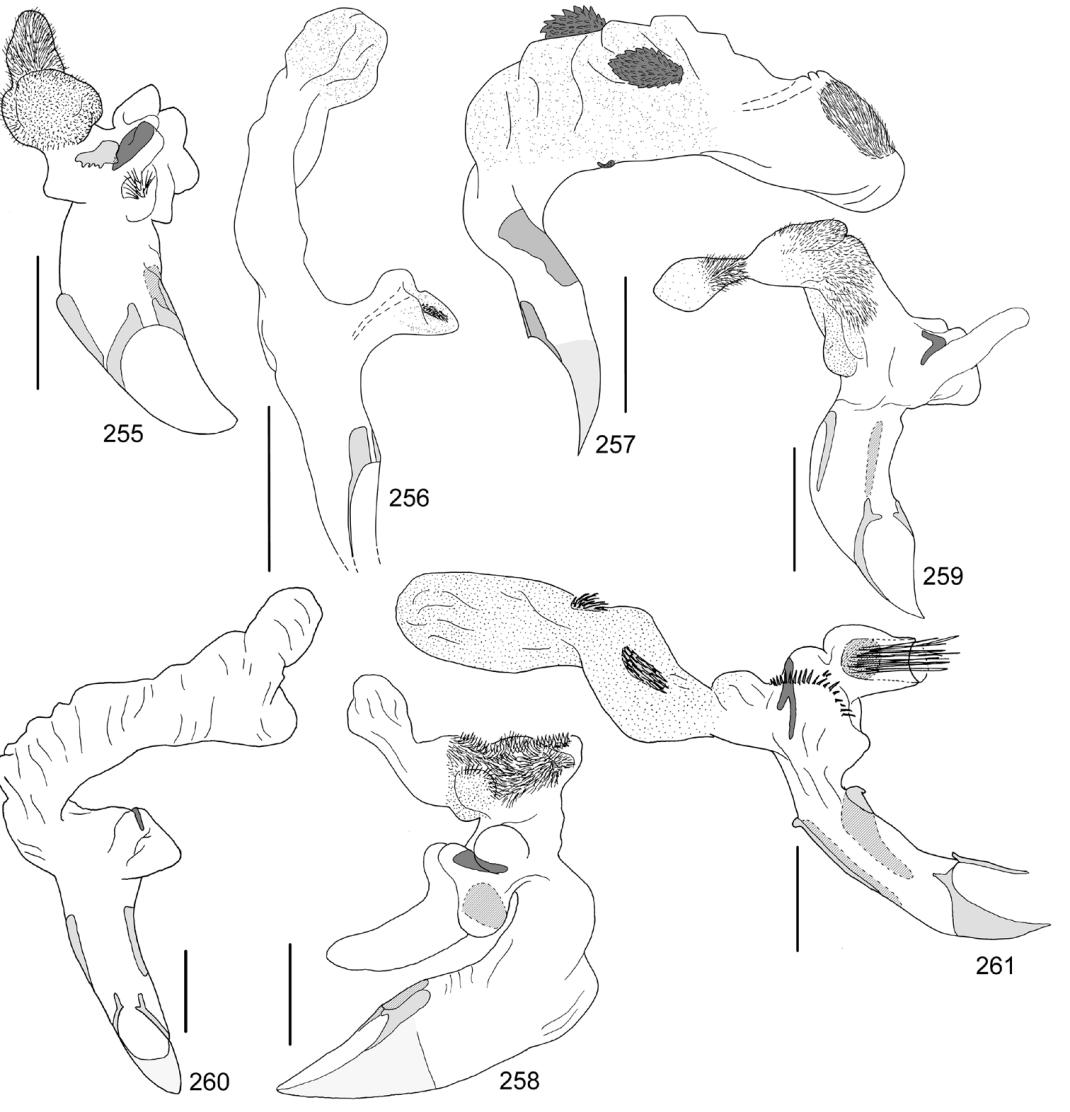
Endophallus of **255** *Anomala hiata* (Estación Pittier, Puntarenas, MNCR) **256** *Anomala histrionella* (Estación Murcielago, Guanacaste, MNCR) **257** *Anomala hoppi*, there is variability in the size and number (1–3) of patches of spines (Las Cruces, Puntarenas, MNCR) **258** *Anomala inbio* (Volcán Tenorio, Guanacaste, CEUA) **259** *Anomala latifalculata* (Zona Protectora Cerros de la Carpintera, Cartago, MNCR) **260** *Anomala leopardina* (Buenos Aires, Puntarenas, MNCR) **261** *Anomala levicollis* (Estación La Casona, Puntarenas, MNCR). Scale bars: 1 mm. Figs 255, 258–261 from Filippini et al. (2015d).

**Figures 262–267.**
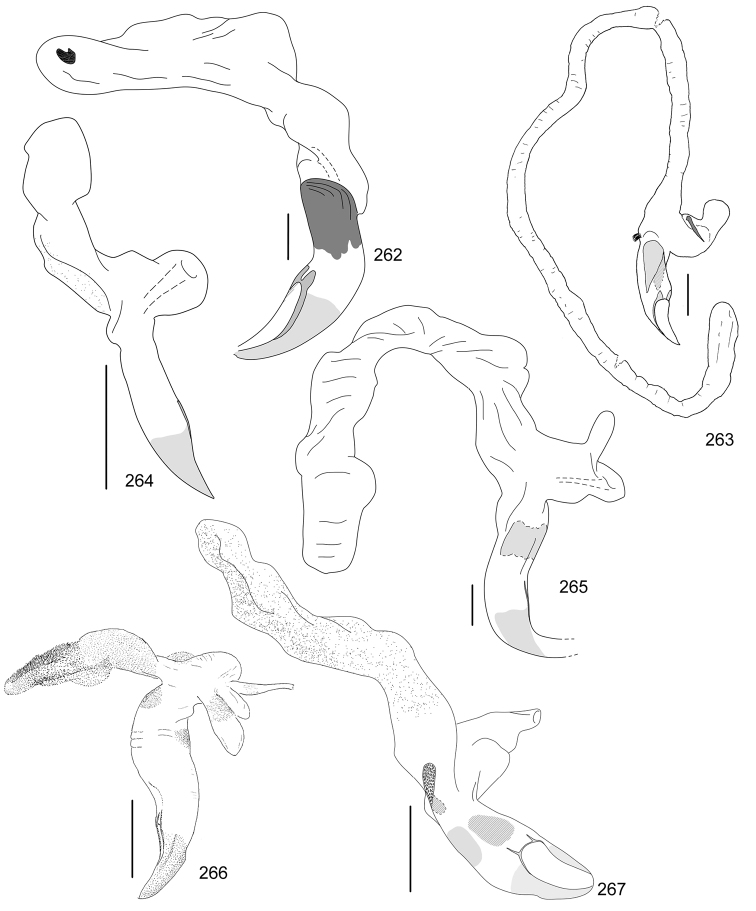
Endophallus of **262** *Anomala limon* (Estación Hitoy Cerere, Limón, MNCR) **263** *Anomala longisacculata* (Cabanga, Alajuela, CEUA) **264** *Anomala ludoviciana* (Parque Nacional Santa Rosa, Guanacaste, MNCR) **265** *Anomala megalia* (Cerro Tortuguero, Limón, MNCR) **266** *Anomala megaparamera* (Estación Cuatro Esquinas, Limón, MNCR) **267** *Anomala mersa* (Sector Palo Verde, Guanacaste, MNCR). Scale bars: 1 mm. Figures 266 from Filippini et al. (2013); Fig. 262 from Filippini et al. (2015b); Fig. 267 from Filippini et al. (2015c); Fig. 263 from Filippini et al. (2015d).

**Figures 268–276.**
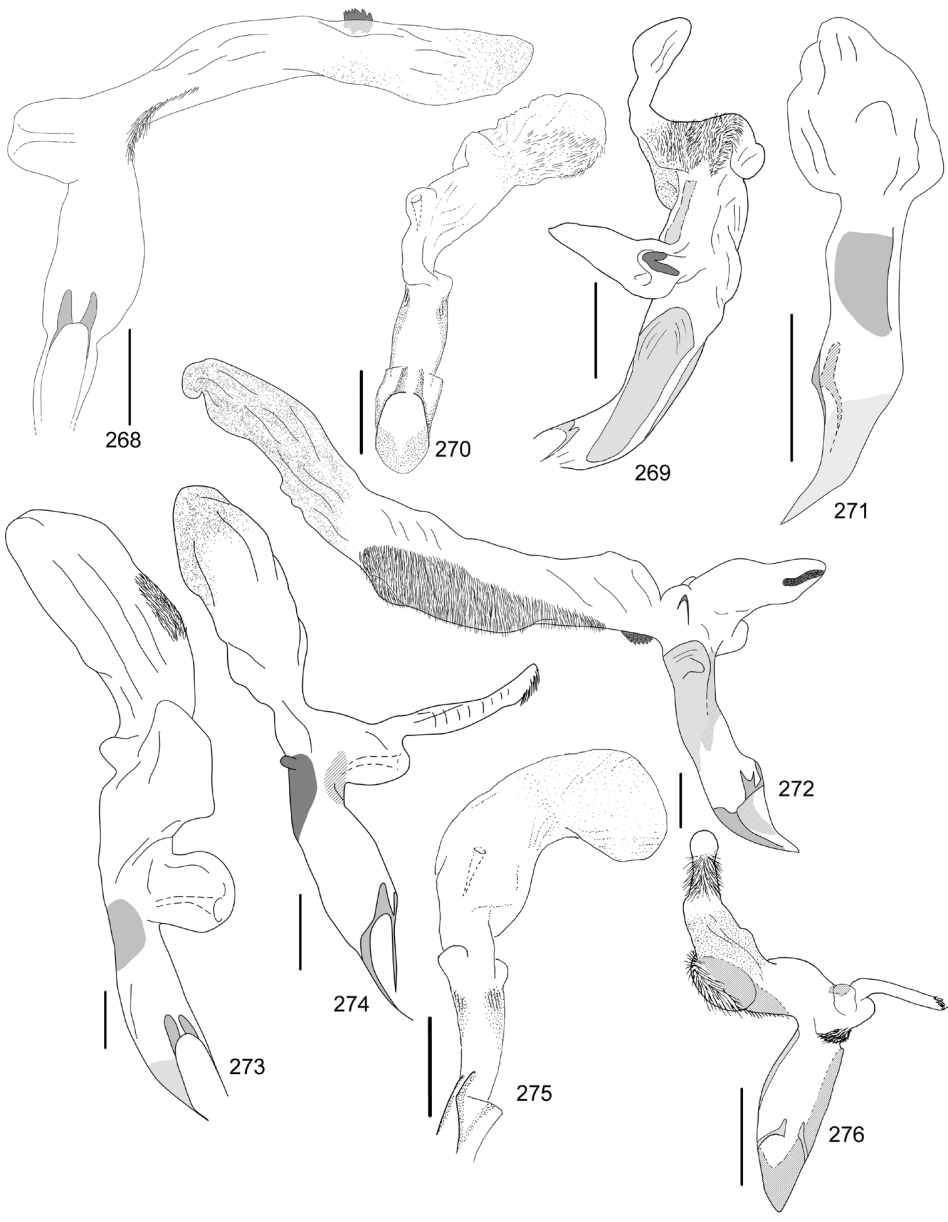
Endophallus of **268** *Anomala mesosticta* (Los Arbolitos, heredia, MNCR) **269** *Anomala m-fuscum* (La Esperanza del Guarco, Cartago, MNCR) **270** *Anomala moroni* (Estación Las Pailas, Guanacaste, MNCR) **271** *Anomala nigroflava* (Río Rincon, Puntarenas, MNCR) **272** *Anomala obovata* (Cerro Chompipe, Heredia, MNCR) **273** *Anomala ochrogastra* (Estación Las Alturas, Puntarenas, MNCR) **274** *Anomala ochroptera* (La Maritza, Guanacaste, MNCR) **275** *Anomala parvaeucoma* (Estación Sirena, Puntarenas, MNCR) **276** *Anomala perspicax* (Buenos Aires, Puntarenas, MNCR). Scale bars: 1 mm. Figures 271 from Filippini et al. (2014); Fig. 272 from Filippini et al. (2015b); Fig. 268 from Filippini et al. (2015c); Figs 269, 276 from Filippini et al. (2015d); Figs 270, 275 from Filippini et al. (2015e).

**Figures 277–283.**
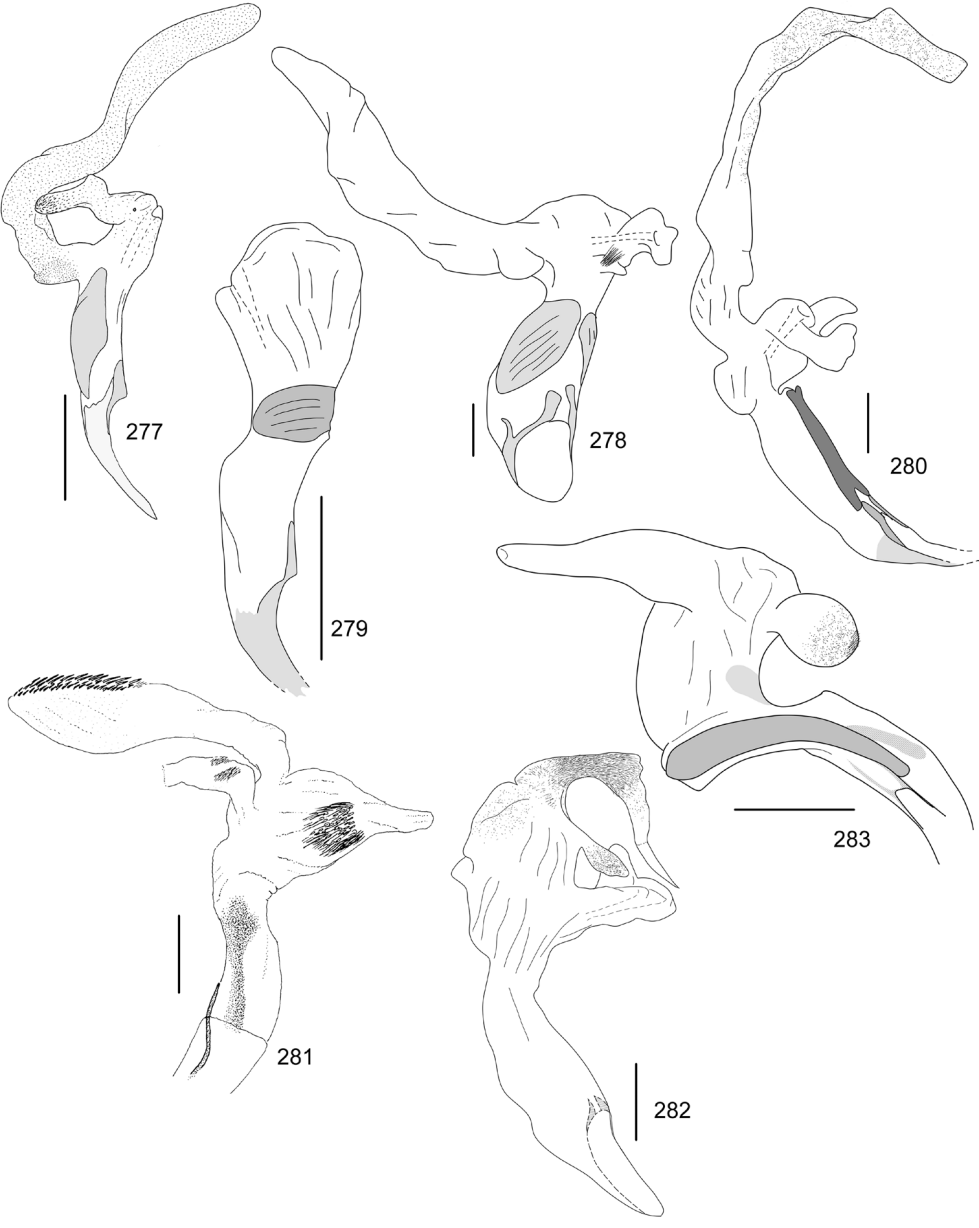
Endophallus of **277** *Anomala piccolina* (Estación Biológica Las Alturas, Puntarenas, MNCR) **278** *Anomala pincelada* (Cuajiniquil, Guanacaste, MNCR) **279** *Anomala popayana* (Reserva Biológica Hitoy Cerere, Limón, MNCR) **280** *Anomala praecellens* (Orosilito, Guanacaste, CEUA) **281** *Anomala pseudoeucoma* (Estación Hitoy Cerere, Limón, MNCR) **282** *Anomala quiche* (Estación Maritza, Guanacaste, MNCR) **283** *Anomala robiginosa* (Zarcero, Alajuela, MNCR). Scale bars: 1 mm. Figures 281 from Filippini et al. (2013); Fig. 278 from Filippini et al. (2015b); Fig. 283 from Filippini et al. (2015c); Fig. 277 from Filippini et al. (2015d).

**Figures 284–290.**
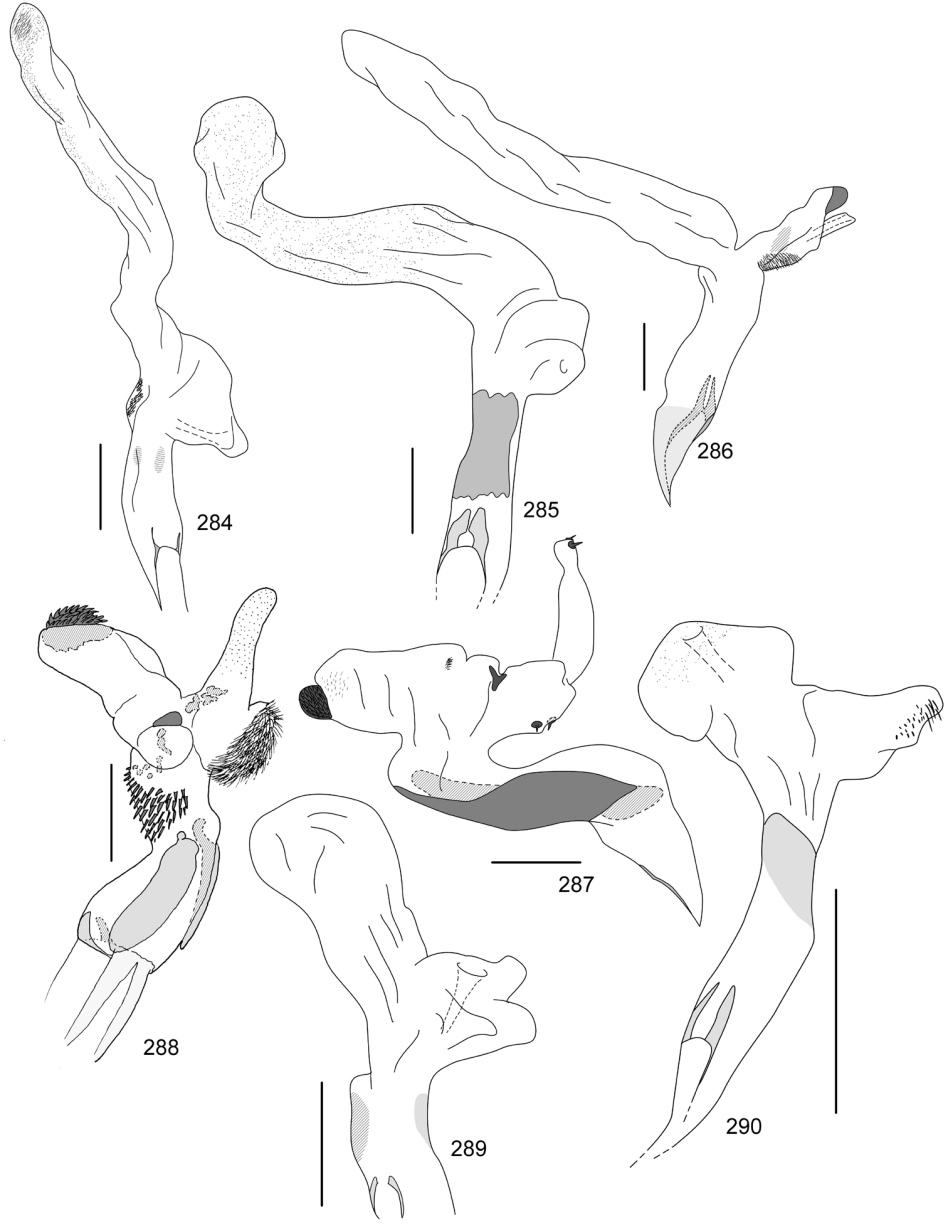
Endophallus of **284** *Anomala ruatana* (Playa Naranjo, Guanacaste, MNCR) **285** *Anomala semicincta* (Albergue Heliconias, Alajuela, CEUA) **286** *Anomala semilla* (Albergue Heliconias, Alajuela, CEUA) **287** *Anomala solisi* (Estación Pitilla, Guanacaste, MNCR) **288** *Anomala stillaticia* (Río Grande de Orosí, Cartago, MNCR) **289** *Anomala strigodermoides* (holotype) **290** *Anomala subaenea* (Estación Maritza, Guanacaste, MNCR). Scale bars: 1 mm. Figs 286–287 from Filippini et al. (2014); Fig. 289 from Filippini et al. (2015c); Fig. 288 from Filippini et al. (2015d).

**Figures 291–298.**
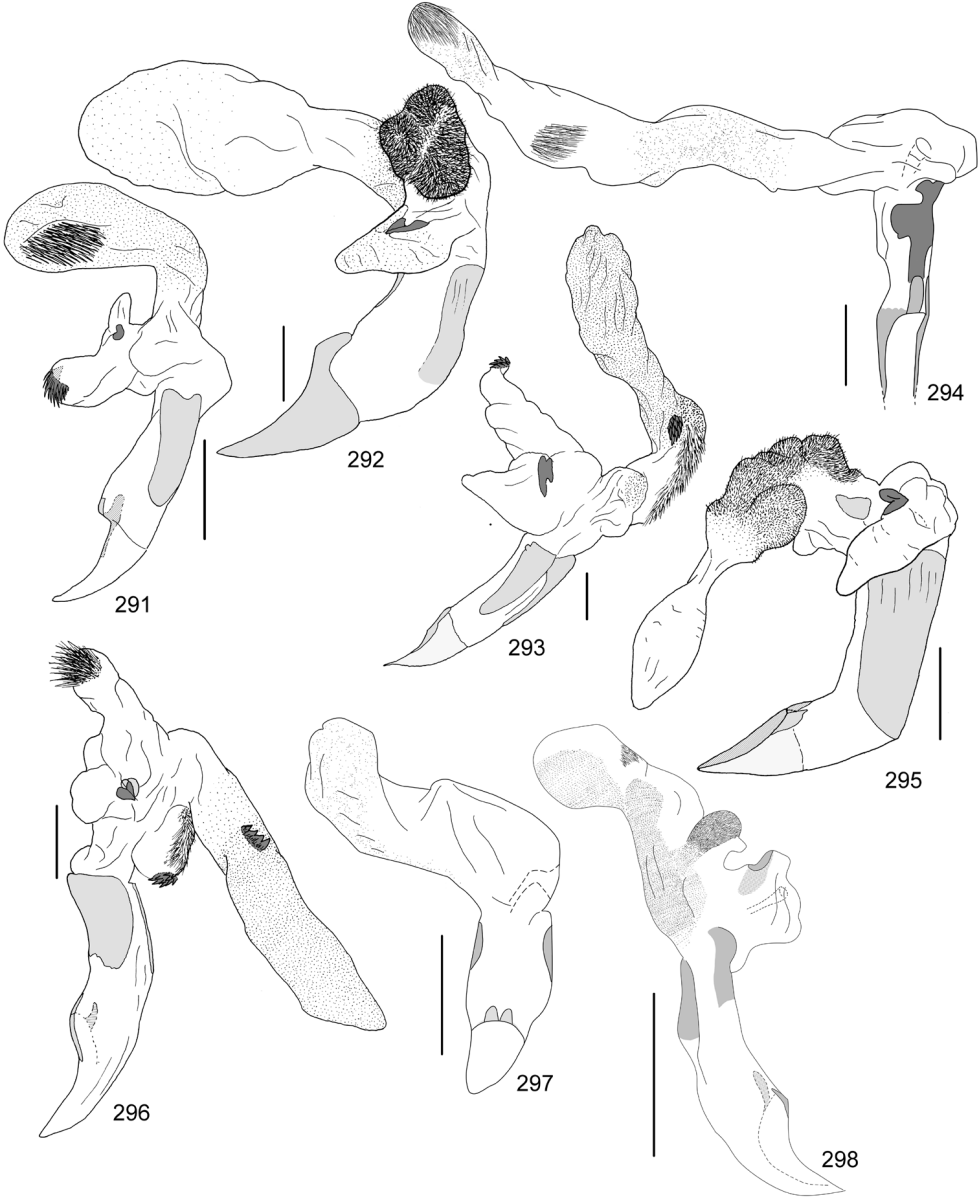
Endophallus of **291** *Anomala subridens* (Reserva Forestal Río Macho, Cartago, MNCR) **292** *Anomala subusta* (Estación Cacao, Guanacaste, MNCR) **293** *Anomala tenoriensis* (Parque Nacional Volcán Tenorio, Alajuela, MNCR) **294** *Anomala testaceipennis* (Vuelta Cmpana, Heredia, MNCR) **295** *Anomala trapezifera* (Fila Matama, Limón, MNCR) **296** *Anomala tuberculata* (Albergue Heliconias, Alajuela, CEUA) **297** *Anomala undulata* (Zarcero, Alajuela, MNCR) **298** *Anomala unilineata* (Parque Nacional Santa Rosa, Guanacaste, MNCR). Scale bars: 1 mm. Figs 298 from Filippini et al. (2015c); Figs 291–293, 295–296 from Filippini et al. (2015d).

**Figures 299–306.**
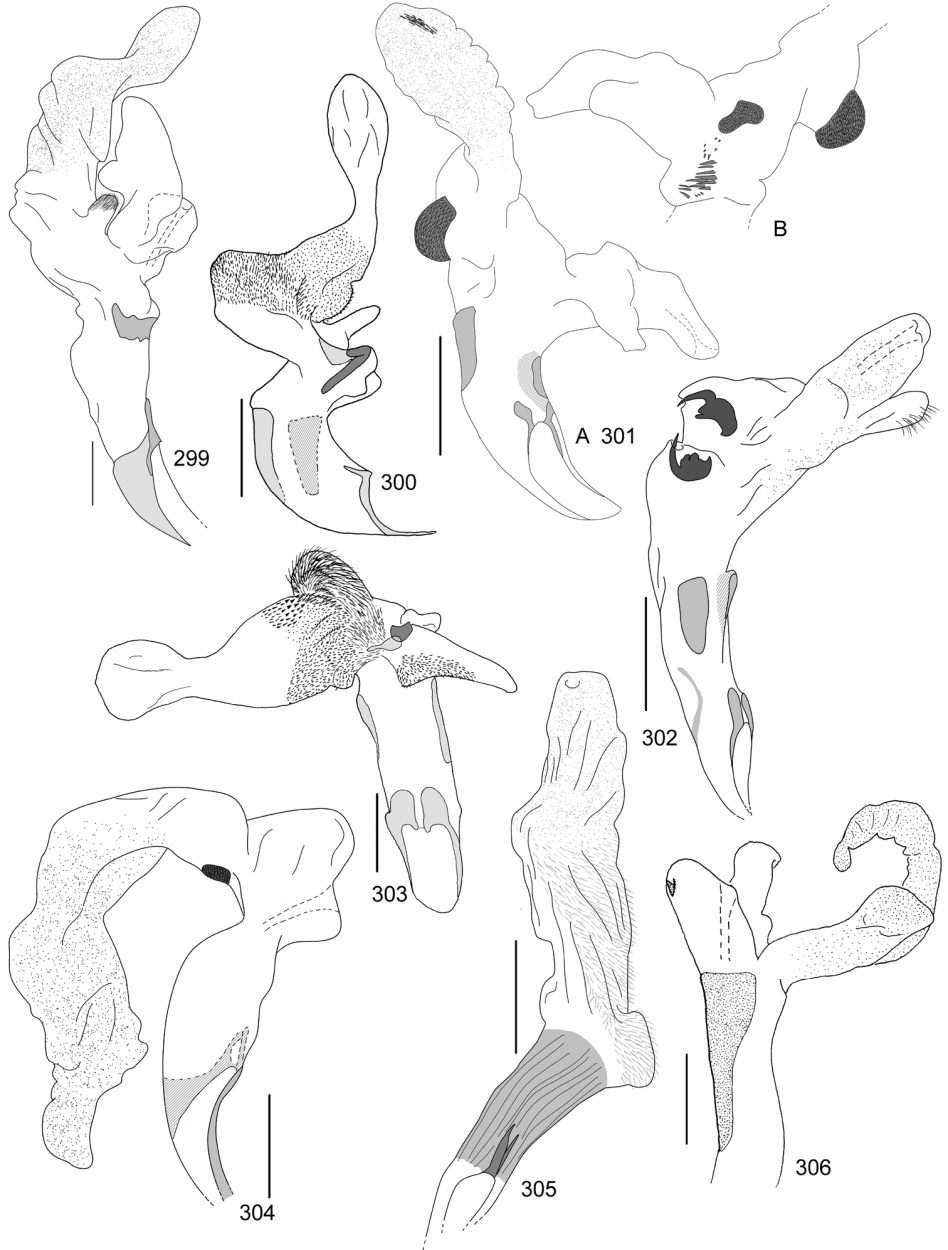
Endophallus of **299** *Anomala valida* (Estación Biológica La Selva, Heredia, MNCR) **300** *Anomala vallisneria* (Sector Las Pailas, Guanacaste, MNCR) **301** *Anomala veraecrucis*, A and B: oppsite lateral views (Finca Jenny, Guanacaste, MNCR) **302** *Anomala volsellata* (Las Quebraditas, Puntarenas, MNCR) **303** *Anomala vulcanicola* (San Gerardo de Dota, San José, MNCR) **304** *Anomala zumbadoi* (Rancho quemado, Puntarenas, MNCR) **305** *Anomalorhina turrialbana* (Cabanga, Alajuela, CEUA) **306** *Callistethus calonotus* (Alto de Las Moras, Puntarenas, MNCR). Scale bars: 1 mm. Figs 302, 304 from Filippini et al. (2014); Fig. 306 modified from Filippini et al. (2015a); Figs 300, 303 from Filippini et al. (2015d).

**Figures 307–314.**
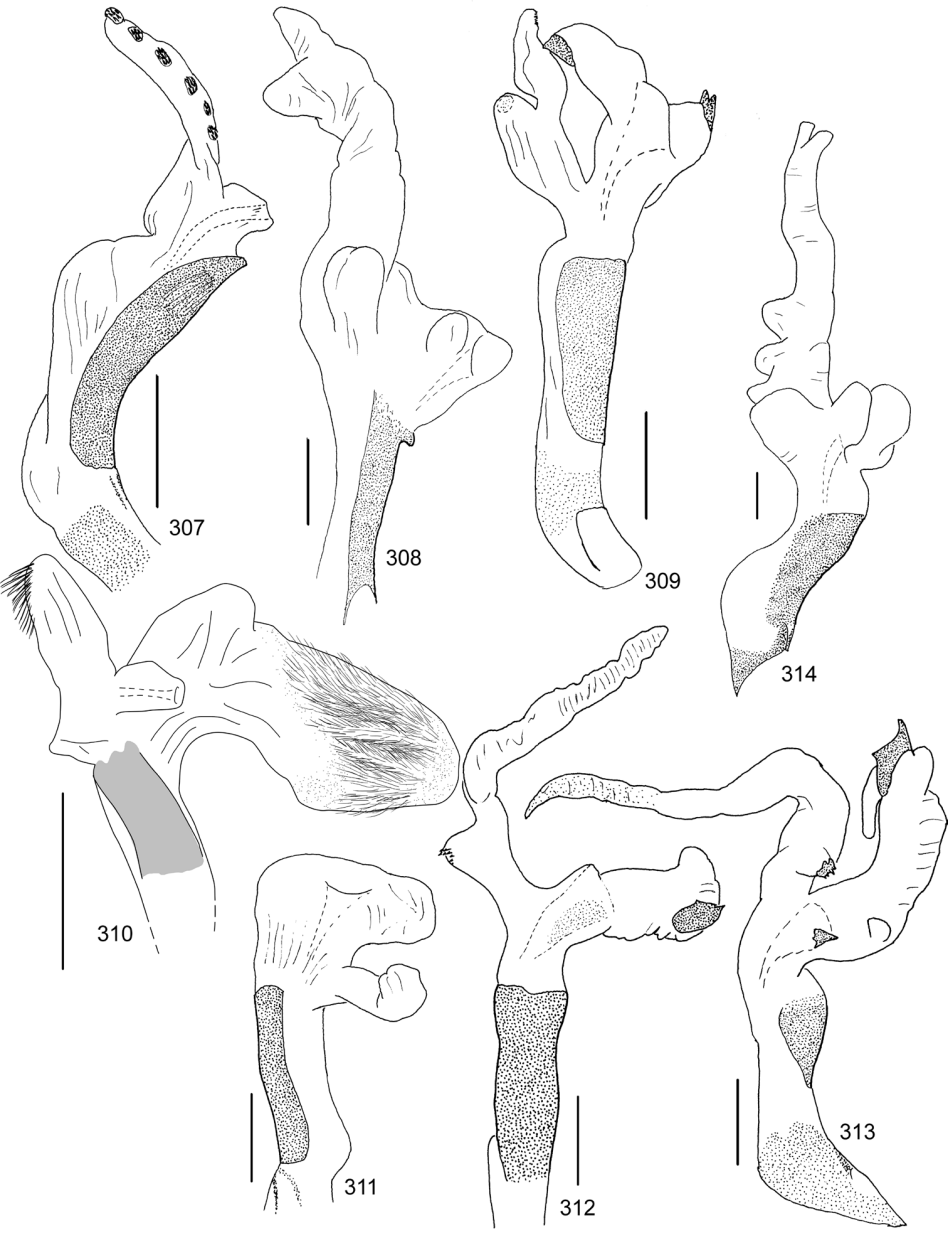
Endophallus of **307** *Callistethus carbo* (Río San Lorenzo, Guanacaste, MNCR) **308** *Callistethus chlorotoides* (Estación Hitoy Cerere, Limón, MNCR) **309** *Callistethus chontalensis* (El Copal, Cartago, CEUA) **310** *Callistethus chrysomelinus* (Buen Amigos, Puntarenas, MNCR) **311** *Callistethus flavodorsalis* (Finca Cafrosa, Puntarenas, MNCR) **312** *Callistethus fuscorubens* (Estación Altamira, Puntarenas, MNCR) **313** *Callistethus granulipygus* (Estación Quebrada Bonita, Puntarenas, MNCR) **314** *Callistethus jordani* (Estación Cacao, Guanacaste, MNCR). Scale bars: 1 mm. Figs 307–309, 311–314 modified from Filippini et al. (2015a).

**Figures 315–321.**
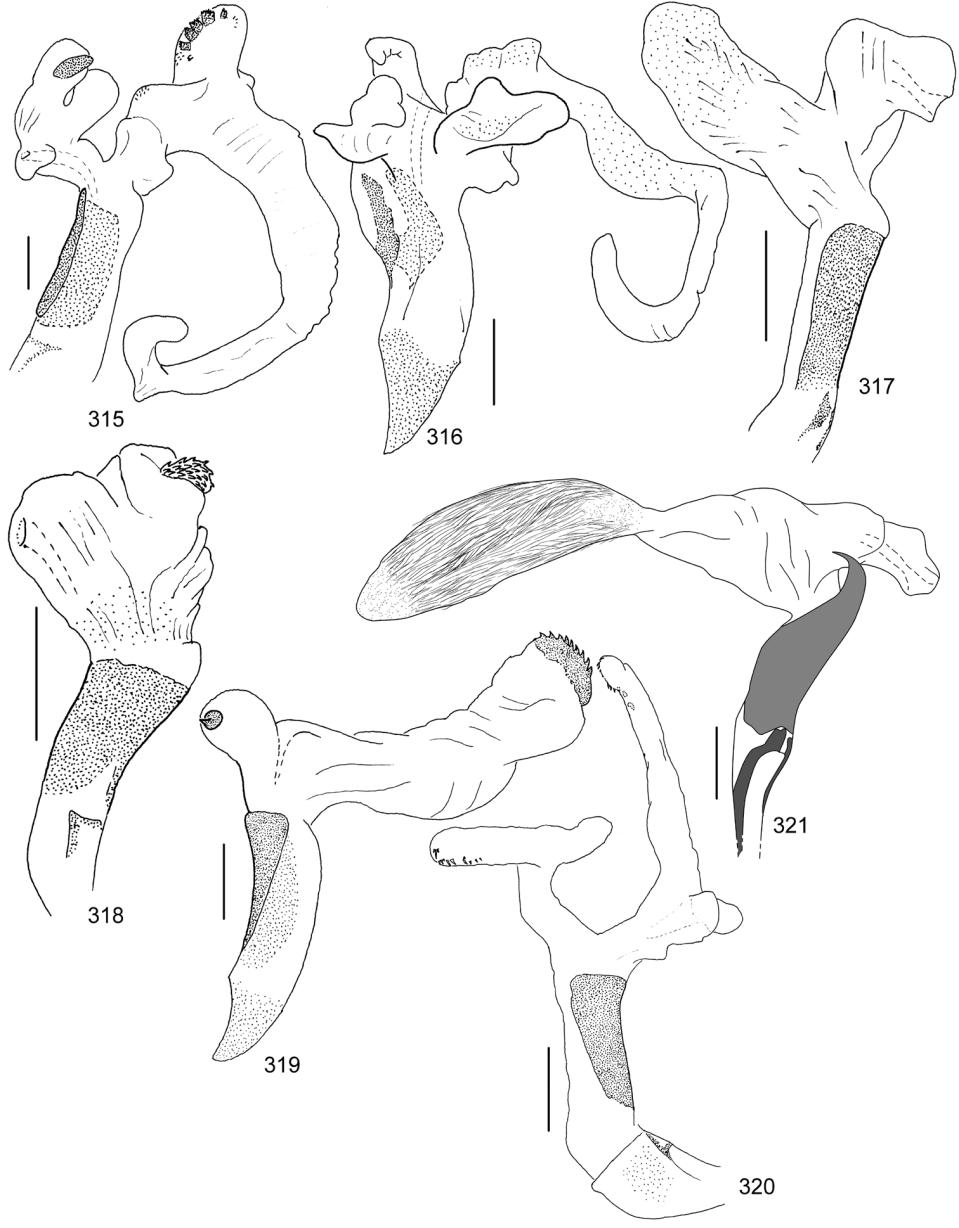
Endophallus of **315** *Callistethus lativittis*, sacculi artificially separated (Albergue Heliconias, Alajuela, CEUA) **316** *Callistethus levigatus* (Quebrada Segunda, Cartago, MNCR) **317** *Callistethus macroxantholeus* (Río San Lorencito, Alajuela, MNCR) **318** *Callistethus microxantholeus* (Est. Pitilla, Guanacaste, MNCR) **319** *Callistethus mimeloides* (Orosilito, Guanacaste, CEUA) **320** *Callistethus multiplicatus* (Sector Cerro Cocori, Limón, MNCR) **321** *Callistethus nicoya* (Estación Quebrada Bonita, Puntarenas, MNCR). Scale bars: 1 mm. Figs 315–320 modified from Filippini et al. (2015a).

**Figures 322–329.**
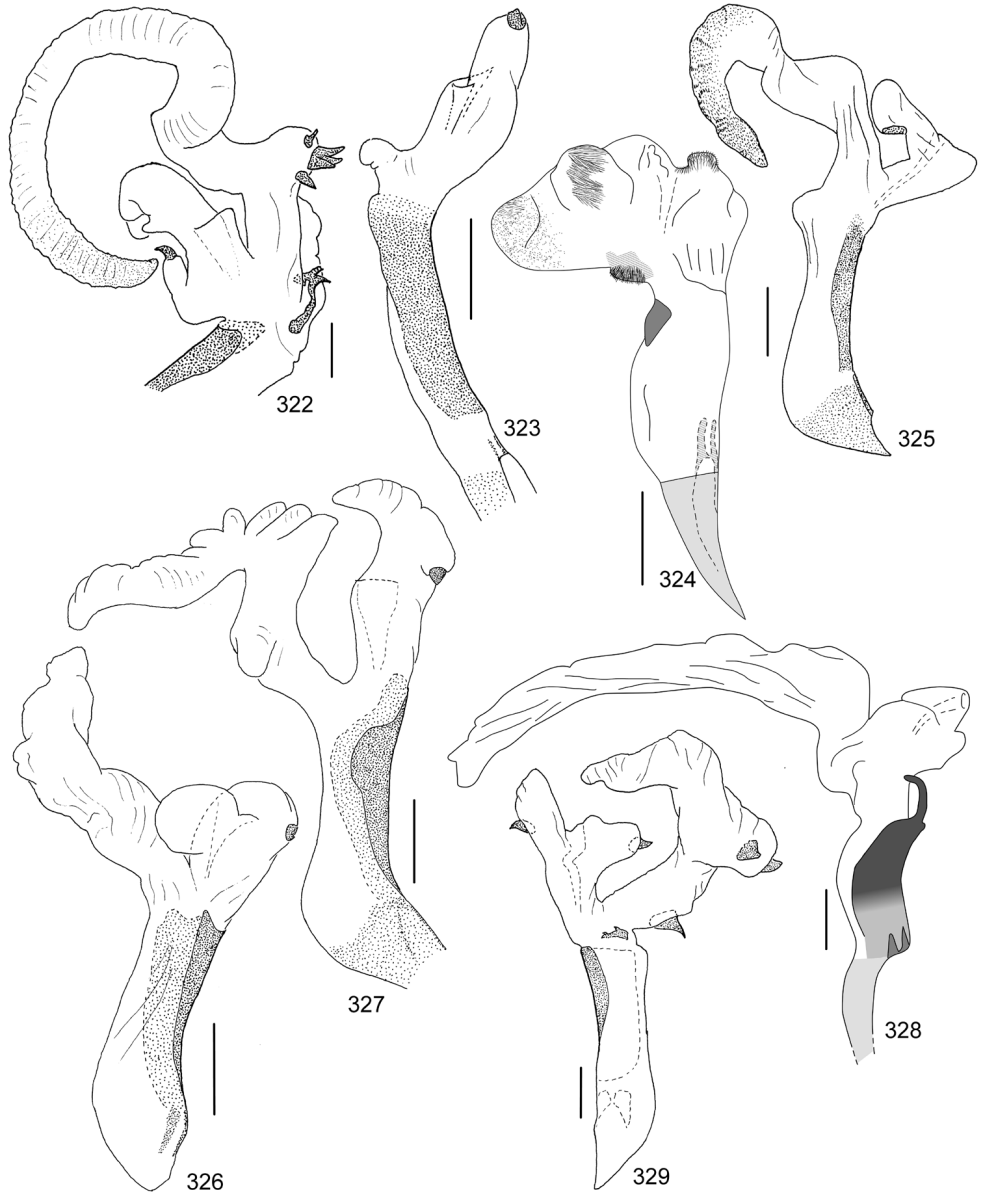
Endophallus of **322** *Callistethus parapulcher* (Estación Pittier, Puntarenas, MNCR) **323** *Callistethus pseudocollaris* (Estación La Casona, Puntarenas, MNCR) **324** *Callistethus ruteloides* (holotype) **325** *Callistethus schneideri* (Albergue Heliconias, Alajuela, MNCR) **326** *Callistethus specularis*, sacculi artificially separated (Quebrada Segunda, Cartago, MNCR) **327** *Callistethus stannibractea* (Estación Barva, Heredia, MNCR) **328** *Callistethus sulcans* (Estación La Maritza, Guanacaste, MNCR) **329** *Callistethus valdecostatus* (Estación Biológica Las Alturas, Puntarenas, MNCR). Scale bars: 1 mm. Figs 322–323, 325–327, 329 modified from Filippini et al. (2015a); Fig. 324 from Filippini et al. (2015b).

**Figures 330–337.**
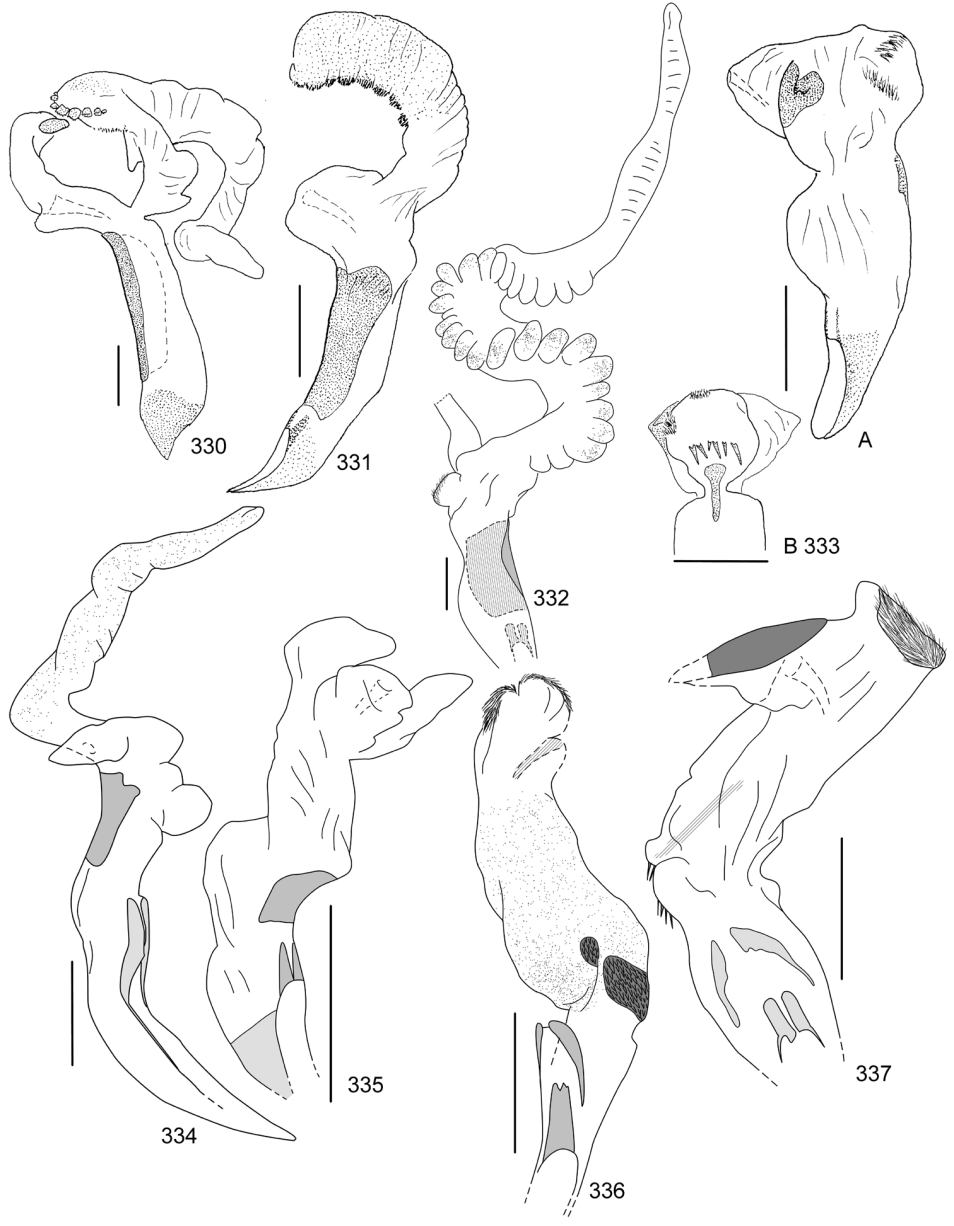
Endophallus of **330** *Callistethus vanpatteni* (Cinco Esquinas de Carrizal, Alajuela, MNCR) **331** *Callistethus xiphostethus* (Los Ángeles, Heredia, MNCR) **332** *Callistethus yalizo* (holotype) **333** *Moroniella nitidula*, A: lateral view, B: dorsal view (Zarcero, Alajuela, MNCR) **334** *Strigoderma auriventris* (Sector San Ramón de dos ríos, Alajuela, MNCR) **335** *Strigoderma biolleyi* (San Luis, Puntarenas, MNCR) **336** *Strigoderma nodulosa* (Estación Quebrada Bonita, Puntarenas, MNCR) **337** *Strigoderma sulcipennis* (Finca Jenny, Guanacaste, MNCR). Scale bars: 1 mm. Figs 330–331 modified from Filippini et al. (2015a); Fig. 332 from Filippini et al. (2015b).

**Figure 338.**
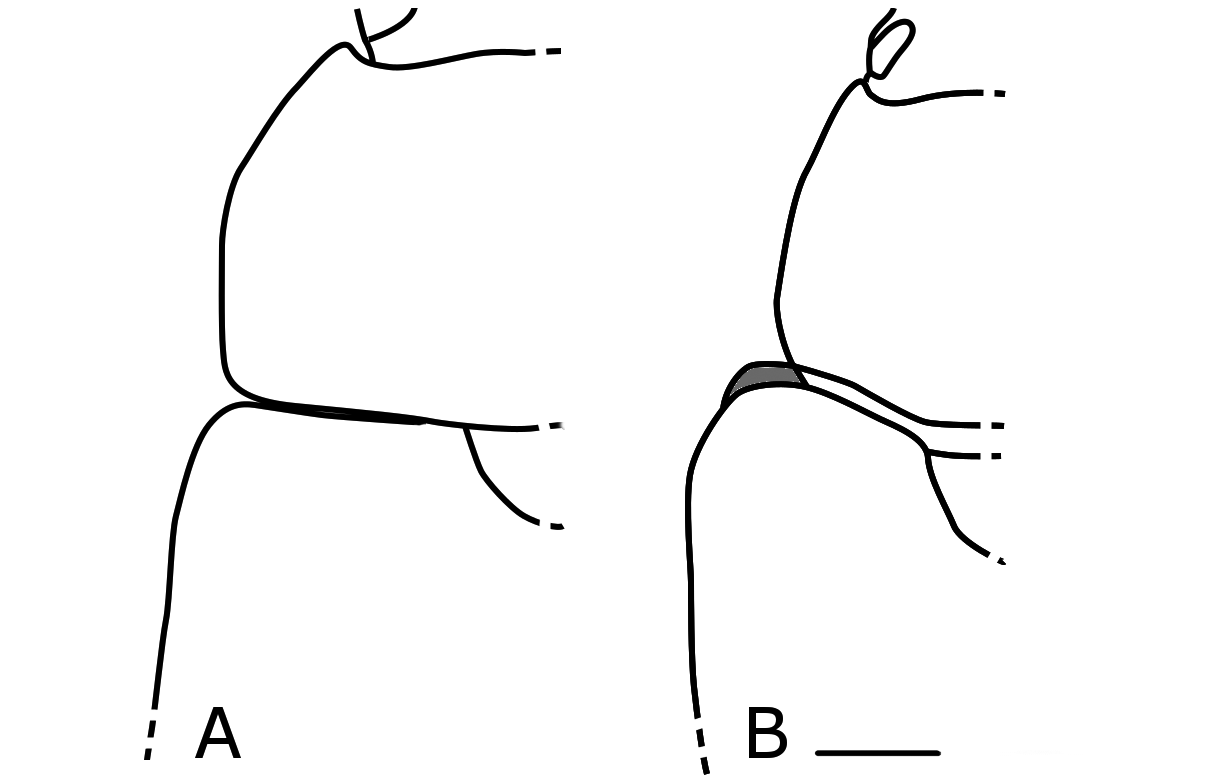
Mesepimeron of **A** *Anomalorhina turrialbana* **B** *Strigoderma nodulosa*. Scale bars: 1 mm.
